# Semisynthesis of homogeneous spike RBD glycoforms from SARS-CoV-2 for profiling the correlations between glycan composition and function

**DOI:** 10.1093/nsr/nwae030

**Published:** 2024-01-22

**Authors:** Farong Ye, Cheng Li, Feng-Liang Liu, Xinliang Liu, Peng Xu, Rong-Hua Luo, Wenping Song, Yong-Tang Zheng, Tianlei Ying, Biao Yu, Ping Wang

**Affiliations:** Center for Chemical Glycobiology, Frontiers Science Center for Transformative Molecules, Zhangjiang Institute for Advanced Study, School of Chemistry and Chemical Engineering, Shanghai Jiao Tong University, Shanghai 200240, China; MOE/NHC/CAMS Key Laboratory of Medical Molecular Virology, Shanghai Frontiers Science Center of Pathogenic Microorganisms and Infection, Shanghai Institute of Infectious Disease and Biosecurity, Shanghai Engineering Research Center for Synthetic Immunology, School of Basic Medical Sciences, Shanghai Medical College, Fudan University, Shanghai 200032, China; Key Laboratory of Animal Models and Human Disease Mechanisms of Chinese Academy of Sciences/Key Laboratory of Bioactive Peptides of Yunnan Province, Center for Biosafety Mega-Science, Kunming Institute of Zoology, Chinese Academy of Sciences, Kunming 650223, China; Center for Chemical Glycobiology, Frontiers Science Center for Transformative Molecules, Zhangjiang Institute for Advanced Study, School of Chemistry and Chemical Engineering, Shanghai Jiao Tong University, Shanghai 200240, China; State Key Laboratory of Bioorganic and Natural Products Chemistry, Center for Excellence in Molecular Synthesis, Shanghai Institute of Organic Chemistry, Chinese Academy of Sciences, Shanghai 200032, China; Key Laboratory of Animal Models and Human Disease Mechanisms of Chinese Academy of Sciences/Key Laboratory of Bioactive Peptides of Yunnan Province, Center for Biosafety Mega-Science, Kunming Institute of Zoology, Chinese Academy of Sciences, Kunming 650223, China; MOE/NHC/CAMS Key Laboratory of Medical Molecular Virology, Shanghai Frontiers Science Center of Pathogenic Microorganisms and Infection, Shanghai Institute of Infectious Disease and Biosecurity, Shanghai Engineering Research Center for Synthetic Immunology, School of Basic Medical Sciences, Shanghai Medical College, Fudan University, Shanghai 200032, China; Key Laboratory of Animal Models and Human Disease Mechanisms of Chinese Academy of Sciences/Key Laboratory of Bioactive Peptides of Yunnan Province, Center for Biosafety Mega-Science, Kunming Institute of Zoology, Chinese Academy of Sciences, Kunming 650223, China; MOE/NHC/CAMS Key Laboratory of Medical Molecular Virology, Shanghai Frontiers Science Center of Pathogenic Microorganisms and Infection, Shanghai Institute of Infectious Disease and Biosecurity, Shanghai Engineering Research Center for Synthetic Immunology, School of Basic Medical Sciences, Shanghai Medical College, Fudan University, Shanghai 200032, China; State Key Laboratory of Bioorganic and Natural Products Chemistry, Center for Excellence in Molecular Synthesis, Shanghai Institute of Organic Chemistry, Chinese Academy of Sciences, Shanghai 200032, China; Center for Chemical Glycobiology, Frontiers Science Center for Transformative Molecules, Zhangjiang Institute for Advanced Study, School of Chemistry and Chemical Engineering, Shanghai Jiao Tong University, Shanghai 200240, China; Shenzhen Research Institute of Shanghai Jiao Tong University, Shenzhen 518057, China

**Keywords:** peptide ligation, glycosylation, glycoprotein

## Abstract

Vaccines have been the primary remedy in the global fight against coronavirus disease 2019 (COVID-19). The receptor-binding domain (RBD) of the spike protein, a critical viral immunogen, is affected by the heterogeneity of its glycan structures and relatively low immunogenicity. Here, we describe a scalable synthetic platform that enables the precise synthesis of homogeneously glycosylated RBD, facilitating the elucidation of carbohydrate structure–function relationships. Five homogeneously glycosylated RBDs bearing biantennary glycans were prepared, three of which were conjugated to T-helper epitope (T_pep_) from tetanus toxoid to improve their weak immune response. Relative to natural HEK293-derived RBD, synthetic RBDs with biantennary *N*-glycan elicited a higher level of neutralising antibodies against SARS-CoV-2 in mice. Furthermore, RBDs containing T_pep_ elicited significant immune responses in transgenic mice expressing human angiotensin-converting enzyme 2. Our collective data suggest that trimming the *N*-glycans and T_pep_ conjugation of RBD could potentially serve as an effective strategy for developing subunit vaccines providing efficient protection.

## Teaser

Highly efficient semisynthetic procedure of homogeneously glycosylated RBDs was developed and further illuminated removal of sialic acids and trimming of N-glycans enhancing in vivo activity against SARS-CoV-2.

## INTRODUCTION

**Figure 1.**
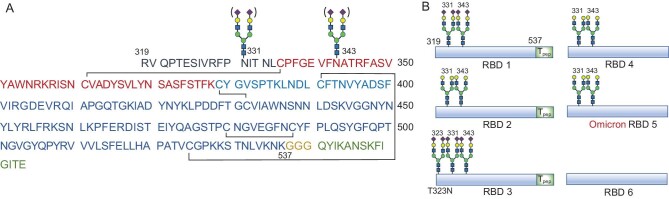
Structure of target homogeneous RBD glycoforms. (A) Homogeneous sequences of SARS-CoV-2 S protein RBD with two N-linked glycans (N331 and N343), linker and T_pep_. (B) Target RBDs (**1**–**4** and Omicron RBD **5**) bearing homogeneous glycans (N331 and N343) and nonglycosylated RBD **6**. A T323N mutation was introduced into RBD **3**, which was glycosylated. RBD (PDB code: 6WPS), receptor-binding domain. Linker, GGG. T_pep_, QYIKANSKFIGITE (Q830–E843 derived from tetanus toxoid). Omicron RBD (B.1.1.529, PDB code: 7QO7).

SARS-CoV-2 and its variants infect host cells via interactions between the receptor-binding domain (RBD) on the viral surface Spike (S) glycoprotein and human receptor angiotensin-converting enzyme 2 (ACE2) [1–3]. Accordingly, RBD containing two conserved N-linked glycans (N331, N343) represents the primary target for vaccine development and neutralising antibodies (Fig. 1A) [4–6]. The two glycosylation sites are important for viral infectivity [7] and elicit an incomplete neutralisation effect by producing glycan shields, thus impacting the immunogenic integrity of the vaccine [7,8]. To date, several glycosylated RBD-based subunit vaccines produced using various expression platforms have been evaluated in clinical trials or clinically approved, including yeast [9], insect [1,10], mammalian [11], and HEK293 cell systems expressing RBD [8]. Currently, vaccines are developed without consideration of the complex effects of glycosylation and the expression systems utilised result in highly heterogeneous RBD glycoforms. For instance, the *N*-glycosylation sites (N331 and N343) of RBD expressed from Chinese hamster ovary (CHO) and human embryonic kidney 293 (HEK293) cells contain mainly heterogeneous complex-type glycans with bi- and higher antennary structures as the major glycoforms [12], whereas their epitopes may be shielded by glycans. The microheterogeneity of these glycoforms often results in different pharmacodynamic and biological properties [13,14]. Notably, sialylated complex-type glycans of S protein are associated with enhanced viral infectivity and S protein vaccine lacking sialic acids and glycan shield is reported to induce improved protective responses against SARS-CoV-2 [8]. Therefore, comprehensive exploration of the specific effects of glycosylation on RBD-based vaccines is of paramount clinical importance. We previously reported the chemical synthesis of asialylated glycoforms of RBDs and their binding profiles with ACE2 and neutralising antibodies [15]. However, effective methods of preparation of homogeneous sialylated RBDs and antigenicity studies are yet to be developed. In this context, attainment of a well-defined glycosylated RBD, which is beyond the reach of current capabilities, could play a critical role in clarifying the relationship between degree of glycosylation of vaccines and immune response, providing a novel mimic epitope and rational route for effective vaccine design.

Recombinant subunit vaccines usually elicit a weak immune response and require the participation of appropriate adjuvants [10,16]. Q830–E843 (QYIKANSKFIGITE, T-helper cell peptide, T_pep_) derived from the tetanus toxoid (TT) has been confirmed as a safe epitope for improving the immunogenicity of subunit vaccines in a commercial setting [17–19]. Accordingly, we were interested in introducing T_pep_ into glycosylated RBD-based vaccines, with the aim of enhancing the quality and quantity of subunit vaccine-promoted immunity. In addition, SARS-CoV-2 is continuously evolving as a result of genetic selection, leading to increased infectivity and immune escape. B.1.1.529, a highly transmissible coronavirus variant named Omicron with notably distinct biology from the original virus, has rapidly spread worldwide [20]. The extensive mutations of Omicron have induced antigenic shifting, immune evasion and a decrease in the protective potency of currently marketed vaccines, 15 of which are found in RBD. The collective factors, along with the rapid spread of the Omicron variant that can efficiently escape from clinically approved vaccines and monoclonal antibodies, further highlight the importance of developing RBD vaccines with well-defined structures that are capable of inducing strong immunity against SARS-CoV-2. In this study, we comprehensively investigated the immunogenicity and protective efficacy of chemically synthesised homogeneous glycosylated RBD-based vaccines (**1**–**5**) (Fig. 1B). The glycoforms **1**–**5** included sialylated and asialylated N-linked glycan at conserved N331 and N343, along with the T-helper cell epitope Q830–E843. Compared with recombinant HEK293 RBD and nonglycosylated RBD **6**, synthetic RBDs with trimmed biantennary *N*-glycans enhanced the neutralising antibody titer response. The collective data from *in vitro* and *in vivo* studies on the utility of RBD incorporating TT as vaccines against SARS-CoV-2 indicate that trimming complex *N*-glycans and introducing the TT peptide in RBD provides an efficient strategy to develop vaccine candidates with improved efficacy.

## RESULTS AND DISCUSSION

**Figure 2.**
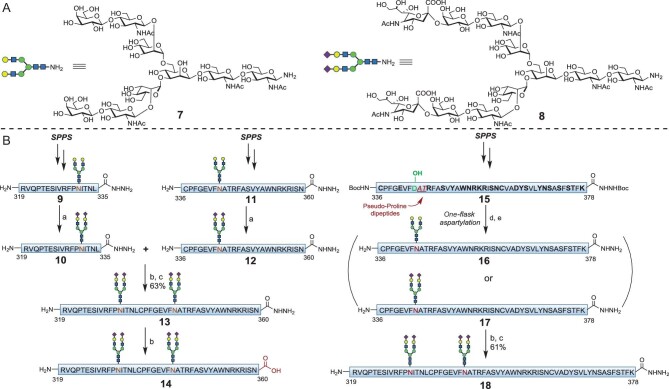
Preparation of glycosylated RBD fragments. (A) Structures of glycosyl amines **7** and **8** employed in the preparation of glycopeptides. Nonasaccharide **7**, undecasaccharide **8**. (B) Preparation of glycosylated RBD fragments. Reaction conditions: (a) sialylation, Neu5Ac (4.0 equiv), CTP (8.0 equiv), *PmST3* (0.30 mg·mL^–1^) and NmCSS (0.30 mg·mL^–1^), 100 mM Tris•HCl, 20 mM MgCl_2_, pH 8.6, 37°C, 10 h (83% for **10**, 76% for **12**); (b) peptide hydrazide activation, NaNO_2_ (6.0 equiv), 6 M GND•HCl, 200 mM Na_2_HPO_4_, pH 3.0, –15°C, 15 min; (c) NCL: MPAA (100 mM), 6 M GND•HCl, 200 mM Na_2_HPO_4_, pH 6.8, 25°C, 2 h (63% for **13**, 61% for **18**); (d) glycosyl amine **7** or **8** (1.5 equiv), HATU (10.0 equiv), DIPEA (20.0 equiv), DMSO, 25°C, 3 h; (e) Cocktail B (49% for **16**, 42% for **17** over two steps). Neu5Ac, *N*-acetylneuraminic acid. CTP, cytidine-5′-triphosphoric acid disodium salt. *PmST3*, α-2,6-sialyltransferase. NmCSS, CMP-sialic acid synthetase. GND•HCl, guanidine hydrochloride. AAs with protecting groups are shown in bold and pseudoproline dipeptides in red (underlined). The protecting groups are as follows: C(Trt), D(*t*Bu), E(*t*Bu), R(Pbf), S(*t*Bu), W(Boc), N(Trt), K(Boc), Y(*t*Bu), T(*t*Bu). HATU, O-(7-azabenzotriazol-1-yl)-*N,N,N* ′,*N*′-tetramethyluronium hexafluorophosphate; DIPEA, diisopropylethylamine; DMSO, dimethylsulfoxide; TFA, trifluoroacetic acid; Pbf, 2,2,4,6,7-pentamethyldihydrobenzofuran-5-sulfonyl group; Cocktail B, TFA/H_2_O/i-Pr_3_SiH/phenol (88 : 5 : 2 : 5, v/v/v/v); MPAA, 4-mercaptophenyl acetic acid.

Despite recent progress in protein synthesis [21–23] and oligosaccharide synthesis [24–26], chemical synthesis of sufficient quantities of medium-sized glycoproteins (>200 amino acids in length) containing complex oligosaccharides remains a tremendous challenge [27–30]. RBD (R319-K537) is a glycoprotein containing 219 amino acid residues organised as a five-stranded antiparallel β-sheet with four intramolecular disulfide linkages (C336–C361, C379–C432, C391–C525 and C480–C488). Previously, our group synthesised homogeneous glycoforms of RBD with asialo-*N*-glycans via expressed protein ligation (EPL) [31] using N360–C361 as the junction site [15]. We initially believed that the route to generating sialylated RBD glycoforms, if followed directly, could fulfil our synthetic goals. In this context, we prepared an N-linked glycopeptide RBD (319–360) fragment containing a terminal α-2,3 sialylated undecasaccharide at N331 and N343. Given that biantennary *N*-glycans are one of the conserved components in yeast, CHO and HEK293-expressed RBDs, we chose to incorporate biantennary *N*-glycans **7** and **8** at N331 and N343 in our envisioned synthetic RBD (Fig. 2A, details in Supporting Information, page S10) [32]. Enzymatic sialylation of glycopeptide **9** [15] in the presence of recombinant α-2,3-sialyltransferase *PmST3* from *Pasteurella multocida* [33] led to rapid generation of α-2,3-sialylated glycopeptide **10** with 83% isolated yield (Fig. 2B). Similarly, glycopeptide **11** [15] readily underwent sialylation with *PmST3*, generating α-2,3-sialylated RBD (336–360) fragment **12** with 76% yield. Using these glycopeptides, peptide hydrazide **10** was converted into thioester via *in situ* NaNO_2_ activation developed by Liu and coworkers [34], followed by coupling with glycopeptide **12** under native chemical ligation (NCL) conditions [35,36], providing an excellent isolated yield of RBD (319–360) fragment **13** (63%). Unfortunately, transformation of the C-terminal Asn hydrazide **13** to the corresponding peptide thioester, performed with the intention of preparing the NCL precursor, failed and resulted in completely hydrolysed product **14** (Supporting Information, page S16) [34]. Extensive investigations led to the speculation that the presence of sialic acids facilitates the hydrolysis of C-terminal Asn thioester, further highlighting the synthetic challenges related to preparation of sialylated complex glycoproteins.

Next, we rearranged the ligation site of EPL due to the difficult Asn–Cys junction. As shown in Fig. 1A, retrosynthesis of RBD was reorganised into two fragments: synthetic glycopeptide RBD (319–378) with two *N*-glycans at N331 and N343 and expressed RBD **19** (C379–K537–GGG–T_pep_) bearing an N-terminal Cys for crucial EPL and a C-terminal T-helper cell epitope for enhanced immune responses. Using this strategy, the Asn360–Cys361 junction was replaced with Lys378–Cys379. In addition, given the position of Cys residues and glycans, the glycopeptide (319–378) was further divided into two fragments, specifically, R319–L335 and C336–K378. Preparation of sialylated glycopeptide (C336–K378) commenced with synthesis of the protected peptide **15** in which Boc-hydrazide was induced to prevent undesired conjugation during subsequent HATU-assisted aspartylation. Generation of glycopeptide **16** was achieved through pseudoproline-mediated one-flask aspartylation [33,37,38]. Coupling of the carboxylic acid at D343 with glycosyl amine **7** produced **16** with a 49% isolated yield. However, incubation of **16** with CMP-Neu5Ac in the presence of α-2,3-sialyltransferase *PmST3* led to incomplete conversion, presumably due to the poor solubility of **16** in reaction buffer. Thus, following the pseudoproline-mediated one-flask aspartylation protocol, glycopeptide RBD (C336–K378) fragment **17** was prepared via conjugation of peptide **15** and sialylated glycosylamine **8** with a 42% isolated yield. The two sialylated glycopeptides **10** and **17** were coupled via NCL to generate RBD fragment (319–378). Using this strategy, the previously challenging N360–C361 junction was replaced with a new K378–C379 ligation site to facilitate the required EPL. Peptide hydrazide **10** was activated through NaNO_2_, generating the corresponding thioester. Following one-step NCL with glycopeptide **17**, RBD (319–378) fragment **18** bearing two sialylated *N*-glycans at N331 and N343 was readily obtained with a 61% isolated yield.

**Figure 3.**
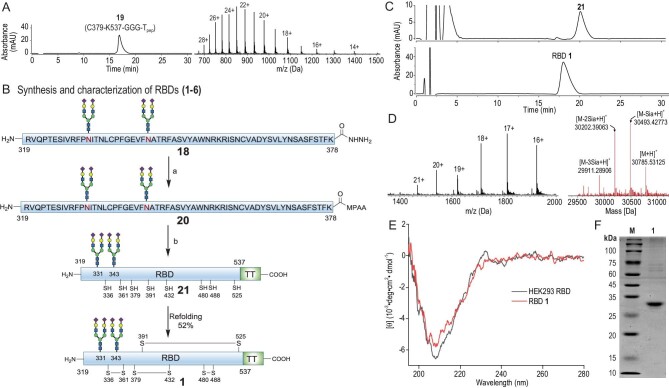
Synthesis and characterisation of RBD **1**. (A) Characterisation of recombinant fragment **19** (C379–K537–GGG–T_pep_) using HPLC and ESI-MS ([M + H]^+^ calcd, 19532.8). (B) Ligation and refolding conditions for glycosylated RBD **1**. Ligation conditions: (a) peptide hydrazide activation, NaNO_2_ (6.0 equiv), 6 M GND•HCl, 200 mM Na_2_HPO_4_, pH 3.0, –15°C, 15 min; (b) NCL, expressed RBD fragment (**19, 10** and **12**), MPAA (100 mM), 6 M GND•HCl, 200 mM Na_2_HPO_4_, pH 6.8, 25°C, 3 h (63% for **13**, 61% for **18**). Refolding protocol: Buffer A, 2 M GND•HCl, 50 mM Tris•HCl, 3 mM GSH, 0.9 mM GSSG, 400 mM Arg, pH 8.0, 4°C, 36 h. Buffer B, 1 M GND•HCl, 50 mM Tris•HCl, 3 mM GSH, 0.9 mM GSSG, 200 mM Arg, pH 8.0, 4°C, 24 h. Buffer C, 250 mM NaCl, 50 mM Tris•HCl, 3 mM GSH, 0.9 mM GSSG, 100 mM Arg, pH 8.0, 4°C, 24 h. Buffer D, 250 mM NaCl, 50 mM Tris•HCl, pH 8.0, 4°C, 12 h. (C) HPLC analysis of the refolding process and purified RBD **1**. (D) ESI-MS ([M + H]^+^ calcd, 30803.4) and HRMS of RBD **1** ([M + H]^+^ calcd, 30785.6; found, 30785.5, 0–4 sialic acids (Sia) were disconnected under HRMS conditions). (E) Circular dichroism spectrum of RBD **1**. (F) SDS-PAGE of RBD **1**. HEK293 RBD: ACROBiosystems, Arg319–Lys537 with His Tag, cat. no. SPD-C52H1.

The expressed RBD fragment **19** with T_pep_ (C379–K537–GGG–T_pep_) was cloned and produced efficiently in *Escherichia coli* (*E. coli*). The N-terminal methionine was removed simultaneously using endogenous methionine aminopeptidase [39]. Subsequently, the desired protein **19** was purified using high-performance liquid chromatography (HPLC) and obtained with high purity and yield of 23 mg/L (Fig. 3A). Having accomplished the preparation of prerequisitely expressed RBD fragment **19** and glycopeptide **18**, we performed their assembly using EPL to create the full RBD domain (Fig. 3B). Peptide thioester **20** was easily obtained from peptide hydrazide **18** through *in situ* NaNO_2_ activation, which set the stage for final EPL assembly. The projected EPL between **20** and expressed protein **19** proceeded with no issues, generating fully sialylated RBD **21** with a 52% isolated yield. The refolding process was conducted according to our previously reported protocol [15] in a dialysis tube using glutathione (GSH)/glutathione disulfide (GSSG) as the redox shuffling agent. RBD **21** was dialysed against refolding buffers A–D (Fig. 3B) and purified using size exclusion high-performance liquid chromatography, resulting in RBD **1** with a 52% isolated yield. Successful refolding of RBDs (**2**–**6**) was further validated based on the HPLC retention time (Fig. 3C), electrospray ionization mass spectrometry (ESI-MS), high-resolution MS (HRMS, Fig. 3D), circular dichroism (CD) criteria (Fig. 3E) and sodium dodecyl sulfate-polyacrylamide gel electrophoresis (SDS-PAGE) (Fig. 3F). Following the procedure established for RBD **1**, RBD **2** was synthesised using glycopeptides **9, 16** and expressed protein **19** as precursors. RBD **3** containing one mutated sialylated N-linked glycan (T323N) was generated efficiently with the same protocol as RBD **1** using glycopeptide **17, S11** (Supporting Information, page S36) and expressed protein **19** as precursors. The present method provides a practical strategy for enhancing the sialylation of RBD products. RBD **4** was prepared by our group in a previous study [15]. To generate RBD variants without T_pep_, Omicron RBD fragment (379–537) was expressed in *E. coli* (Supporting Information, page S48). Using a similar ligation and refolding protocol, Omicron RBD **5** bearing nonasaccharides at N331 and N343 was prepared with similar yields. Finally, to evaluate the impacts of N331, N343 glycan and T-cell epitopes, nonglycosylated RBD 319–537 was expressed in *E. coli* and refolded, producing nonglycosylated **6** containing Met at the N-terminus (Supporting Information, page S58). Interestingly, during the refolding process, a white precipitate was formed that resulted in a lower yield (29%), indicating that nonglycosylated RBD undergoes considerable aggregation. Successful refolding of RBDs (**2**–**6**) was confirmed with the aid of HPLC, CD, SDS-PAGE and high-resolution MS (Supporting Information, page S63).

**Figure 4.**
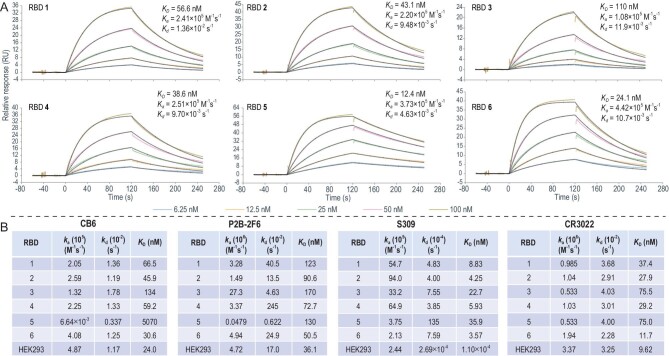
Binding assay of RBDs to ACE2 and mAbs. (A) Binding profile between RBDs and hACE2 (100, 50, 25, 12.5, 6.25 nM; 1 : 1 binding). (B) Binding profile between RBDs and mAbs (CB6, P2B-2F6, S309 and CR3022). HEK293 RBD: ACROBiosystems, Arg319–Lys537 with His Tag, cat. no. SPD-C52H1. ACE2: ACROBiosystems, cat. no. AC2-H5257.

To assess the impact of glycosylation of RBD proteins and evaluate the efficacy of synthetic RBDs as vaccine candidates (Fig. 4A), we further investigated the binding profile of RBD with ACE2 via surface plasmon resonance (SPR, Biacore) [1]. The dissociation constant (*K*_D_) of hACE2 and HEK293 RBD was 15.4 nM (Figure S84). Synthetic RBDs (**1**–**4** and **6**) showed reduced affinities, ranging from a 1.56- to 7.14-fold decrease. Our results demonstrate that synthetic RBD binds hACE2 with similar affinity, indicative of the correct conformation of the newly synthesised protein products. Notably, Omicron RBD **5** displayed enhanced binding affinity (1.24-fold) compared with HEK293 RBD. Direct comparison of RBD **4** and Omicron RBD **5** containing the same glycan showed that Omicron RBD **5** binds to ACE2 with increased affinity (by 3.11-fold), in line with previous reports [15]. Overall, compared with nonglycosylated RBD **6**, glycosylation, sialic acids and the T_pep_ motif induced a gradual slight decrease in binding affinity.

To further elucidate the specific functions of the different RBDs, dissociation constants (*K*_D_) of synthetic RBDs and four human neutralising antibodies (CB6 [5], P2B-2F6 [40], S309 [41] and CR3022 [6,42]) were measured using SPR (Fig. 4B). These antibodies are reported to target different COVID-19 S-RBD epitopes [43]. In general, the existence of sialic acid was associated with a trend of slight reduction in binding affinity (RBD **3, 1** and **2**), suggesting that sialylation enhances the glycan shielding effect against neutralising antibodies. T_pep_ exerted no effect on binding affinity of antibodies against RBDs **2** and **4**. Notably, mutation of Omicron RBD **5** resulted in diminished affinity for these antibodies relative to RBD **4**, ranging from a 1.79- to 85.6-fold reduction (CB6, 85.6; P2B-2F6, 1.79; S309, 6.05; CR3022, 2.57). CB6 specifically targets the receptor binding motif of SARS-CoV-2, indicating that extensive mutations of Omicron may result in strong potential resistance against this antibody. In keeping with previous findings [32], mutations in Omicron RBD led to a significant decrease in binding to CB6. The antibody S309, originally discovered by Corti and coworkers [41] and shown to have moderate neutralising activity against Omicron in a previous study [44], displayed reduced affinity (by 6.05-fold) for RBD **5** and **4** in our experiments. Compared to HEK293 RBD, reduced binding of RBDs (**1**–**6**) may be attributed to the lack of the important fucose epitope at N343 glycan. Antibodies P2B-2F6 and CR3022 against Omicron showed reduced affinities (ranging from 1.79- to 2.57-fold), indicating a lower effect against the variant.

### Development of RBD as a vaccine

**Figure 5.**
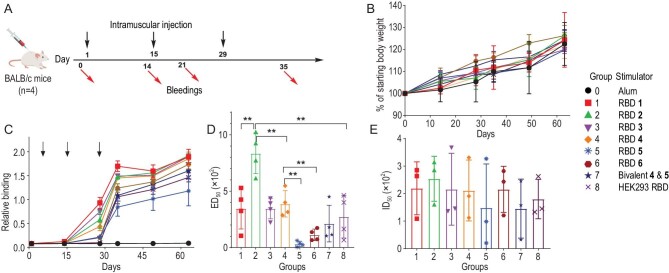
Assessment of synthetic RBDs as potential vaccines against SARS-CoV-2. (A) Immunisation schedule for BALB/c mice. (B) Relative body weight changes (*n* = 4). (C) Serum binding antibody response against RBD (PDB code: 6WPS) in mice on days 0, 14, 28, 35, 49 and 63 (*n* = 4). (D) Binding titers of plasma of vaccinees against His-tagged WT RBD. (E) Neutralising antibody titers against WT-pseudotyped SARS-CoV-2 virus (*n* = 3, day 35). ID_50_, 50% inhibitory dose. Statistically significant values (*P*) are presented as asterisks (*P < 0.05; **P < 0.01).

To gain a comprehensive insight into the functions of homogeneous glycosylated RBDs, we compared immunogenicity elicited by our vaccines. Nine groups of female BALB/c mice (*n* = 4) were used for experiments, including one unvaccinated group treated with phosphate-buffered saline (PBS) that served as the control (group 0), six vaccinated groups with RBDs **1**–**6** (groups 1–6), one vaccinated group with a mixture of RBDs (**4** and **5**, 1 : 1, group 7), and one vaccinated group with HEK293 RBD (group 8). RBDs (0.32 nmol in 50 μL) were formulated with aluminium hydroxide (alum, 50 μL) as an adjuvant and subsequently used to immunise 6- to 8-week-old female BALB/c mice via intramuscular injection. All mice in groups 1–9 were immunised on days 1, 15 and 29 (Fig. 5A). Serum samples collected on days 0, 14, 28, 35, 49 and 63 were evaluated for RBD-specific and neutralising antibody responses. No body weight loss was observed in immunised mice (Fig. 5B).

To evaluate the RBD-specific antibody response during the whole immunisation course, we measured relative binding of immunised serum from the nine groups to RBD (1 : 100 dilution) via enzyme-linked immunosorbent assay (ELISA) at six time-points. On day 14 after the primary vaccination, we observed slight RBD-specific antibody responses elicited by RBD and a significantly higher level of binding antibodies by days 35 and 63 (Fig. 5C). For example, mice immunised with RBD **1** exhibited the highest binding antibody response relative to those immunised with other RBDs, which was significantly increased (∼7.7- to 14.1-fold) on days 28 and 35 after the second and third vaccinations. To further compare the RBD-binding antibody titers elicited by these vaccines, we assessed the serum 50% effective dose (ED_50_) to SARS-CoV-2 RBD on day 35 when antibody binding reached relatively high levels. ED_50_ of serum elicited by synthetic homogeneous glycosylated RBDs **1**–**4** with T_pep_ and biantennary glycans was higher than that by HEK293 RBD on day 35 (Fig. 5D), indicating that trimming of the glycan shield led to improved antibody responses. The Omicron RBD **5**-immunised group of mice demonstrated a lower level of antibody binding compared with HEK293 RBD, which could be attributed to antigen heterogeneity compared with the wild-type (WT) group. Moreover, RBD **6** and the bivalent RBD immunogen (**4** and **5**, group 7) induced similar levels of binding antibodies relative to the HEK293 RBD group. Among the synthetic homogeneous glycosylated RBDs, RBD **2** elicited a significant 3.1-fold higher titer of RBD-specific IgG relative to HEK293 RBD. The impact of synthetic RBDs was further determined using the pseudovirus neutralisation assay, an effective quantitative method for assessing neutralisation of SARS-CoV-2 (Fig. 5E) [11,45]. RBD **2** induced ID_50_ (254) on day 35, which was slightly higher than that of other RBDs. RBDs **1, 3, 4** and **6** elicited similar ID_50_ titers whereas slightly lower ID_50_ titers were obtained with RBDs **5, 7** and **8**. Based on the collective findings, we conclude that RBDs (**1** and **2**) are potentially more immunogenic against the prototype SARS-CoV-2 strain.

**Figure 6.**
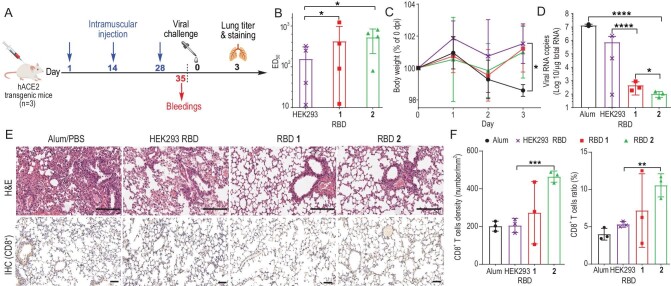
RBD (**1** and **2**) vaccination in hACE2 transgenic mice elicits enhanced protection efficacy against WT SARS-CoV-2 infection *in vivo*. (A) The immunisation schedule for hACE2 transgenic mice is depicted. (B) Sera collected on day 35 after vaccination were examined for RBD-specific antibodies (*n* = 3). (C) Body weights measured in hACE2 transgenic mice after the WT SARS-CoV-2 challenge (*n* = 3). (D) Virus titers in lungs of challenged hACE2 mice (*n* = 3). (E) Representative histopathology and immunohistochemistry of infected mouse lungs (3 dpi). First line: Hematoxylin and eosin (H&E) staining, scale bar 200 μm. Second line: immunohistochemical (IHC) staining for detection of CD8^+^ T cells; scale bar, 50 μm. (F) CD8^+^ T-cell density and ratio of lung tissue from authentic SARS-CoV-2-infected hACE2 mice (*n* = 3). hACE2 transgenic mice: GemPharmatech, cat. no. T037630. dpi: days post-infection. Statistically significant values (*P*) are presented as asterisks (*P < 0.05; **P < 0.01; ***P < 0.001; ****P < 0.0001). Differences were considered statistically significant at *P* < 0.05.

Transgenic mice expressing hACE2 represent a suitable animal model for the evaluation of vaccines [46,47]. To examine the protective efficacy of synthetic RBDs *in vivo*, we deployed the WT SARS-CoV-2 strain (Accession No. NMDCN0000HUI, China National Microbiology Data Center) challenge in hACE2 transgenic mice (*n* = 3) immunised with three injections of HEK293 RBD, RBD **1, 2** or placebo (alum/PBS on days 1, 14 and 28). Prechallenge sera were evaluated for RBD-specific antibody titers (Fig. 6A).

Notably, as shown in Fig. 6B, ED_50_ titers of RBD-specific antibodies induced by RBD **1** and **2** were 425 and 527, respectively, which were significantly higher than that elicited by HEK293 RBD (156). After intranasal challenge of mice with 1 × 10^6^ TCID_50_ of WT SARS-CoV-2, no weight loss was detected in prophylactically treated animals (Fig. 6C). Mice were euthanised at 3 days post-infection and the harvested lung tissues examined for viral RNA load, histopathology and T-cell response. In line with antibody titers, all RBD vaccinations reduced viral RNA loads. Significantly reduced virus titers were observed in mice immunised with RBD **1** (1644-fold) and **2** (7168-fold) relative to the HEK293 RBD immunogen (Fig. 6D). We further evaluated the protective effect of synthetic RBDs in terms of minimising lung pathology after challenge. Lung tissues from placebo mice exhibited mild inflammatory cellular infiltration, thickened alveolar walls and haemorrhage. In contrast, few lesions were observed in mice vaccinated with HEK293 RBD and no obvious lesions present in mice immunised with RBD **1** and **2** (Fig. 6E). We further evaluated the degree of CD8^+^ T cell distribution via immunohistochemistry based on reports that CD8^+^ T cell responses contribute to the protective efficiency of the vaccine against SARS-CoV-2 [48,49]. All immunised mice showed high levels of CD8^+^ T cells compared with the placebo group. Markedly enhanced CD8^+^ T cell contents were detected in groups vaccinated with synthetic RBDs including T_pep_ (**1**, 1.3-fold and **2**, 2.3-fold) compared with HEK293 RBD (Fig. 6F). The effect of T_pep_ on inducing the CD8^+^ T cell response was significant, especially in RBD **2**. Our results suggest that trimmed and asialylated glycoforms of RBDs elicit a higher level of antibody response than HEK293 RBD containing triantennary and tetrabiantennary glycans. Based on the collective findings, we conclude that RBD **2** serves as a powerful immunogen against SARS-CoV-2 with significantly enhanced immunogenicity. However, further comprehensive analysis of the immune response is warranted in future studies.

## CONCLUSION

In conclusion, we have developed a practical and efficient semisynthetic procedure for the production of RBDs bearing biantennary sialylated glycans. This protocol can achieve generation of complex glycoproteins in sufficient quantities and thus rapidly provide important materials for structural and functional studies of glycoproteins. Production of glycoproteins with well-defined structures is a valuable accomplishment at the level of glycoprotein synthesis, which includes a combination of oligosaccharide, peptide and protein chemistry. Glycosylation of RBD has a key influence on viral infectivity and immune responses. The HEK293 RBD contained biantennary, triantennary and tetraantennary glycans with higher sialylation levels as the major glycans, which could influence antibody recognition via shielding specific epitopes. Compared with HEK293 RBD, our synthetic RBD **2** with biantennary glycans and T_pep_ exhibited enhanced *in vivo* activity against SARS-CoV-2. Our collective data suggest that removal of sialic acids and trimming of *N*-glycans lead to increased antibody reactivity. Overall, the protocol reported in this study presents a promising strategy for the development of effective therapeutics and vaccines for several other pathogens, including SARS and human herpes virus. Ambitious synthesis strategies may therefore be employed in more complex disease-relevant glycoproteins, which will be disclosed in due course.

## Supplementary Material

nwae030_Supplemental_File
