# Supplementary material for: Semisynthesis of homogeneous spike RBD glycoforms from SARS-CoV-2 for profiling the correlations between glycan composition and function
Source: Natl Sci Rev. 2024 Jan 22;11(2):nwae030. doi: 10.1093/nsr/nwae030 (PMC10852988; doi:10.1093/nsr/nwae030)
Supplement: nwae030_Supplemental_File [file nwae030_supplemental_file.docx]

**Supporting Information**

**Semisynthesis of homogeneous spike RBD glycoforms from SARS-CoV-2 for profiling the correlations between glycan composition and function**

Farong Ye^1,†^, Cheng Li^2,†^, Feng-Liang Liu^3,†^, Xinliang Liu^1^, Peng Xu^4^, Rong-Hua Luo^3^, Wenping Song^2^, Yong-Tang Zheng^3,*^, Tianlei Ying^2,*^, Biao Yu^4^ and Ping Wang^1,5,*^

^1^Center for Chemical Glycobiology, Frontiers Science Center for Transformative Molecules, School of Chemistry and Chemical Engineering, Zhangjiang Institute for Advanced Study, Shanghai Jiao Tong University, Shanghai 200240, China

^2^MOE/NHC/CAMS Key Laboratory of Medical Molecular Virology, Shanghai Frontiers Science Center of Pathogenic Microorganisms and Infection, Shanghai Institute of Infectious Disease and Biosecurity, Shanghai Engineering Research Center for Synthetic Immunology, School of Basic Medical Sciences, Shanghai Medical College, Fudan University, Shanghai 200032, China

^3^Key Labora[tory of Animal Models and Human Disease Mechanisms of Chinese Academy of Sciences/Key Laboratory of Bioactive Peptides of Yunnan Province, Center for Biosafety Mega-Science, Kunming Institute of Zoology, Chinese Academy of Sciences, Kunming, Yunnan 650223, China.

^4^State Key Laboratory of Bioorganic and Natural Products Chemistry, Center for Excellence in Molecular Synthesis, Shanghai Institute of Organic Chemistry, Chinese Academy of Sciences, 345 Lingling Road, Shanghai 200032, China

^5^Shenzhen Research Institute of Shanghai Jiao Tong University, Shenzhen, 518057, China

†These authors contributed equally to this work.

*Corresponding authors. Email: zhengyt@mail.kiz.ac.cn; tlying@fudan.edu.cn; wangp1@sjtu.edu.cn.

Table of contents

[I. Chemical synthesis and characterization 3](#_Toc128443663)

[1. General information of reagents and instruments 3](#_Toc128443664)

[2. General Procedures 5](#_Toc128443665)

[2.1 General Procedure A for manual solid-phase peptide synthesis (SPPS) 5](#_Toc128443666)

[2.2 General procedure B for protein expression and purification 7](#_Toc128443667)

[2.3 General procedure C for enzymatic sialylation 7](#_Toc128443668)

[2.4 General Procedure D for conversion of glycopeptide hydrazide to glycopeptide thioester and 1^st^ NCL in one pot 8](#_Toc128443669)

[2.5 General Procedure E for conversion of glycopeptide hydrazide to glycopeptide thioester and 2^nd^ NCL in one pot 8](#_Toc128443670)

[2.6 General Procedure F for protein refolding and SEC-HPLC purification procedure 9](#_Toc128443671)

[2.7 General Procedure G for surface plasmon resonance (SPR) analysis 10](#_Toc128443672)

[3. Synthesis, purification and characterization of RBD **1** 10](#_Toc128443673)

[3.1 Enzymatically sialylated of nonasaccharide and nonasaccharide modified peptides 10](#_Toc128443674)

[3.2 Sialylated glycopeptide and RBD **1** synthesis and characterization with alternative method 22](#_Toc128443675)

[4. Synthesis, purification and characterization of RBD **2** 31](#_Toc128443676)

[5. Synthesis, purification and characterization of RBD **3** 36](#_Toc128443677)

[6. Refolding, purification and characterization of RBD **4** 47](#_Toc128443678)

[7. Synthesis, purification and characterization of RBD **5** 48](#_Toc128443679)

[8. Refolding, purification and characterization of naked RBD **6** 58](#_Toc128443680)

[9. CD and SDS-PAGE analysis of different RBD derivatives. 63](#_Toc128443681)

[10. Binding affinities of the RBD derivatives to antibodies and hACE2 receptor 64](#_Toc128443682)

[II. Immunological evaluation 68](#_Toc128443683)

# I. Chemical synthesis and characterization

## 1. General information of reagents and instruments

**1.1 Chemical reagents**

Technical solvents including dichloromethane (DCM), methanol (MeOH), tetrahydrofuran (THF), diethyl ether, N,N-dimethyl formamide (DMF) and chemical reagents including guanidine hydrochloride (GND•HCl), tris(2-carboxyethyl) phosphine (TCEP) and 4-(carboxymethyl) thiophenol (MPAA), acetylacetone, reduced glutathione (GSH), oxidized glutathione (GSSG), piperidine were purchased from Adamas-beta®, Bidepharm and Sinophram, all used as received, without further purification. HPLC grade acetonitrile (ACN), trifluoroacetic acid (TFA) and formic acid (FA) were purchased at the highest commercial quality from Macklin. Anhydrous dimethylsulfoxide (DMSO), N,N-Diisopropylethylamine (DIPEA) and O-(7-azabenzotriazol-1-yl)-N,N,N’,N’-tetramethyluronium hexafluorophosphate (HATU) used in coupling reaction and N-acetylneuraminic acid (Neu5Ac) and Cytidine-5'-triphosphoric acid disodium salt (CTP) used in enzymatic reactions were provided by Energy Chemical. Trityl chloride-ChemMatrix^®^ resins, Fmoc-protected amino acids (Fmoc-AA-OH), Boc-protected amino acids (Boc-AA-OH), pseudoproline dipeptides (Fmoc-Ile-Thr(Ψ^Me,Me^Pro)-OH, Fmoc-Ala-Thr(Ψ^Me,Me^pro)-OH, Fmoc-Ile-Ser(Ψ^Me,Me^pro)-OH, Fmoc-Glu(O*t*Bu)-Ser(Ψ^Me,Me^pro)-OH and Fmoc-Tyr(*t*Bu)-Ser(Ψ^Me,Me^pro)-OH), N,N′-Diisopropylcarbodiimide (DIC), ethyl cyanoglyoxylate-2-oxime (Oxyma) and other reagents for solid phase peptide synthesis (SPPS) were obtained from GL Biochem. His-Tagged HEK293 RBD (SARS-CoV-2 S protein RBD (Arg319-Lys537) expressed from human 293 cells (HEK293), Cat. No. SPD-C52H3) and human ACE2 protein (Gln18-Ser740, Fc Tag (Pro100-Lys330), expressed from human 293 cells (HEK293), Cat. No. AC2-H5257) were provided by ACRO Biosystems. The expression plasmids pET21a-rbd, pET21a-rbd-tt, pET21a-omicron-rbd, pET21a-naked rbd and pACYC-MAP were constructed by Sangon Biotech and GENEWIZ. CB6 (PDB entry 7C01), P2B2F6 (PDB entry 7BWJ), S309 (PDB entry 6WS6) and CR3022 (PDB entry 6W41) antibodies were provided by Prof. Tianlei Ying.

**1.2 Instruments and methods**

Reactions were set up under nitrogen (N_2_) atmosphere and monitored by Merck pre-coated thin layer chromatography (TLC) plates (silica gel 60 HSGF254, 0.2 mm) or LC-MS. Analytical TLC was visualized under UV light (254 nm and 365 nm), or stained by a solution of H_2_SO_4_ in MeOH (5%) or phosphomolybdic acid in EtOH (5%). Silica gel column chromatography purification was performed on silica gel GF254 (300−400 mesh).

Analytical HPLC-MS was recorded on a Shimadzu HPLC-MS 2020 with different analytic columns (ReproSil-Pur 300Å C4 3 μm, 2.1 × 100 mm; Hedera C18 3 μm, 2.1 × 100 mm) at a flow rate of 0.3 mL·min^-1^. The mobile phase was a combination of solvent A (0.1% FA in water) and solvent B (0.1% FA in acetonitrile). Temperature of the column heater was set on 40 ºC. Detector wavelength was set at 230 and 254 nm.

Preparative reverse-phase HPLC (RP-HPLC) was operated on Shimadzu LC-20 AR equipped with Shimadzu LC-20 AR Multi solvent Delivery System, Shimadzu CBM 20 A system controller and a Shimadzu SPD-20 A programmable wavelength detector. Preparative RP-HPLC was also operated on a Hanbon Sci. & Tech. semi-HPLC equipped with a Hanbon Sci. & Tech. NP7000 solvent delivery system and a Hanbon Sci. & Tech. NU3000 UV detector and a DM-A Dynamic mixer. The wavelengths of UV-detector were set to 230 nm and 254 nm. Preparative RP-HPLC was performed with different columns: Dubhe C18 (10 μm, 20 × 250 mm, 120 Å), Dubhe C18 (10 μm, 10 × 250 mm, 120 Å), Hedera C4 (10 μm, 20 × 250 mm, 100 Å) and Hedera C4 (10 μm, 10 × 250 mm, 100 Å), eluted with a mixed mobile phase of solvent A (0.05% TFA in water) and solvent B (0.05% TFA in acetonitrile) at a flow rate of 10 mL·min^-1^ (for small column) or 13 mL·min^-1^ (for big column).

High-resolution ESI mass spectra (HRMS (ESI)) were performed on a Thermo Q Exactive Orbitrap mass spectrometer coupled to a Dionex UHPLC.

Surface Plasmon Resonance (SPR)-based measurements were carried on Biacore 8K (GE Healthcare, Uppsala, Sweden) with Sensor Chip Protein A.

The Circular Dichroism (CD) spectra were recorded on a Jasco J-1500 spectrometer in a semi-micro quartz cell at 25 ºC (data interval 0.2 nm, path length 0.1 cm, scan rate 100 nm/min, response time 2 s).

The loading degree of the resins was determined by the UV absorbance at 301 nm of the Fmoc cleavage. The concentration of the protein was determined by UV absorbance at 280 nm. UV absorbance was detected on a UV-1100 spectrophotometer (MAPADA) with a semi-micro quartz cell.

Preparative size exclusion chromatography was performed on an Äkta Purifier HPLC with UV-detection at 214, 254 and 280 nm, with a column of GE Healthcare Sephadex^TM^ 75 (600 × 16 mm, 34 μm), eluted with PBS (1 ×) at a flow rate of 0.5 mL∙min^-1^.

Dialysis tubing Zellutrans V Serie (MWCO 10 kDa, flat width 25 mm) was obtained from Roth (Germany).

Abbreviations of amino acids and corresponding protecting groups: Pbf: 2,2,4,6,7-pentamethyl-2,3-dihydrobenzofuran-5-sulfonyl, Trt: triphenylmethyl, *t*-Bu: *t*-butyl, Boc: *t*-Butoxycarbonyl. Amino acids containing acid-labile protecting groups are marked in bold in the following schemes. Protecting groups are: R(Pbf), Q(Trt), E(*t*Bu), D(*t*Bu), N(Trt), W(Boc), K(Boc), C(Trt), S(*t*Bu), T(*t*Bu), Y(*t*Bu).

## 2. General Procedures

### 2.1 General Procedure A for manual solid-phase peptide synthesis (SPPS)

*Preloaded Fmoc-Leu-OH or Fmoc-Lys(Boc)-OH onto hydrazine resins*

Trityl chloride-ChemMatrix® resins (500.0 mg, 0.53 mmol, 1.0 equiv) were swollen in dry DCM for 3 min and then washed with DMF (3 × 5 mL). After that, the resins were treated with 10% hydrazine monohydrate in DMF for a certain time, then washed with DMF (3 × 5 mL), DCM (3 × 5 mL) and DMF (3 × 5 mL). The excess reactive sites were quenched by suspending the resins in a mixture of MeOH/DIPEA/DMF (2/1/7, v/v/v), gently shaken for 30 min at 25 ºC before washing with DMF (3 × 5 mL), DCM (3 × 5 mL) and DMF (3 × 5 mL).

Fmoc-based manual solid-phase peptide synthesis was conducted as following: the first amino acids were coupled by separately treating the resins with a solution of Fmoc-AA-OH (4.0 equiv, either Fmoc-Leu-OH or Fmoc-Lys(Boc)-OH), Oxyma (4.0 equiv) and DIC (5.0 equiv) in DMF, shaked for another 1 hour at 45 ºC before washing with DMF (3 × 5 mL), DCM (3 × 5 mL) and DMF (3 × 5 mL). After dried in high vacuum, trace resins were treated with piperidine/DMF (1:4, v/v) to release Fmoc. Then UV absorbance at 301 nm of the cleavage solution was measured to estimate the loading degree of the resins [1]. The surplus terminal amino groups were capped by treating the resins with a solution of acetic anhydride in pyridine (10%, volume ratio), gently shaken for 5 min at 25 ºC before washing with DMF (3 × 5 mL), DCM (3 × 5 mL) and DMF (3 × 5 mL).

*Fmoc-deprotection and* *Fmoc-AA-OH coupling*:

The N-terminal Fmoc group of the peptidyl resins were removed by suspending the peptidyl resins in a solution of piperidine/DMF (1/4, v/v, containing 0.1 M HOBt, 2 × 5 mL), gently shaken for 20 min (2 × 10 min) at 25 ºC, following washed with DMF (3 × 5 mL), DCM (3 × 5 mL) and DMF (3 × 5 mL). A solution of Fmoc-AA-OH (4.0 equiv, with final concentration of 0.2 M), Oxyma (4.0 equiv) and DIC (5.0 equiv) in DMF was mixed with the resins. The reaction suspension was gently shaken at 45 ºC for 30 min. After the reaction was completed, the peptidyl resins were washed with DMF (3 × 5 mL), DCM (3 × 5 mL) and DMF (3 × 5 mL).

*Conservative cleavage:*

After dried in high vacuum, the peptidyl resins were suspended in a diluted acidic solution of TFA in DCM (1%, v/v) and shaken for 1 min at 25 ºC. The resins were filtered and treated repeatedly for several times with the same method until the peptide was completely released. Combined the filtrate and the TFA was removed by washed with saturated sodium bicarbonate aqueous solution (NaHCO_3_). The organic phase was collected and dried over Na_2_SO_4_, then concentrated in vacuum to afford the crude N-terminal unprotected peptide.

*Global cleavage and purification:*

The full protected glycosylated polypeptide was dissolved in a mixture of cocktail B (TFA/phenol/water/*i*-Pr_3_SiH, 88/5/5/2, v/v/v/v) and agitated at 25 ºC for 1.5 hours to cleave the protecting groups completely. The resulted acidic solution was then concentrated by blowing with a gentle stream of N_2_. The remaining liquid was mixed with ice-cooled diethyl ether. The title peptide was precipitate, resulting suspension was centrifuged at 3500 rpm for 5 min. The supernatant was decanted, the white residue was washed with cold diethyl ether and centrifuged for another two times, then dried under reduced pressure. The crude peptide was dissolved in MeCN/H_2_O (with 0.05% TFA, volume ratio), further purified by preparative RP-HPLC.

### 2.2 General procedure B for protein expression and purification

The plasmids and pACYC-MAP were transformed into the *Escherichia coli* strain BL21(DE3), then plated on a selection plate containing ampicillin (100 μg·mL^-1^) and chloramphenicol (34 μg·mL^-1^), incubated 12 hours at 37 ºC. A single BL21 was inoculated in 5 mL Lysogeny Broth (LB) medium containing ampicillin (100 μg·mL^-1^) and chloramphenicol (34 μg·mL^-1^). The cell suspension was incubated at 37 ºC with shaking at 220 rpm for 12 hours. Subsequently, 2 mL of the cell suspension was diluted into 200 mL of LB medium containing ampicillin (100 μg·mL^-1^) and chloramphenicol (34 μg·mL^-1^), further shaken at 37 ºC until OD600 of the cell suspension reached 0.4-0.6. IPTG was added (final concentration 0.1 mM), following incubated for another 5 hours. Cells were harvested via centrifugation, and resuspended in phosphate buffer saline (100 mM, pH 8.0). The cells were disrupted by passing through a homogenizer, the lysate was centrifuged at 13000 rpm for 30 min at 4 ºC. The inclusion body was collected and dissolved in a buffer of GND•HCl (6 M), Na_2_HPO_4_ (200 mM), MeONH_2_·HCl (0.2 M), pH 4.0, then incubated at 37 ºC for 1 hour to unmask the N-terminal Cys residue. The mixture was centrifuged at 12000 rpm for 10 min at 4 ºC, supernatant containing RBD protein (fragment II) was collected and purified by preparative RP-HPLC.

### 2.3 General procedure C for enzymatic sialylation [2]

To a stock solution of tris (100 mM) and magnesium chloride (20 mM), oligosaccharide or glycopeptide (1.0 equiv, final Conc. 10 mg∙mL^-1^), Neu5Ac (4.0 equiv), CTP (8.0 equiv) were added, pH of the mixture was adjusted to 8.5 with 2.0 M NaOH solution, following added *PmST3* (final Conc. 0.30 mg∙mL^-1^) and *NmCSS* (final Conc. 0.30 mg∙mL^-1^). Then the reaction mixture was incubated at 37 ºC for 8 hours. The reaction was cooled to room temperature, equal volume ice-cooled EtOH was added, resulting suspension was centrifuged at 3500 rpm for 5 min. The supernatant was collected and the EtOH was removed under reduced pressure, the residue was further purified by preparative RP-HPLC.

### 2.4 General Procedure D for conversion of glycopeptide hydrazide to glycopeptide thioester and 1^st^ NCL in one pot [3]

Glycopeptide hydrazide (1.0 equiv, final Conc. 12.5 mM) was dissolved in a solvent of GND•HCl (6.0 M), Na_2_HPO_4_ (0.2 M), pH 3.0. The mixture was degassed with N_2_ bubbling and cooled to -15 ºC, then kept it stirring for 5 min. Subsequently, an freshly prepared ice-cooled stock solution of 0.4 M NaNO_2_ (6.0 equiv) was added slowly by micro-syringe. The reaction mixture was kept stirring at -15 ºC for another 15 min under N_2_ atmosphere. A solution of 200 mM MPAA (60 equiv) in the solvent of GND•HCl (6.0 M), Na_2_HPO_4_ (0.2 M), pH 7.0 was added. Subsequently, *N*-terminal Cys glycopeptide (1.1 equiv) were added. pH of the reaction mixture was adjusted to 6.8-7.0 with 2.0 M NaOH solution. The reaction was warmed to room temperature, degassed and further stirred for 2 more hours. Upon completion, equal volume solution of TCEP (150 mM) in GND•HCl (6.0 M) and Na_2_HPO_4_ (0.2 M), pH 6.5 was added. After stirring for 30 min, the reaction mixture was diluted with MeCN/H_2_O and further purified by preparative RP-HPLC.

### 2.5 General Procedure E for conversion of glycopeptide hydrazide to glycopeptide thioester and 2^nd^ NCL in one pot [3]

Glycopeptide hydrazide (1.02 equiv, final Conc. 5.7 mM) was dissolved in a solvent of GND•HCl (6.0 M), Na_2_HPO_4_ (0.2 M), pH 3.0. The mixture was degassed with N_2_ bubbling and cooled to -15 ºC, then kept it stirring for 5 min. Subsequently, a freshly prepared ice-cooled stock solution of 0.4 M NaNO_2_ (6.0 equiv) was added slowly by micro-syringe. The reaction mixture was kept stirring at -15 ºC for another 15 min under N_2_ atmosphere. A solution of 200 mM MPAA (60 equiv) in the solvent of GND•HCl (6.0 M), Na_2_HPO_4_ (0.2 M), pH 7.0 was added. Subsequently, *N*-terminal Cys containing recombinant fragment (1.0 equiv) were added. pH of the reaction mixture was adjusted to 6.5-6.8 with 2.0 M NaOH solution. The reaction was warmed to room temperature, degassed and further stirred for 3 more hours. Upon completion, equal volume solution of TCEP (150 mM) in GND•HCl (6.0 M) and Na_2_HPO_4_ (0.2 M), pH 6.5 was added. After stirring for 30 min, the reaction mixture was diluted with MeCN/H_2_O and further purified by preparative RP-HPLC.

### 2.6 General Procedure F for protein refolding and SEC-HPLC purification procedure

Denatured protein refolding was carried out according to the procedure developed in our lab with some modifications [4]. Specifically, protein refolding was conducted by stepwise dialysis according to the following procedure. Lyophilized glycoprotein was dissolved in a denaturing stock buffer of DTT (10 mM), GND•HCl (6.0 M) and Tris·HCl (50 mM), buffered at pH 8.0, further incubated at 37 ºC for 30 min in an anaerobic atmosphere. The UV absorbance at 280 nm of the solution was measured in a semi-micro quartz cell to estimate the concentration of the protein. The solution of glycoprotein was diluted with a buffer of GND•HCl (6.0 M), Tris·HCl (50 mM), pH 8.0 to final concentration of the glycoprotein 50 μg·mL^-1^. The solution was transferred to a dialysis tubing (10 kDa MWCO, Pierce, 1.5-fold volume to the solution), following dialysed against 100-fold volume (base on the volume of the tube) of GND•HCl (6.0 M) and Tris·HCl (50 mM), pH 8.0 for 12 hours at 4 ºC to remove DTT. Subsequently, the dialysis was carried out against the following buffer systems to initiate the refolding: A) Tris·HCl (50 mM), GND•HCl (2 M), Arg (400 mM), GSH (3 mM), GSSG (0.9 mM), pH 8.0, 4 ºC, 36 hours. B) Tris·HCl (25 mM), GND•HCl (1 M), Arg (200 mM), GSH (1.5 mM), GSSG (0.45 mM), pH 8.0, 4 ºC, 24 hours. C) Tris·HCl (25 mM), NaCl (250 mM), Arg (100 mM), GSH (1.5 mM), GSSG (0.45 mM), pH 8.0, 4 ºC, 24 hours. D) Tris·HCl (25 mM), NaCl (250 mM), pH 8.0, 4 ºC, 12 hours.

Upon completion, the solution in dialysis tubing was transferred to a centrifugal tube and the dialysis tubing was washed with PBS (1 ×). The solution was combined, centrifuged to remove the aggregation. The supernatant was concentrated by ultrafiltering (Centrifugal Filter, 10 kDa MWCO, regenerated cellulose, purchased from Merck Millipore Ltd.) until the volume of the solution is less than 2.0 mL. The residue was further purified by preparative size exclusion chromatography (SEC-HPLC). SEC-HPLC was performed on an Äkta Purifier HPLC with UV-detection at 214, 254 and 280 nm, with a column of GE Healthcare SephadexTM 75 (600 × 16 mm, 34 μm), eluted with PBS (1 ×) at a flow rate of 0.5 mL∙min^-1^. UV absorbance at 280 nm of the refolded protein in PBS was detected, then further estimated the concentration of the final protein solution and calculated the folding yield.

### 2.7 General Procedure G for surface plasmon resonance (SPR) analysis

Surface plasmon resonance (SPR)-based assays were conducted according to previous report [5], to explore the real-time binding ability between RBDs (commercially available HEK393 RBD and synthetic RBDs) and antibodies or human ACE2 on Biacore 8K. Antibodies and ACE2 were immobilized on Sensor Chip Protein A (Cytiva) at a flow rate of 10 μL·min^-1^ for 60 s (to ~50-100 RU). The RBD was diluted to 2-fold series and flowed over the chip at a flow rate of 30 μL·min^-1^ for 120 s for kinetic analysis (an empty channel was set in each group as control). RBDs bound on the chip were dissociated by HBS-ET running buffer (1 ×, Cytiva) at a flow rate of 30 μL·min^-1^ for 300 s. Sensor Chip Protein A was regenerated by glycine buffer (10 mM, pH 1.5) at a flow rate of 30 μL·min^-1^ for 60 s. The results were analyzed on Biacore Insight Evaluation software to afford association rate constants (*K_a_*), dissociation rate constants (*K_d_*) and affinity constants (*K_D_*).

## 3. Synthesis, purification and characterization of RBD 1

### 3.1 Enzymatically sialylated of nonasaccharide and nonasaccharide modified peptides[2]

**Scheme S1:** Synthesis of glycosyl amine **7** and **8**

Nonasaccharide azide **S1** (33.2 mg, 20.4 μmol, 1.0 equiv, synthesized according previous work [4]) was added to a 1mL bottle containing dry MeOH (0.6 mL), DIPEA (35 μL, 0.2 mmol, 10 equiv) and 1,3-dimercaptopropane (120 μL, 1.2 mmol, 60 equiv) were following added. The bottle was sealed and stirred vigorously at 25 ℃ for 2 hours. The evaporable solvent was removed under reduced pressure. The residue was suppended in ice-cold diethyl ether (2 mL), then centrifuged. The white precipitate was dried under vacuum to afford crude title product **7** (31.1 mg) as a white powder, which was used directly without further purification.

According to **General Procedure C**, sialylated undecasaccharide azide **S2** was synthesized with **S1** (47.0 mg, 28.2 μmol, 1.0 equiv). After the reaction completed, equal volum of ice-cold ethanol was added, the suspended enzyme was removed by centrifuging. The supernatant was collected and concentrated under vacuum. The residue was purified by Bio-Gel P4 (Bio-Rad), eluted with water. A white powder **S2** (47.1 mg, 74% isolated yield) was obtained after freeze-drying. HRMS (ESI+): Calculated Mass for C84H137N_9_O61 [M+2Na]^2+^: 1146.8845, found: 1146.8848.

Sialylated undecasaccharide azide **S2** (26.5 mg, 12.8 μmol, 1.0 equiv) was added to a 1 mL bottle containing dry MeOH (0.64 mL), DIPEA (23 μL, 0.13 mmol, 10 equiv) and 1,3-dimercaptopropane (77 μL, 0.77 mmol, 60 equiv) were following added. The bottle was sealed and stirred vigorously at 25 ℃ for 2 hours. The evaporable solvent was removed under reduced pressure. The residue was suppended in ice-cold diethyl ether (2 mL), then centrifuged. The white precipitate was dried under vacuum to afford crude title product **8** (24.2 mg) as a white powder, which was used directly without further purification.

**Scheme S2:** Synthesis of sialylated glycopeptides

Glycopeptidyl hydrazide **10** was synthesized by enzyme catalytic reaction according to the protocol described in **General Procedure C** with nonasaccharide modified glycopeptidyl hydrazide **9** (12.1 mg, 3.34 μmol, 1.0 equiv, synthesized according to our previous work [4]). The resulting reaction mixture was purified by preparative RP-HPLC (Dubhe C18 column, 10 μm, 20 × 250 mm, linear gradient 18%-30% of solvent B over 30 min). The undecasaccharide modified glycopeptidyl hydrazide **10** was obtained as a white lyophilized powder (11.7 mg, 83% isolated yield).


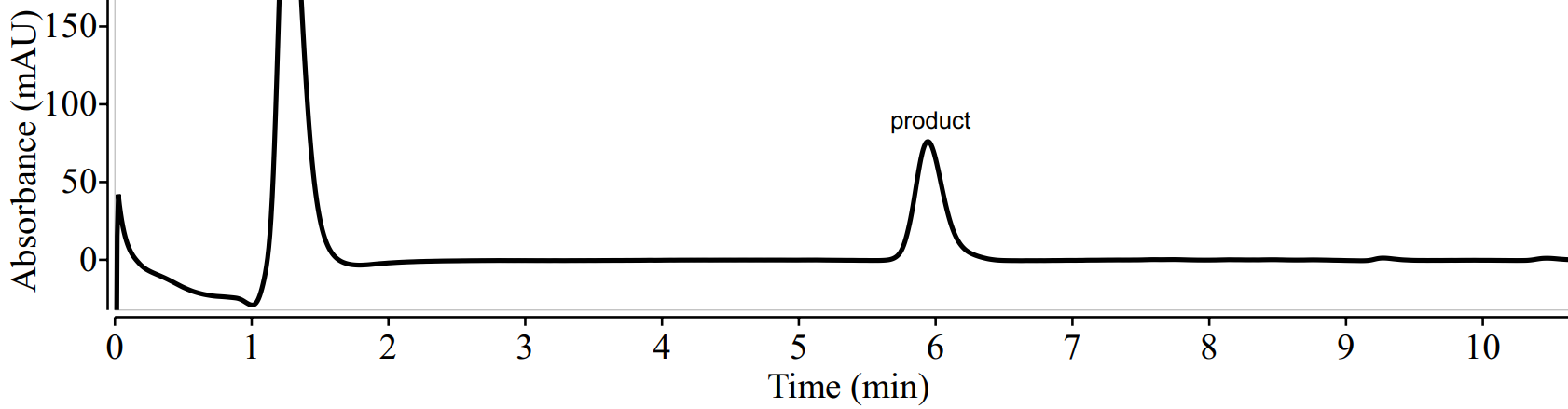


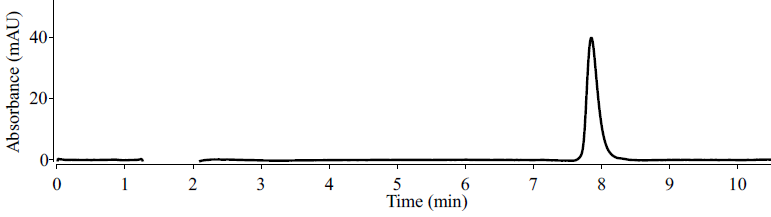


**Figure S 1.** Analytical HPLC profile of crude (top) and purified (bottom) **10** (*t*_R_ = 7.9 min, Hedera C18 column, 2.1 × 100 mm, 3 μm, linear gradient 10%-40% of solvent B over 10 min).


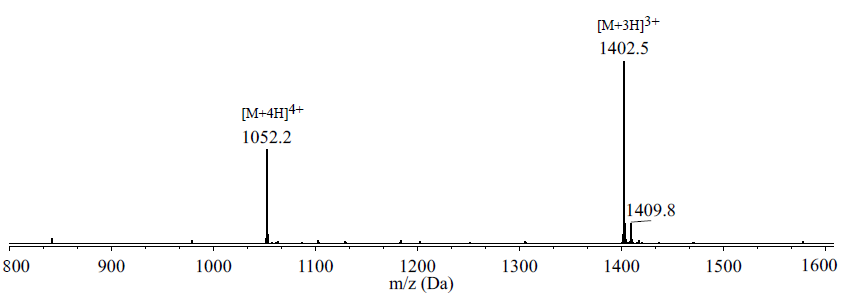


**Figure S 2.** Low-resolution MS (ESI) spectrum of purified **10**, Calculated Mass for C_172_H_284_N_34_O_86_ [M+3H]^3+^: 1402.4; [M+4H]^4+^: 1052.1; Mass Found (ESI+) [M+3H]^3+^: 1402.5; [M+4H]^4+^: 1052.2.

Glycopeptidyl hydrazide **12** was synthesized by enzyme catalytic reaction according to the protocol described in **General Procedure C** with nonasaccharide modified glycopeptidyl hydrazide **11** (10.8 mg, 2.32 μmol, 1.0 equiv, synthesized according to our previous work [4]). The resulting reaction mixture was purified by preparative RP-HPLC (Dubhe C18 column, 10 μm, 20 × 250 mm, linear gradient 20%-25% of solvent B over 30 min). The undecasaccharide modified glycopeptidyl hydrazide **12** was obtained as a white lyophilized powder (9.24 mg, 76% isolated yield).


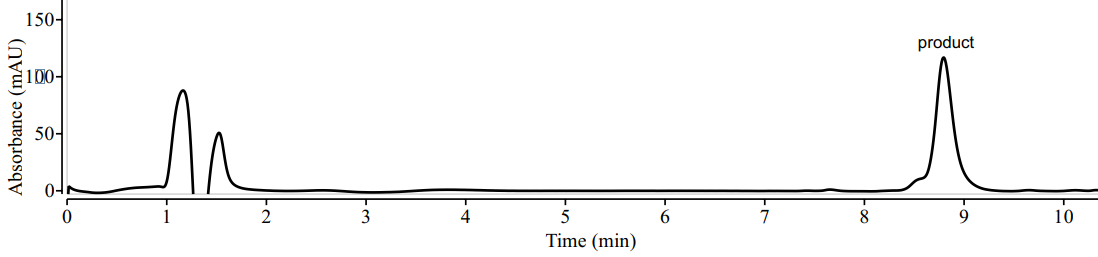


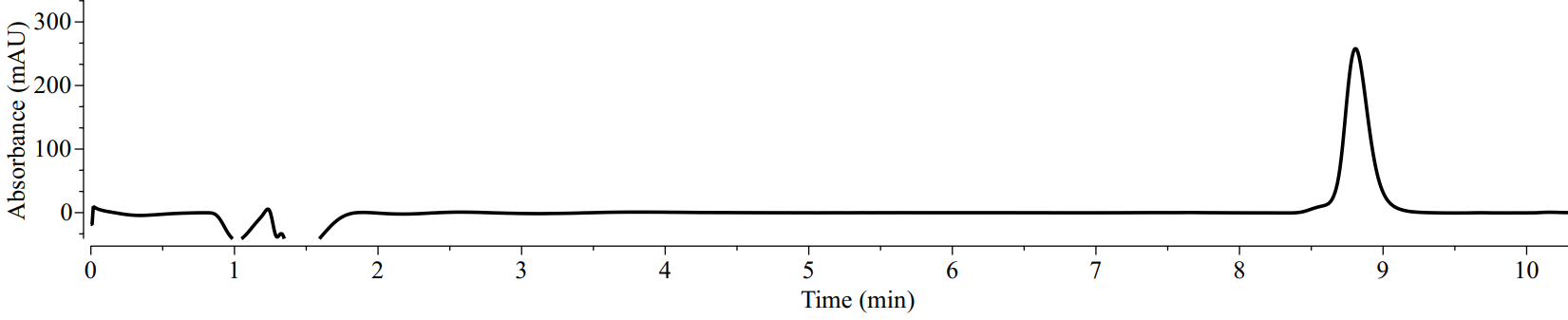


**Figure S 3.** Analytical HPLC profile of crude (top) and purified (bottom) **12** (*t*_R_ = 8.8 min, Hedera C18 column, 2.1 × 100 mm, 3 μm, linear gradient 5%-50% of solvent B over 10 min).


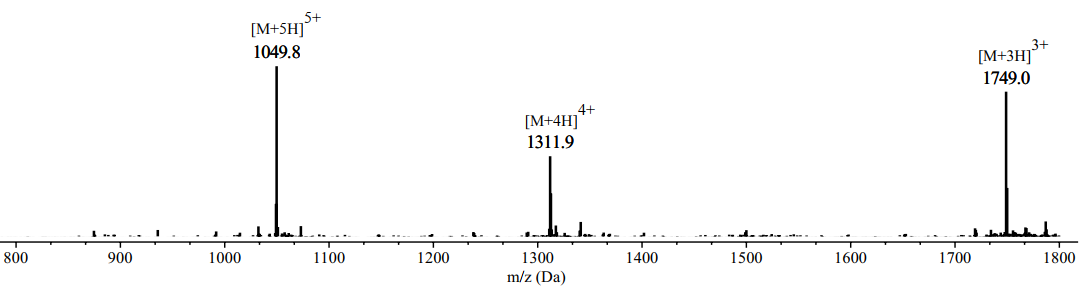


**Figure S 4.** Low-resolution MS (ESI) spectrum of purified **12**, Calculated Mass for C_221_H_343_N_47_O_95_S2 [M+3H]^3+^: 1748.5; [M+4H]^4+^: 1311.6; [M+5H]^5+^: 1049.5; Mass Found (ESI+) [M+3H]^3+^: 1749.0; [M+4H]^4+^: 1312.1; [M+5H]^5+^: 1049.8.

NCL between glycopeptidyl hydrazide **10** (0.87 mg, 0.20 μmol, 1.1 equiv) and *N*-terminal Cys glycopeptidyl hydrazide **12** (1.0 mg, 0.19 μmol, 1.0 equiv) was conducted acccording to **General Procedure D** through two-step reaction in one-pot to assemble glycopeptidyl hydrazide **13**. The resulting ligation mixture was purified by preparative RP-HPLC (Hedera C4 column, 10 μm, 10 × 250 mm, linear gradient 20%-50% of solvent B over 30 min). The glycopeptidyl hydrazide **13** was obtained as a white lyophilized powder (1.12 mg, 63% isolated yield).


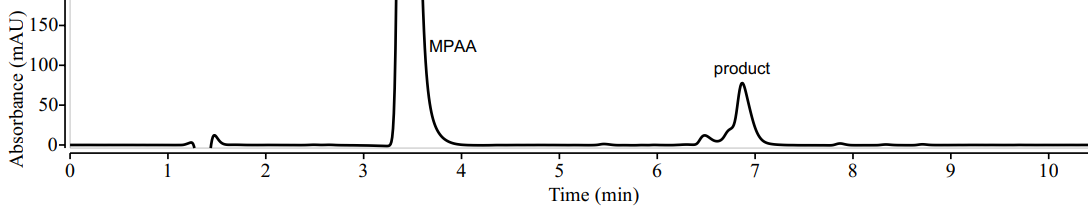


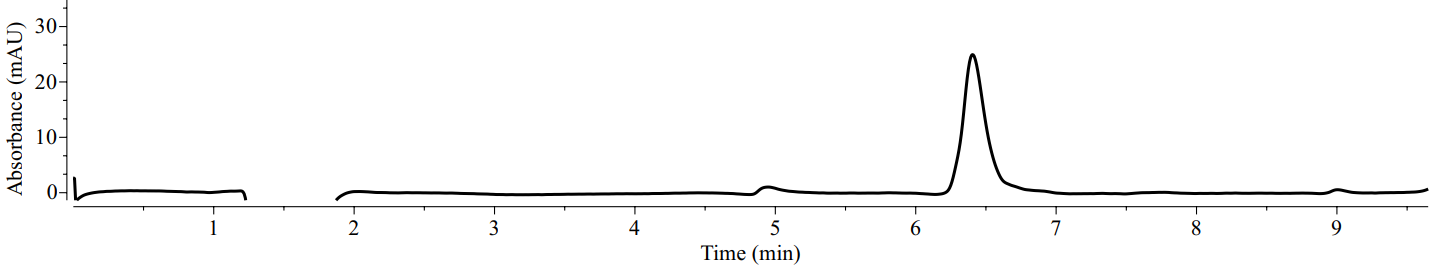


**Figure S5.** Analytical HPLC profile of crude (top) and purified (bottom) **13** (*t*_R_ = 6.4 min, Hedera C18 column, 2.1 × 100 mm, 3 μm, linear gradient 20%-60% of solvent B over 10 min).


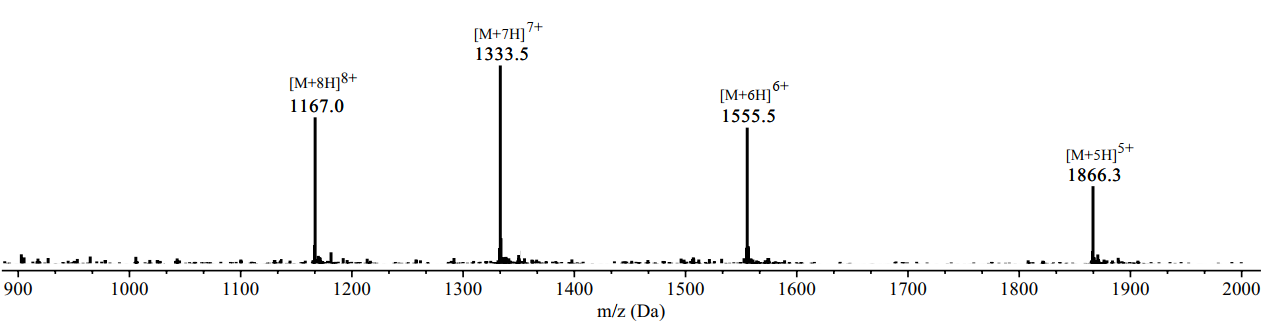


**Figure S6.** Low-resolution MS (ESI) spectrum of purified **13**, Calculated Mass for C_389_H_615_N_79_O_181_S [M+5H]^5+^: 1866.3; [M+6H]^6+^: 1555.4; [M+7H]^7+^: 1333.4; [M+8H]^8+^: 1166.8; Mass Found (ESI+) [M+5H]^5+^: 1866.3; [M+6H]^6+^: 1555.5; [M+7H]^7+^: 1333.5; [M+8H]^8+^: 1167.0.

NCL pilot test between glycopeptidyl hydrazide **13** (0.57 mg, 0.11 μmol, 1.0 equiv) and free Cysteine (26.7 μg, 0.22 μmol, 2.0 equiv) was conducted acccording to **General Procedure D**. After stirring for two hours, LCMS was applied to monitor the reaction. The results showed no ligation product, but only hydrolysis byproduct (**14**) of **13**.

Additionally, glycopeptidyl hydrazide **13** was also transferred to the corresponding thioester in the presence of acetylacetone. The LCMS showed the same results, no thioester product but only hydrolysis byproduct **14**.


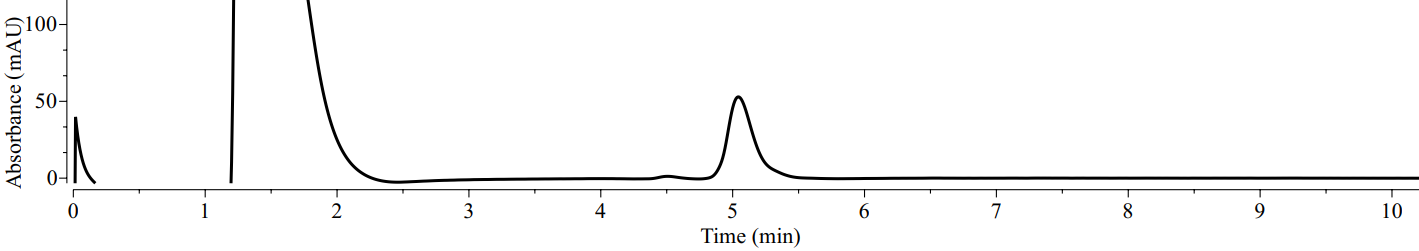


**Figure S7.** Analytical HPLC profile of crude **14** (*t*_R_ = 5.1 min, Hedera C4 column, 2.1 × 100 mm, 3 μm, linear gradient 20%-60% of solvent B over 10 min).


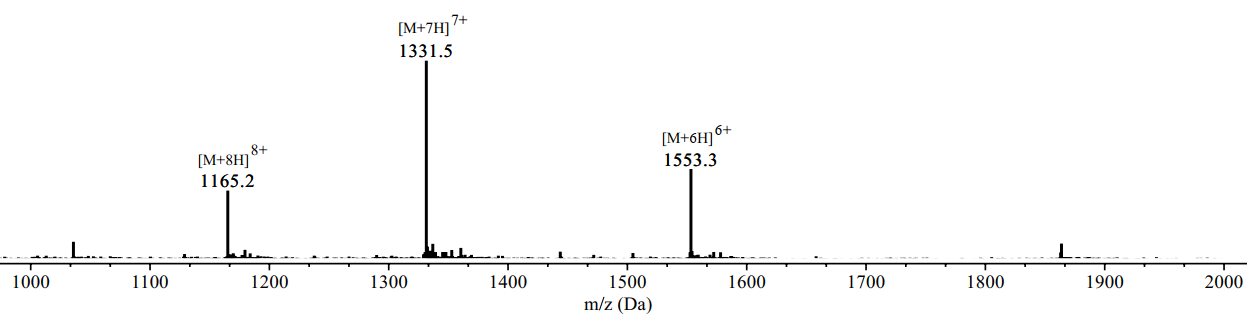


**Figure S8.** Low-resolution MS (ESI) spectrum of purified **13**, Calculated Mass for C_389_H_613_N_77_O_182_S [M+6H]^6+^: 1553.1; [M+7H]^7+^: 1331.4; [M+8H]^8+^: 1165.1; Mass Found (ESI+) [M+6H]^6+^: 1553.3; [M+7H]^7+^: 1331.5; [M+8H]^8+^: 1165.2.

**Scheme S3:** Alternative routine of sialylated glycopeptide synthesis

Amino acids containing acid-labile protecting groups are marked in bold in the schemes. Pseudoproline dipeptides are shown in red (underlined). Protecting groups are: R(Pbf), Q(Trt), E(*t*Bu), D(*t*Bu), N(Trt), W(Boc), K(Boc), C(Trt), S(*t*Bu), T(*t*Bu), Y(*t*Bu).

Fmoc-Lys(Boc)-OH pre-loaded resins (1.10 g, 0.50 mmol·g^-1^) were used to synthesize resin-bound peptide **S3** according to generally manual SPPS protocols described in **General Procedure A**. Allyl (All) protected Fmoc-Asp(All)-OH and pseudoproline dipeptide Fmoc-Tyr-Ser(ψ^Me,Me^Pro)-OH, Fmoc-Ala-Thr(ψ^Me,Me^Pro)-OH were introduced in the corresponding sites [6].

Resin-bound peptide was subjected to conservative cleavage with diluted TFA solution (1% in DCM, v/v) according to **General Procedure A** to give crude protected peptidyl hydrazide (4.23 g). The crude product was used in the next step without further purification.

The crude peptidyl hydrazide was dissolved in a mixture of THF (20 mL) and saturated NaHCO_3_ aqueous solution (10 mL), Boc_2_O (1.26 mL, 5.5 mmol, 10.0 equiv, based on initial loading value) were following added. The mixture was further stirred vigorously at 25 ºC for 6 hours. After the reaction completed, the reaction mixture was diluted with AcOEt (70 mL), the organic phase was seperated and dried over Na_2_SO_4_. After filtrating, the solution was concentrated under reduced pressure to give crude protected peptide. The residue was further purified by silica gel column chromatography (DMC/MeOH, 40/1-30/1, v/v, with trace trimethylamine) to afford allyl protected peptide **S4** (2.55 g, 57% yield, based on initial loading value) as a white solid.


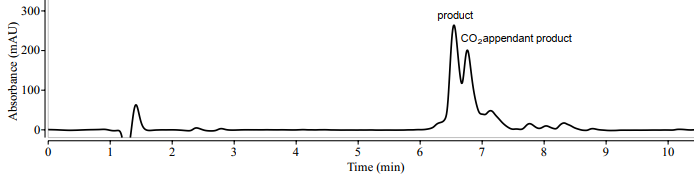


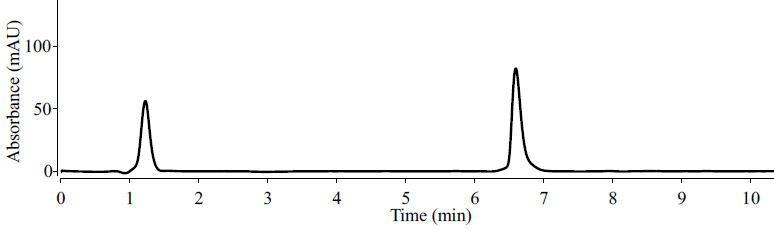


**Figure S9.** Analytical HPLC profile of crude (top) and purified (bottom) **S4** after globally deprotected with Cocktail B (*t*_R_ = 6.6 min, ReproSil-Pur 300Å C4 column, 2.1 × 100 mm, 3 μm, linear gradient 15%-50% of solvent B over 10 min).

**
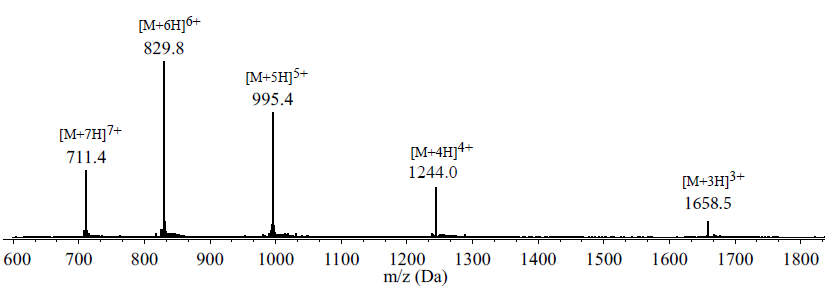
**

**Figure S10.** Low-resolution MS (ESI) spectrum of purified **S4** after globally deprotected with Cocktail B, Calculated Mass for C_227_H_332_N_60_O_63_S_2_ [M+3H]^3+^: 1658.9; [M+4H]^4+^: 1244.4; [M+5H]^5+^: 995.7; [M+6H]^6+^: 829.9; [M+7H]^7+^: 711.5; Mass Found (ESI+) [M+3H]^3+^: 1658.5; [M+4H]^4+^: 1244.0; [M+5H]^5+^: 995.4; [M+6H]^6+^: 829.8; [M+7H]^7+^: 711.4.

To a solution of allyl protected peptide **S4** (2.12 g, 0.26 mmol, 1.0 equiv) in DCM (10 mL), Pd(PPh_3_)_4_ (150 mg, 0.13 mmol, 0.5 equiv) and PhSiH_3_ (321 μL, 2.60 mmol, 10.0 equiv) were added. The mixture was kept stirring at 25 ºC for 1 hour. During the progress, the reaction mixture turned from light yellow to dark brown. Upon completion, the reaction mixture was concentrated under reduced pressure, then further purified by silica gel column chromatography (DMC/MeOH, 20/1-10/1, v/v) to afford protected peptidyl acid **15** (1.68 g, 80% isolated yield) as a gray solid.


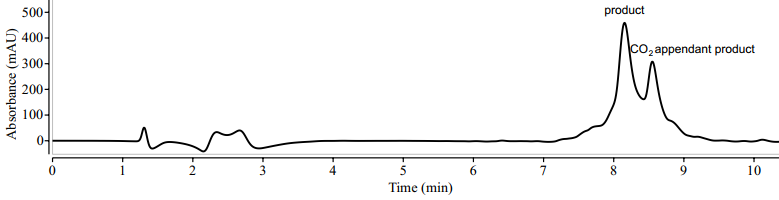


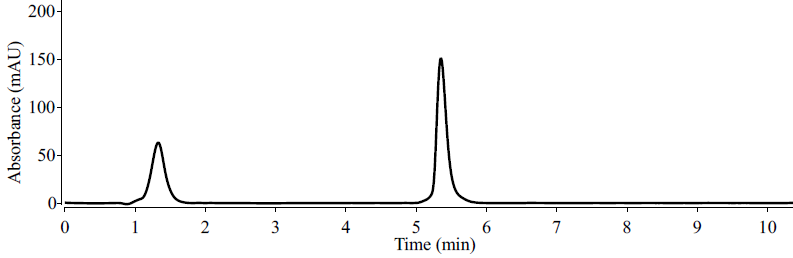


**Figure S11.** Analytical HPLC profile of crude (top) and purified (bottom) **15** after globally deprotected with Cocktail B (*t*_R_ = 5.5 min, ReproSil-Pur 300Å C4 column, 2.1 × 100 mm, 3 μm, linear gradient 20%-50% of solvent B over 10 min).

**
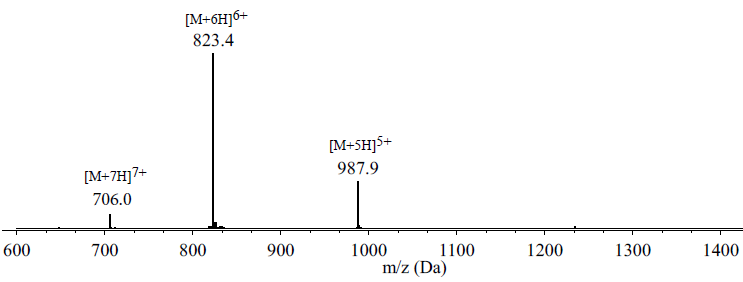
**

**Figure S12.** Low-resolution MS (ESI) spectrum of purified **15** after globally deprotected with Cocktail B, Calculated Mass for C_227_H_332_N_60_O_63_S_2_ [M+5H]^5+^: 987.7; [M+6H]^6+^: 823.3; [M+7H]^7+^: 705.8; Mass Found (ESI+) [M+5H]^5+^: 987.9; [M+6H]^6+^: 823.4; [M+7H]^7+^: 706.0.

Protected peptidyl acid **15** (90.0 mg, 11.1 μmol, 1.0 equiv) was glycosylated with nonasaccharide amine (23.7 mg, 14.4 μmol, 1.3 equiv) in the presence of HATU (8.44 mg, 22.2 μmol, 2.0 equiv), HOBt (3.15 mg, 23.3 μmol, 2.1 equiv) and DIPEA (7.7 μL, 44.4 μmol, 4.0 equiv) in anhydrous DMSO (150 μL) [7,8]. The reaction was kept stirring vigorously at 25 ºC for 3 hours. After the reaction completed, the protecting groups were globally cleaved by addition of Cocktail B according to **General Procedure A**. The crude product was purified by preparative RP-HPLC (Dubhe C18 column, 10 μm, 20 × 250 mm, linear gradient 20%-35% of solvent B over 30 min). Glycopeptide **16** was obtained as a white lyophilized powder (35.7 mg, 49% isolated yield).

**
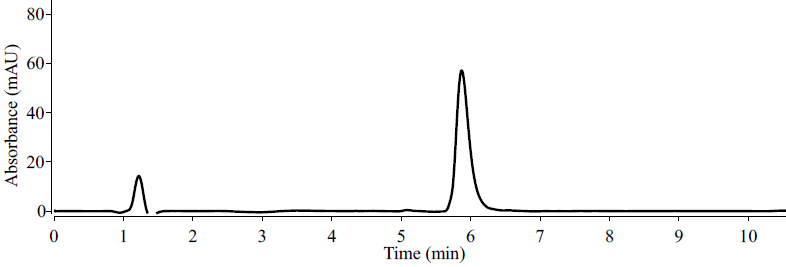
**

**Figure S13.** Analytical HPLC profile of purified **16** (*t*_R_ = 5.9 min, Hedera C18 column, 2.1 × 100 mm, 3 μm, linear gradient 20%-50% of solvent B over 30 min).

**
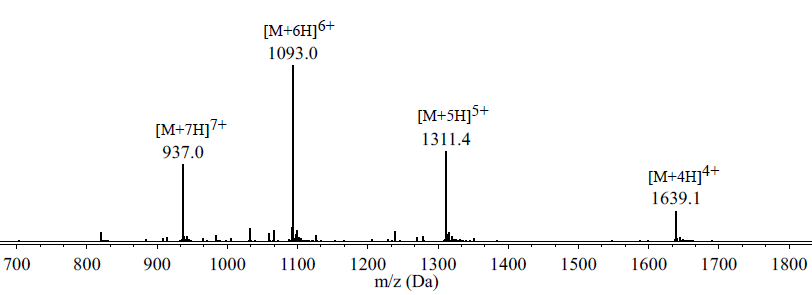
**

**Figure S14.** Low-resolution MS (ESI) spectrum of purified **16**, Calculated Mass for C_286_H_431_N_65_O_107_S_2_ [M+4H]^4+^: 1639.0 [M+5H]^5+^: 1311.4; [M+6H]^6+^:1093.0; [M+7H]^7+^: 937.0; Mass Found (ESI+) [M+4H]^4+^: 1639.1 [M+5H]^5+^: 1311.4; [M+6H]^6+^:1093.0; [M+7H]^7+^: 937.0.

Compound **16** (2.52 mg, 0.38 μmol, 1.0 equiv) was sialylated according to **General Procedure A**. nonasaccharide modified **16** showed poor solubility, amount white precipitate aggregate during the progress even thought the reaction conducted in low concentration (1.0 mg·mL^-1^). LCMS was applied to monitor the reaction, the results showed no title product, and comfirmed the precipitate was compound **16**.

### 3.2 Sialylated glycopeptide and RBD 1 synthesis and characterization with alternative method

**Scheme S4:** Alternative routine of sialylated glycopeptide and **RBD 1** synthesis

Amino acids containing acid-labile protecting groups are marked in bold in the schemes. Protecting groups are: C(Trt), E(*t*Bu), R(Pbf), S(*t*Bu), Y(*t*Bu), W(Boc), N(Trt), D(*t*Bu), T(*t*Bu), K(Boc).

Full nucleotides sequence (forward, the cleavage sites are marked in bold) and amino acids

sequence of fragment **19** (C379-K5537-GGG-T_pep_) were listed as follow.

**CATATG**TGCTACGGCGTGAGCCCCACCAAGCTGAACGACCTGTGCTTCACCAACGTGTACGCCGACAGCTTCGTGATCAGGGGCGACGAGGTGAGGCAGATCGCCCCCGGCCAGACCGGCAAGATCGCCGACTACAACTACAAGCTGCCCGACGACTTCACCGGCTGCGTGATCGCCTGGAACAGCAACAACCTGGACAGCAAGGTGGGCGGCAACTACAACTACCTGTACAGGCTGTTCAGGAAGAGCAACCTGAAGCCCTTCGAGAGGGACATCAGCACCGAGATCTACCAGGCCGGCAGCACCCCCTGCAACGGCGTGGAGGGCTTCAACTGCTACTTCCCCCTGCAGAGCTACGGCTTCCAGCCCACCAACGGCGTGGGCTACCAGCCCTACAGGGTGGTGGTGCTGAGCTTCGAGCTGCTGCACGCCCCCGCCACCGTGTGCGGCCCCAAGAAGAGCACCAACCTGGTGAAGAACAAGGGCGGCGGCCAGTACATCAAGGCCAACAGCAAGTTCATCGGCATCACCGAG**CTCGAG**

**Figure S15.** Full nucleotides (top) and amino acids sequence (bottom) of recombiant fragment **19** (C379-K5537-GGG-T_pep_)

The plasmid pET21a-rbd-tt was employed to recombine RBD fragment **19** (C379-K5537-GGG-T_pep_) according to the protocol described in **General Procedure B**. Supernatant containing fragment **B** was purified by preparative RP-HPLC (Hedera C4 column, 10 μm, 20 × 250 mm, linear gradient 25%-45% of solvent B over 30 min). Fragment **19** was obtained as a white lyophilized powder (23 mg·L^-1^, isolated yield).


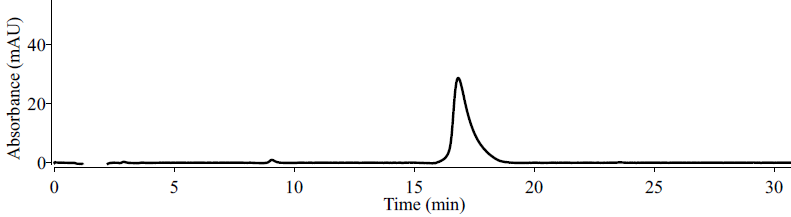


**Figure S16.** Analytical HPLC profile of purified fragment **19** (*t*_R_ = 16.9 min, ReproSil-Pur 300Å C4 column, 2.1 × 100 mm, 3 μm, linear gradient 25%-40% of solvent B over 30 min).


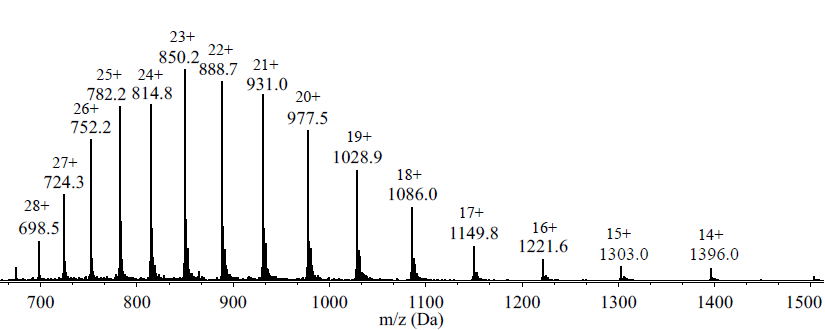


**Figure S17.** Low-resolution MS (ESI) spectrum of purified fragment **19**, Calculated Mass for Calculated Mass for C_881_H_1337_N_231_O_261_S_6_: 19532.82 Da (molecular weight), [M+14H]^14+^: 1396.2; [M+15H]^15+^: 1303.2; [M+16H]^16+^: 1221.8; [M+17H]^17+^: 1150.0; [M+18H]^18+^: 1086.2; [M+19H]^19+^: 1029.1; [M+20H]^20+^: 977.7; [M+21H]^21+^: 931.1; [M+22H]^22+^: 888.9; [M+23H]^23+^: 850.3; [M+24H]^24+^: 814.9; [M+25H]^25+^: 782.3; [M+26H]^26+^: 752.3; [M+27H]^27+^: 724.4; [M+28H]^28+^: 698.6; Mass Found (ESI+) [M+14H]^14+^: 1396.0; [M+15H]^15+^: 1303.0; [M+16H]^16+^: 1221.6; [M+17H]^17+^: 1149.8; [M+18H]^18+^: 1086.0; [M+19H]^19+^: 1028.9; [M+20H]^20+^: 977.5; [M+21H]^21+^: 931.0; [M+22H]^22+^: 888.7; [M+23H]^23+^: 850.2; [M+24H]^24+^: 814.8; [M+25H]^25+^: 782.2; [M+26H]^26+^: 752.2; [M+27H]^27+^: 724.3; [M+28H]^28+^: 698.5.


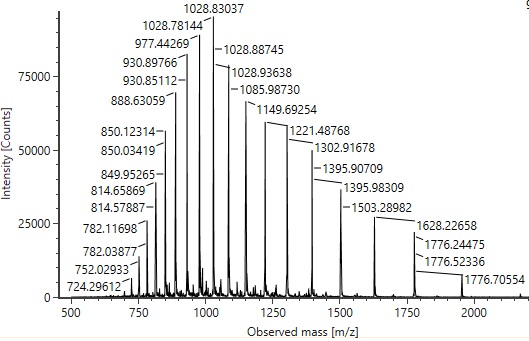

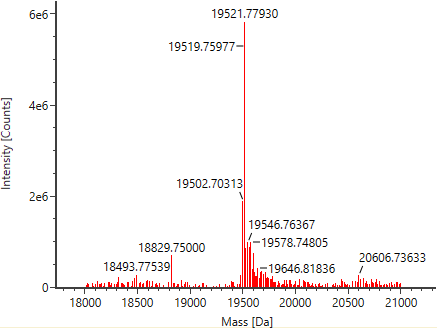


**Figure S18.** High-resolution MS (ESI) spectrum of purified fragment **19**, Calculated Mass for C_881_H_1337_N_231_O_261_S_6_, [M+H]^+^: 19521.68399; Mass Found (ESI+) [M+H]^+^: 19521.77930 (deconvoluted).

Protected peptidyl acid **15** (85.0 mg, 10.5 μmol, 1.0 equiv) was glycosylated with undecasaccharide amine (30.3 mg, 13.7 μmol, 1.3 equiv) in the presence of HATU (40.0 mg, 105 μmol, 10 equiv), HOBt (17.0 mg, 126 μmol, 12 equiv) and DIPEA (35.6 μL, 0.21 mmol, 20 equiv) in anhydrous DMSO (200 μL). The reaction was kept stirring vigorously at 25 ºC for 3 hours. After the reaction completed, the protecting groups were globally cleaved by addition of Cocktail B according to **General Procedure A**. The crude product was dissolved in a buffer of GND•HCl (6.0 M), Na_2_HPO_4_ (0.2 M), pH 8.0. After stirring for 10 min, lactone completely hydrolyzed, the solution was further purified by preparative RP-HPLC (Hedera C4 column, 10 μm, 10 × 250 mm, linear gradient 20%-35% of solvent B over 30 min). Glycopeptidyl hydrazide **17** was obtained as a white lyophilized powder (31.5 mg, 42% isolated yield).


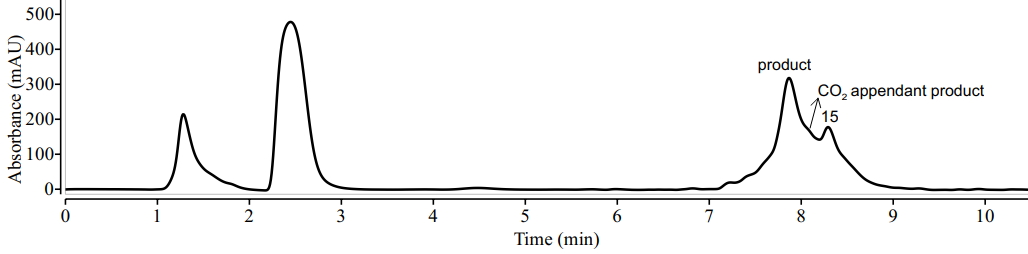


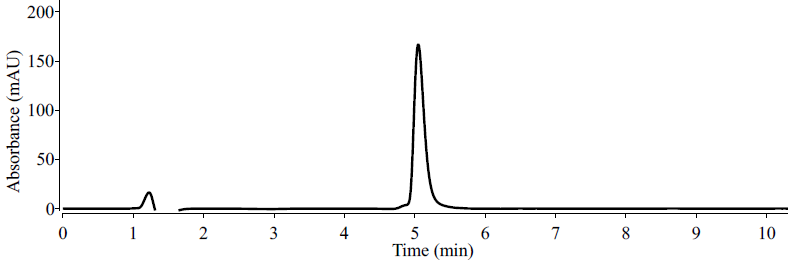


**Figure S19.** Analytical HPLC profile of crude (top) and purified (bottom) **17** (*t*_R_ = 5.1 min, ReproSil-Pur 300Å C4 column, 2.1 × 100 mm, 3 μm, linear gradient 20%-60% of solvent B over 10 min).


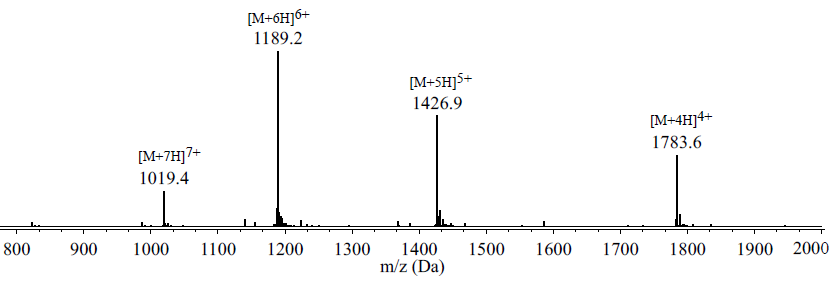


**Figure S20.** Low-resolution MS (ESI) spectrum of purified **17**, Calculated Mass for C_308_H_465_N_67_O_123_S_2_ [M+4H]^4+^: 1784.2; [M+5H]^5+^: 1427.3; [M+6H]^6+^: 1189.5; [M+7H]^7+^: 1020.0; Mass Found (ESI+) [M+4H]^4+^: 1783.6; [M+5H]^5+^: 1426.9; [M+6H]^6+^: 1189.2; [M+7H]^7+^: 1019.4.

NCL between glycopeptidyl hydrazide **10** (2.42 mg, 0.58 μmol, 1.1 equiv) and *N*-terminal Cys glycopeptidyl hydrazide **17** (3.76 mg, 0.53 μmol, 1.0 equiv) was conducted acccording to **General Procedure D** through two-step reaction in one-pot to assemble glycopeptidyl hydrazide **18**. The resulting ligation mixture was purified by preparative RP-HPLC (Hedera C4 column, 10 μm, 10 × 250 mm, linear gradient 20%-35% of solvent B over 30 min). The glycopeptidyl hydrazide **18** was obtained as a white lyophilized powder (3.66 mg, 61% isolated yield).


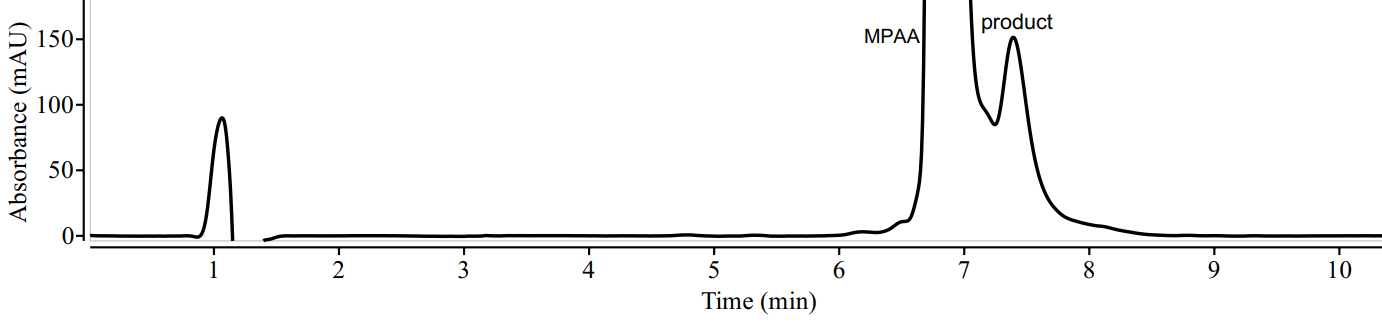


**
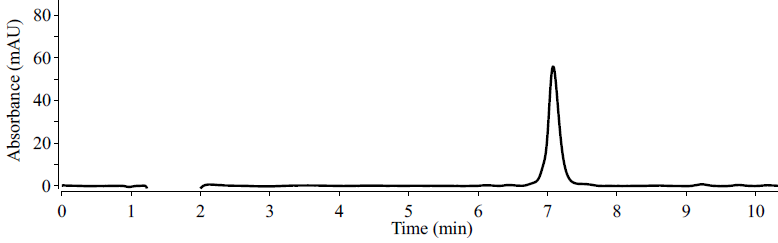
**

**Figure S21.** Analytical HPLC profile of crude (top) and purified (bottom) **18** (*t*_R_ = 7.1 min, ReproSil-Pur 300Å C4 column, 2.1 × 100 mm, 3 μm, linear gradient 20%-60% of solvent B over 10 min).


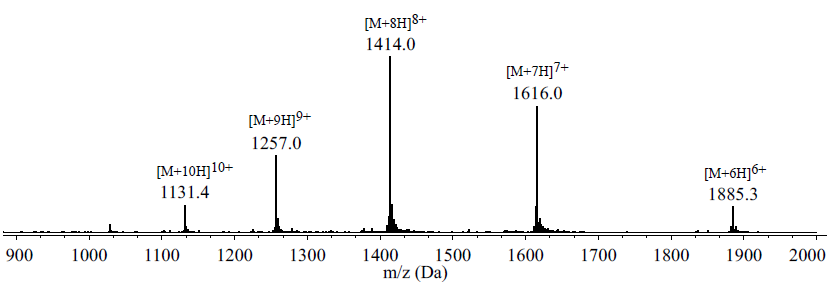


**Figure S22.** Low-resolution MS (ESI) spectrum of purified **18**, Calculated Mass for C_480_H_745_N_99_O_209_S_2_ [M+6H]^6+^: 1885.0; [M+7H]^7+^: 1615.9; [M+8H]^8+^: 1414.0; [M+9H]^9+^: 1257.0; [M+10H]^10+^: 1131.4; Mass Found (ESI+) [M+6H]^6+^: 1885.3; [M+7H]^7+^: 1616.0; [M+8H]^8+^: 1414.0; [M+9H]^9+^: 1257.0; [M+10H]^10+^: 1131.4.

Full sequence **21** was assembled through NCL between glycopeptidyl hydrazide **18** (2.51 mg, 0.22 μmol, 1.1 equiv) and *N*-terminal Cys recombinant fragment **19** (3.94 mg, 0.20 μmol, 1.0 equiv) acccording to **General Procedure E** through two-step reaction in one-pot. The resulting ligation mixture was purified by preparative RP-HPLC (Hedera C4 column, 10 μm, 10 × 250 mm, linear gradient 30%-40% of solvent B over 30 min). The glycosylated full sequence **21** was obtained as a white lyophilized powder (3.21 mg, 52% isolated yield).


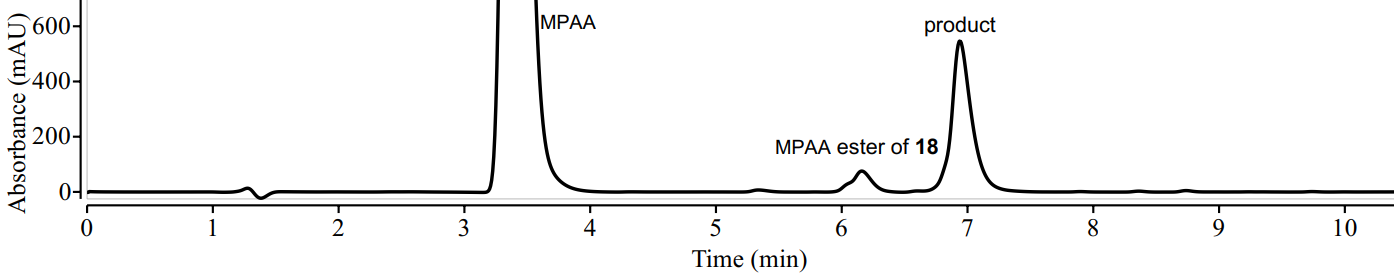


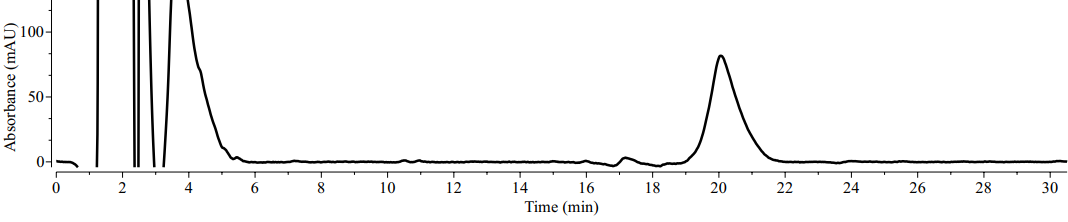


**Figure S23.** Analytical HPLC profile of crude (top) and purified (bottom) **21** (*t*_R_ = 20.1 min, ReproSil-Pur 300Å C4 column, 2.1 × 100 mm, 3 μm, linear gradient 25%-40% of solvent B over 30 min).


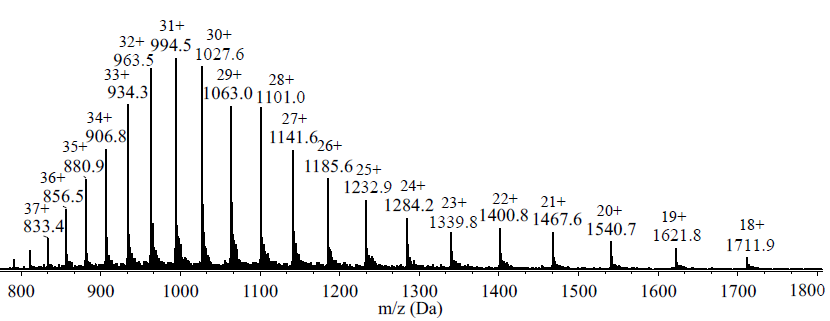


**Figure S24.** Low-resolution MS (ESI) spectrum of purified **21**, Calculated Mass for C_1361_H_2078_N_328_O_470_S_8_: 30811.50 Da (molecular weight), [M+18H]^18+^: 1711.7; [M+19H]^19+^: 1621.7; [M+20H]^20+^: 1540.6; [M+21H]^21+^: 1467.3; [M+22H]^22+^: 1400.7; [M+23H]^23+^: 1339.8; [M+24H]^24+^: 1284.0; [M+25H]^25+^: 1232.7; [M+26H]^26+^: 1185.3; [M+27H]^27+^: 1141.5; [M+28H]^28+^: 1100.7; [M+29H]^29+^: 1062.8; [M+30H]^30+^: 1027.4; [M+31H]^31+^: 994.3; [M+32H]^32+^: 963.3; [M+33H]^33+^: 934.1; [M+34H]^34+^: 906.7; [M+35H]^35+^: 880.8; [M+36H]^36+^: 856.4; [M+37H]^37+^: 833.2; Mass Found (ESI+) [M+18H]^18+^: 1711.9; [M+19H]^19+^: 1621.8; [M+20H]^20+^: 1540.7; [M+21H]^21+^: 1467.6; [M+22H]^22+^: 1400.8; [M+23H]^23+^: 1339.8; [M+24H]^24+^: 1284.2; [M+25H]^25+^: 1232.9; [M+26H]^26+^: 1185.6; [M+27H]^27+^: 1141.6; [M+28H]^28+^: 1101.0; [M+29H]^29+^: 1063.0; [M+30H]^30+^: 1027.6; [M+31H]^31+^: 994.5; [M+32H]^32+^: 963.5; [M+33H]^33+^: 934.3; [M+34H]^34+^: 906.8; [M+35H]^35+^: 880.9; [M+36H]^36+^: 856.5; [M+37H]^37+^: 833.4.


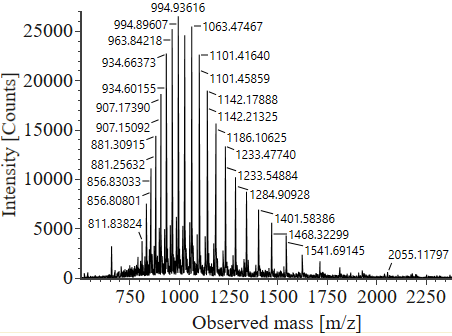

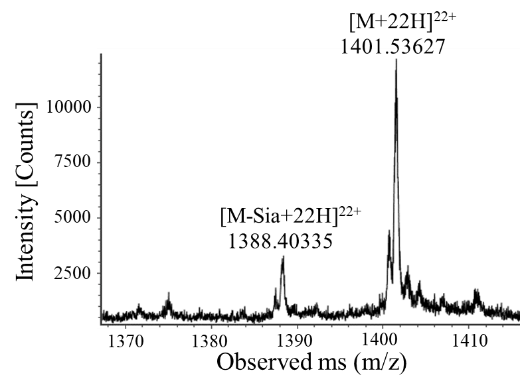


**Figure S25**. High-resolution MS (ESI) full (left) and 22+ charged peaks (right) spectrum of purified **21**, Calculated Mass for C_1361_H_2078_N_328_O_470_S_8_ [M+22H]^22+^: 1401.52352; [M-Sia+22H]^22+^: 1388.28476; Mass Found (ESI+) [M+22H]^22+^: 1401.53627; [M-Sia+22H]^22+^: 1388.40335.

***Note*: Different number of sialic acid (Sia) was disconnected under HRMS condition.**


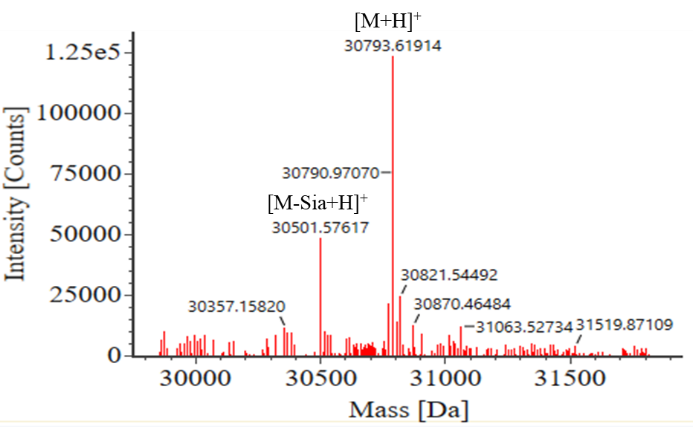


**Figure S26.** High-resolution MS (ESI) spectrum (deconvoluted) of purified **21**, Calculated Mass for C_1361_H_2078_N_328_O_470_S_8_ [M+H]^+^: 30793.66077; [M-Sia+H]^+^: 30501.55983 Mass Found (ESI+) [M+H]^+^: 30793.61914, [M-Sia+H]^+^: 30501.57617.

***Note*: Different number of sialic acid (Sia) was disconnected under HRMS condition.**

Denatured **21** (3.39 mg, 0.11 μM) was refolded and following purified by SEC-HPLC according to **General Procedure F**. The solution of RBD **1** in PBS (1 ×) was stocked for further characterization. The concentration was detected by UV, and the yield of folding was estimated to be 52% (0.93 mg·mL^-1^, 1.88 mL).


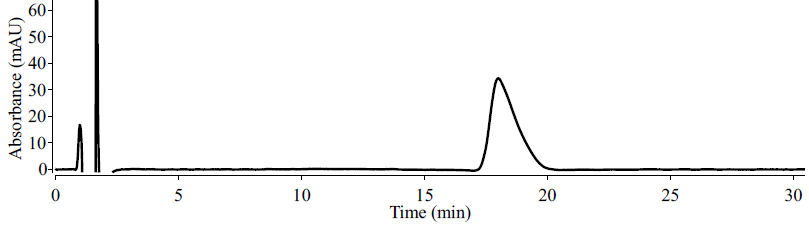


**Figure S27.** Analytical HPLC profile of purified RBD **1** (*t*_R_ = 18.0 min, ReproSil-Pur 300Å C4 column, 2.1 × 100 mm, 3 μm, linear gradient 25%-40% of solvent B over 30 min).


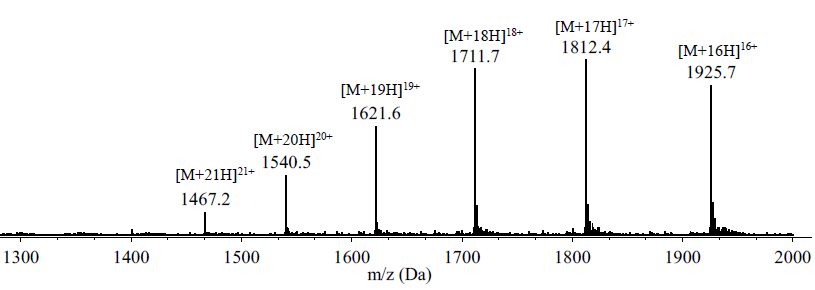


**Figure S28.** Low-resolution MS (ESI) spectrum of purified RBD **1**, Calculated Mass for C_1361_H_2070_N_328_O_470_S_8_: 30803.43 Da (molecular weight), [M+16H]^16+^: 1925.1; [M+17H]^17+^: 1811.9; [M+18H]^18+^: 1711.3; [M+19H]^19+^: 1621.3; [M+20H]^20+^: 1540.2; [M+21H]^21+^: 1466.9; Mass Found (ESI+) [M+16H]^16+^: 1925.7; [M+17H]^17+^: 1812.4; [M+18H]^18+^: 1711.7; [M+19H]^19+^: 1621.6; [M+20H]^20+^: 1540.5; [M+21H]^21+^: 1467.2.


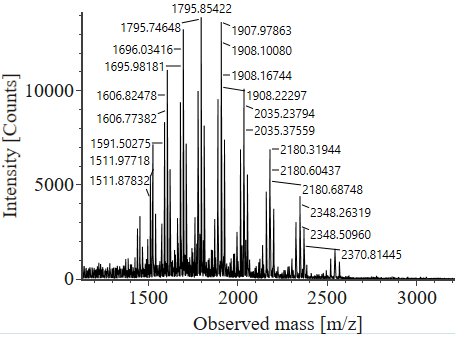

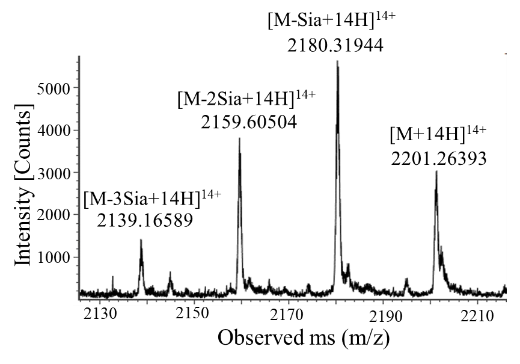


**Figure S29.** High-resolution MS (ESI) full (left) and 14+ charged peaks (right) spectrum of purified RBD **1**, Calculated Mass for C_1361_H_2070_N_328_O_470_S_8_ [M+14H]^14+^: 2201.24593; [M-Sia+14H]^14+^: 2180.44142; [M-2Sia+14H]^14+^: 2159.63716; [M-3Sia+14H]^14+^: 2139.41159; Mass Found (ESI+) [M+14H]^14+^: 2201.26393; [M-Sia+14H]^14+^: 2180.31944; [M-2Sia+14H]^14+^: 2159.60504; [M-3Sia+14H]^14+^: 2139.16589.

***Note*: Different number of sialic acid (Sia) was disconnected under HRMS condition.**


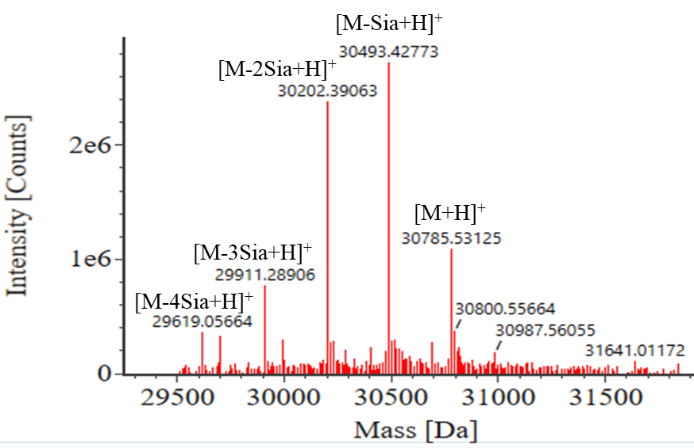


**Figure S30.** High-resolution MS (ESI) spectrum (deconvoluted) of purified RBD **1**, Calculated Mass for C_1361_H_2070_N_328_O_470_S_8_ [M+H]^+^: 30785.59817; [M-Sia+H]^+^: 30493.49723; [M-2Sia+H]^+^: 30202.40181; [M-3Sia+H]^+^: 29911.30639; [M-4Sia+H]^+^: 29620.21097; Mass Found (ESI+) [M+H]^+^: 30785.53125; [M-Sia+H]^+^: 30493.42773; [M-2Sia+H]^+^: 30202.39063; [M-3Sia+H]^+^: 29911.28906; [M-4Sia+H]^+^: 29619.05662.

***Note*: Different number of sialic acid (Sia) was disconnected under HRMS condition.**

## 4. Synthesis, purification and characterization of RBD 2

**Scheme S5:** The synthesis of di-nonasaccharide modified RBD **2**

NCL between glycopeptidyl hydrazide **9** (1.45 mg, 0.40 μmol, 1.1 equiv) and *N*-terminal Cys glycopeptidyl hydrazide **16** (2.36 mg, 0.36 μmol, 1.0 equiv) was conducted acccording to **General Procedure D** through two-step reaction in one-pot to assemble glycopeptidyl hydrazide **S5**. The resulting ligation mixture was purified by preparative RP-HPLC (Dubhe C18 column, 10 μm, 10 × 250 mm, linear gradient 20%-45% of solvent B over 30 min). The glycopeptidyl hydrazide **S5** was obtained as a white lyophilized powder (1.86 mg, 51% isolated yield).


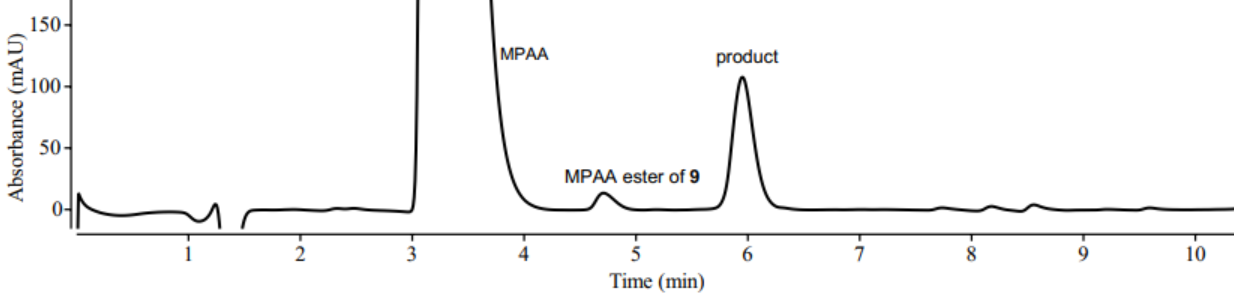


**
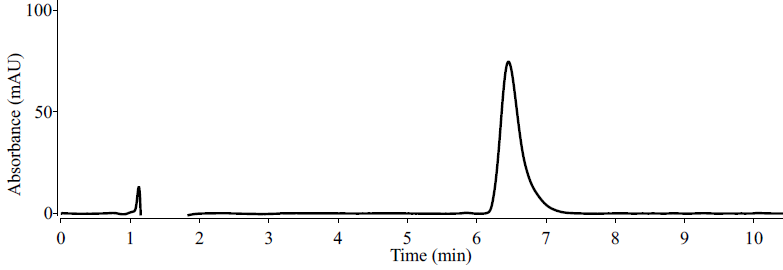
**

**Figure S31.** Analytical HPLC profile of purified **S5** (*t*_R_ = 6.5 min, Hedera C18 column, 2.1 × 100 mm, 3 μm, linear gradient 20%-50% of solvent B over 10 min).

**
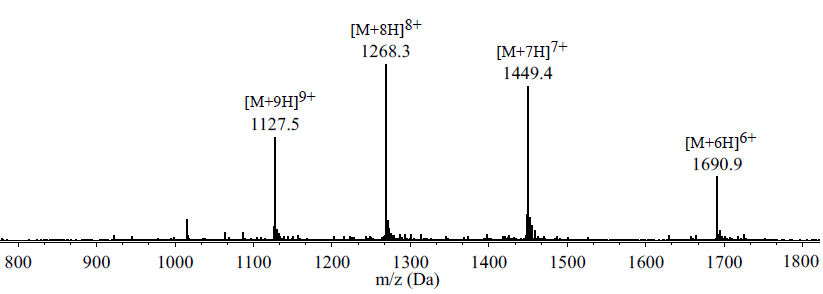
**

**Figure S32.** Low-resolution MS (ESI) spectrum of purified **S5**, Calculated Mass for C_436_H_677_N_95_O_177_S_2_ [M+6H]^6+^:1690.9; [M+7H]^7+^: 1449.5; [M+8H]^8+^: 1268.5 [M+9H]^9+^: 1127.6; Mass Found (ESI+) [M+6H]^6+^:1690.9; [M+7H]^7+^: 1449.4; [M+8H]^8+^: 1268.3 [M+9H]^9+^: 1127.5.

Full sequence of **S6** was assembled through NCL between glycopeptidyl hydrazide **S5** (2.64 mg, 0.26 μmol, 1.1 equiv) and *N*-terminal Cys recombinant fragment **19** (4.54 mg, 0.23 μmol, 1.0 equiv) acccording to **General Procedure D** through two-step reaction in one-pot. The resulting ligation mixture was purified by preparative RP-HPLC (Hedera C4 column, 10 μm, 10 × 250 mm, linear gradient 30%-40% of solvent B over 30 min). The glycosylated full sequence **S6** was obtained as a white lyophilized powder (3.89 mg, 57% isolated yield).


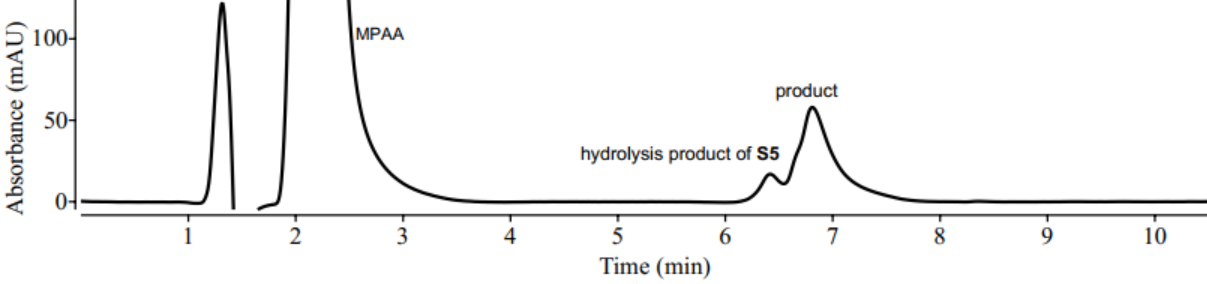


**
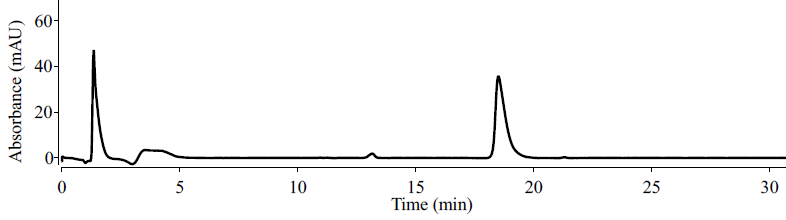
**

**Figure S33.** Analytical HPLC profile of crude (top) and purified (bottom) **S6** (*t*_R_ = 18.7 min, ReproSil-Pur 300Å C4 column, 2.1 × 100 mm, 3 μm, linear gradient 25%-40% of solvent B over 10 min).


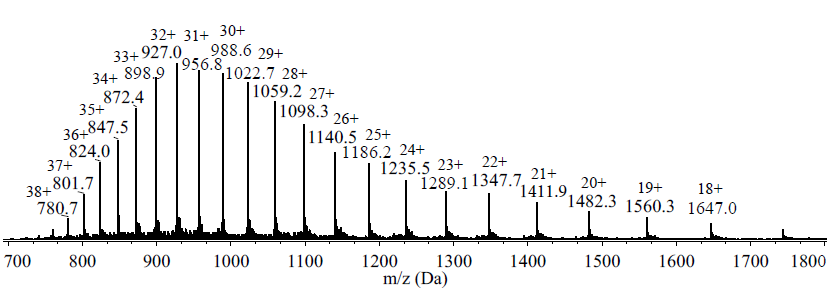


**Figure S34.** Low-resolution MS (ESI) spectrum of purified **S6**, Calculated Mass for C_1317_H_2010_N_324_O_438_S_8_: 29646.50 Da (molecular weight), [M+18H]^18+^: 1647.0; [M+19H]^19+^: 1560.4; [M+20H]^20+^: 1482.4; [M+21H]^21+^: 1411.9; [M+22H]^22+^: 1347.7; [M+23H]^23+^: 1289.2; [M+24H]^24+^: 1235.5; [M+25H]^25+^: 1186.1; [M+26H]^26+^: 1140.5; [M+27H]^27+^: 1098.3; [M+28H]^28+^: 1059.2; [M+29H]^29+^: 1022.7; [M+30H]^30+^: 988.6; [M+31H]^31+^: 956.8; [M+32H]^32+^: 926.9; [M+33H]^33+^: 898.8; [M+34H]^34+^: 872.4; [M+35H]^35+^: 847.5; [M+36H]^36+^: 824.0; [M+37H]^37+^: 801.8; [M+38H]^38+^: 780.7; Mass Found (ESI+) [M+18H]^18+^: 1647.0; [M+19H]^19+^: 1560.3; [M+20H]^20+^: 1482.3; [M+21H]^21+^: 1411.9; [M+22H]^22+^: 1347.7; [M+23H]^23+^: 1289.1; [M+24H]^24+^: 1235.5; [M+25H]^25+^: 1186.2; [M+26H]^26+^: 1140.5; [M+27H]^27+^: 1098.3; [M+28H]^28+^: 1059.2; [M+29H]^29+^: 1022.7; [M+30H]^30+^: 988.6; [M+31H]^31+^: 956.8; [M+32H]^32+^: 927.0; [M+33H]^33+^: 898.9; [M+34H]^34+^: 872.4; [M+35H]^35+^: 847.5; [M+36H]^36+^: 824.0; [M+37H]^37+^: 801.7; [M+38H]^38+^: 780.7.


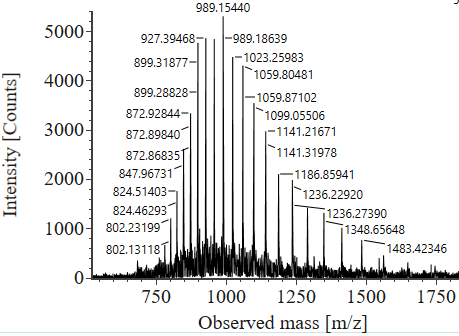

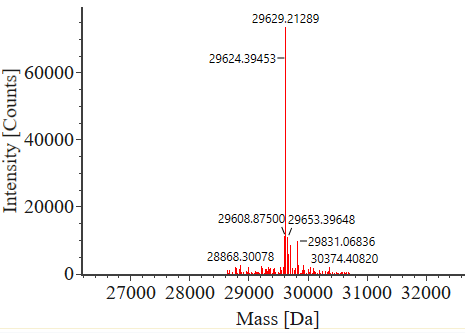


**Figure S35.** High-resolution MS (ESI) spectrum of purified **S6**, Calculated Mass for C_1317_H_2010_N_324_O_438_S_8_ [M+H]^+^: 29629.27920; Mass Found (ESI+) [M+H]^+^: 29629.21289 (deconvoluted).

Denatured **S6** (2.96 mg, 0.10 μM) was refolded and following purified by SEC-HPLC according to **General Procedure F**. The solution of RBD **2** in PBS (1 ×) was stocked for further characterization, the yield of folding was estimated to be 51% (0.79 mg·mL^-1^, 1.91 mL).


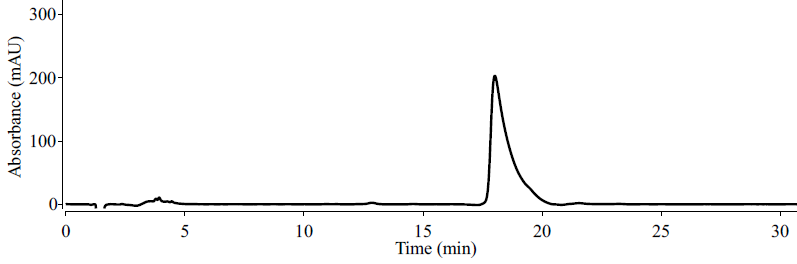


**Figure S36.** Analytical HPLC profile of purified RBD **2** (*t*_R_ = 18.3 min, ReproSil-Pur 300Å C4 column, 2.1 × 100 mm, 3 μm, linear gradient 25%-40% of solvent B over 30 min).


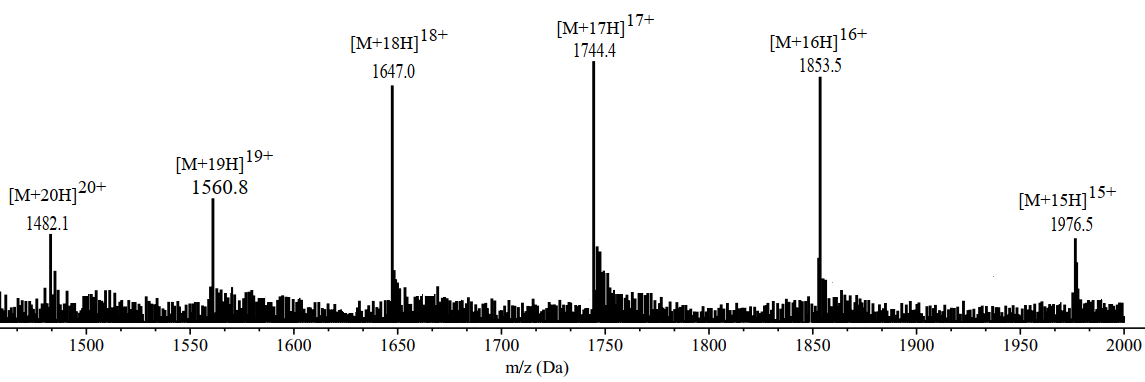


**Figure S37.** Low-resolution MS (ESI) spectrum of purified RBD **2**, Calculated Mass for C_1317_H_2002_N_324_O_438_S_8_: 29638.42 Da (molecular weight), [M+15H]^15+^: 1976.9; [M+16H]^16+^: 1853.4; [M+17H]^17+^: 1744.4; [M+18H]^18+^: 1647.6; [M+19H]^19+^: 1560.9; [M+20H]^20+^: 1482.9; Mass Found (ESI+) [M+15H]^15+^: 1976.5; [M+16H]^16+^: 1853.5; [M+17H]^17+^: 1744.4; [M+18H]^18+^: 1647.0; [M+19H]^19+^: 1560.8; [M+20H]^20+^: 1482.1.


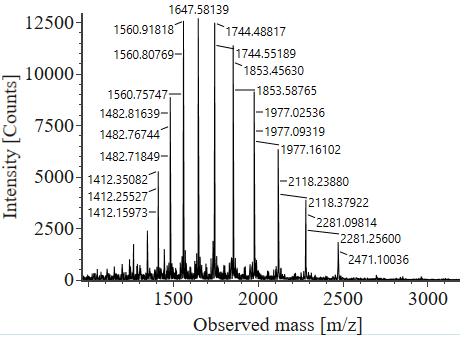

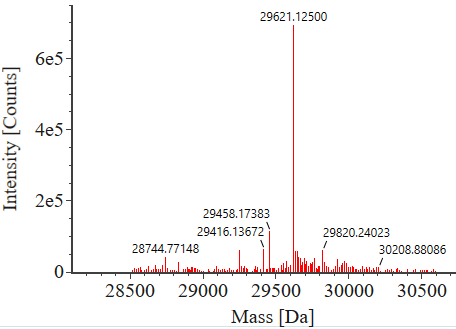


**Figure S38.** High-resolution MS (ESI) spectrum of purified RBD **2**, Calculated Mass for C_1317_H_2002_N_324_O_438_S_8_ [M+H]^+^: 29621.21660; Mass Found (ESI+) [M+H]^+^: 29621.12500 (deconvoluted).

## 5. Synthesis, purification and characterization of RBD 3

 **Scheme S6:** The synthesis of tri-undecasaccharide modified RBD **3**

Amino acids containing acid-labile protecting groups are marked in bold in the schemes. Protecting groups are: R(Pbf), Q(Trt), E(*t*Bu), N(Trt).

Fmoc-Leu-OH pre-loaded resins (0.50 g, 0.66 mmol·g^-1^) were used to synthesize resin-bound peptide **S7** according to generally manual SPPS protocols described in **General Procedure A**. Allyl (All) protected Fmoc-Asp(All)-OH and pseudoproline dipeptide Fmoc-Glu-Ser(ψ^Me,Me^Pro)-OH, Fmoc-Ile-Thr(ψ^Me,Me^Pro)-OH were introduced in the corresponding sites [8].

Resin-bound peptide **S7** was subjected to conservative cleavage with diluted TFA solution (1% in DCM, v/v) according to **General Procedure A** to give crude protected peptidyl hydrazide (1.01 g). The crude product was used in the next step without further purification.

The crude peptidyl hydrazide **S7** was dissolved in a mixture of THF (6 mL) and saturated NaHCO_3_ aqueous solution (3 mL), Boc_2_O (0.76 mL, 3.30 mmol, 10.0 equiv, based on initial loading value) were following added. The mixture was further stirred vigorously at 25 ºC for 6 hours. After the reaction completed, the reaction mixture was diluted with AcOEt (50 mL), the organic phase was seperated and dried over Na_2_SO_4_. After filtration, the solution was concentrated under reduced pressure to give crude protected peptide. The residue was further purified by silica gel column chromatography (DMC/MeOH, 50/1-40/1, v/v, with trace trimethylamine) to afford allyl protected peptide **S8** (1.13 g, 70% yield, based on initial loading value) as a white solid.


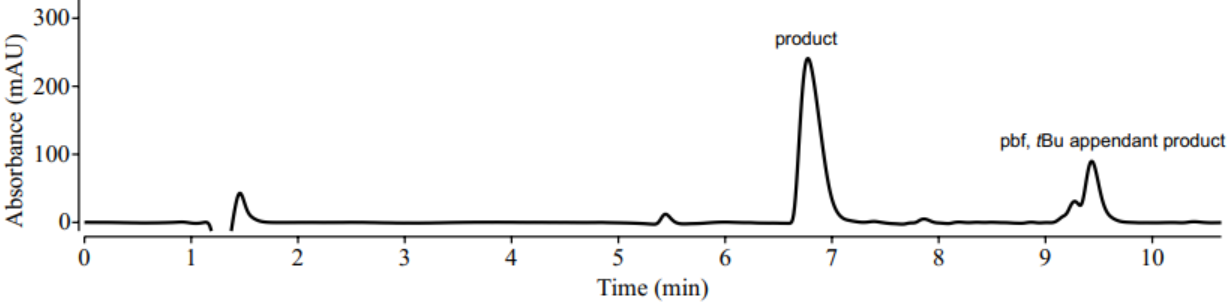


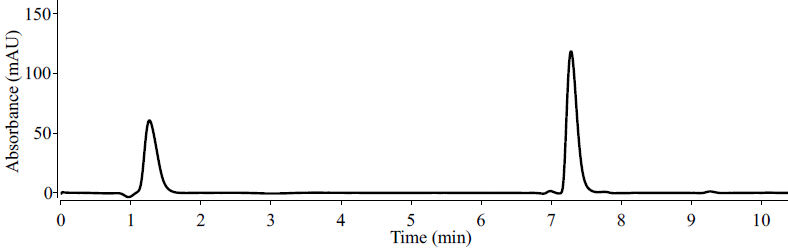


**Figure S39.** Analytical HPLC profile of crude (top) and purified (bottom) **S8** after globally deprotected with Cocktail B (*t*_R_ = 7.3 min, Hedera C18 column, 300 Å, 2.1 × 100 mm, 3 μm, linear gradient 10%-52% of solvent B over 10 min).


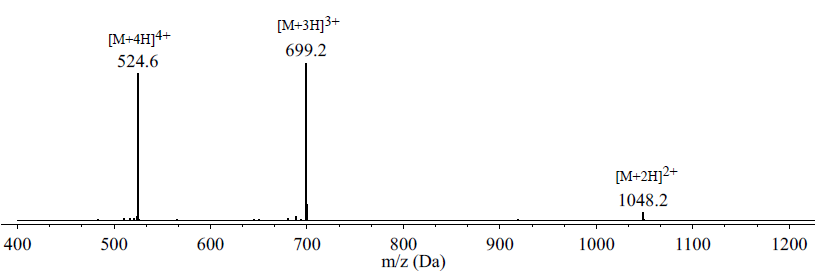


**Figure S40.** Low-resolution MS (ESI) spectrum of purified **S8** after globally deprotected with Cocktail B, Calculated Mass for C_94_H_153_N_27_O_27_ [M+2H]^2+^: 1047.7; [M+3H]^3+^: 698.8; [M+4H]^4+^: 524.4; Mass Found (ESI+) [M+2H]^2+^: 1048.2; [M+3H]^3+^: 699.2; [M+4H]^4+^: 524.6.

To a solution of allyl protected peptide **S8** (0.45 g, 0.13 mmol, 1.0 equiv) in DCM (3 mL), Pd(PPh_3_)_4_ (75 mg, 65.0 μmol, 0.5 equiv) and PhSiH_3_ (160 μL, 1.30 mmol, 10.0 equiv) were added. The mixture was kept stirring at 25 ºC for 1 hour. During the progress, the reaction mixture turned from light yellow to dark brown. Upon completion, the reaction mixture was concentrated under reduced pressure, then further purified by silica gel column chromatography (DMC/MeOH, 30/1-10/1, v/v) to afford protected peptidyl acid **S9** (0.41 g, 95% isolated yield) as a gray solid.


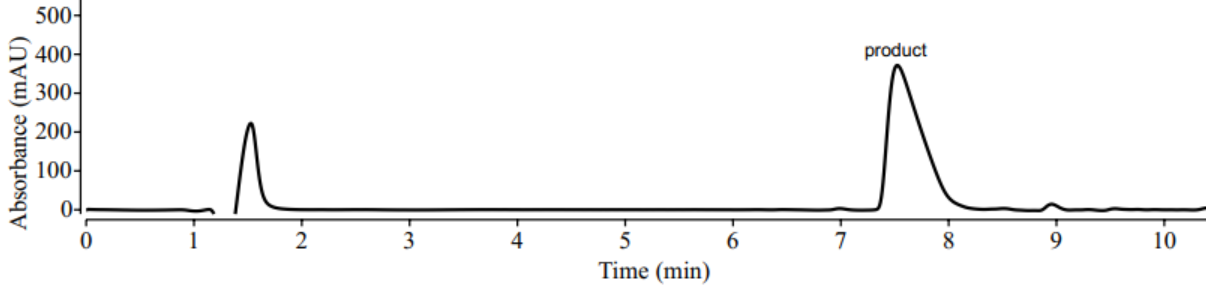


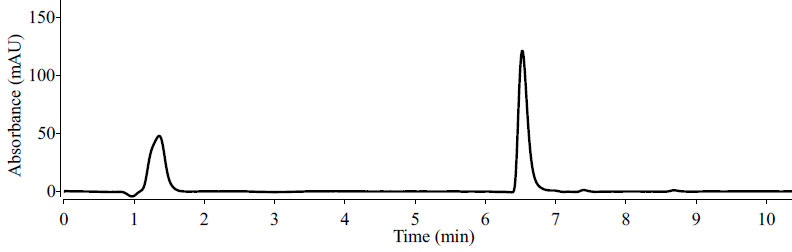


**Figure S41.** Analytical HPLC profile of crude (top) and purified (bottom) **S9** after globally deprotected with Cocktail B (*t*_R_ = 6.6 min, Hedera C18 column, 300 Å, 2.1 × 100 mm, 3 μm, linear gradient 10%-52% of solvent B over 10 min).


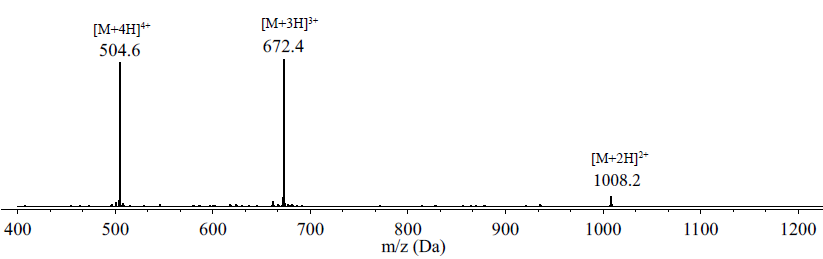


**Figure S42.** Low-resolution MS (ESI) spectrum of purified **S9** after globally deprotected with Cocktail B, Calculated Mass for C_88_H_145_N_27_O_27_ [M+2H]^2+^: 1007.7; [M+3H]^3+^: 672.1; [M+4H]^4+^: 504.3; Mass Found (ESI+) [M+2H]^2+^: 1008.2; [M+3H]^3+^: 672.4; [M+4H]^4+^: 504.6.

Protected peptidyl acid **S9** (35.0 mg, 12.4 μmol, 1.0 equiv) was glycosylated with nonasaccharide amine (61.0 mg, 37.2 μmol, 3.0 equiv) in the presence of HATU (18.8 mg, 49.6 μmol, 4.0 equiv), HOBt (7.03 mg, 52.1 μmol, 4.2 equiv) and DIPEA (17.2 μL, 99.2 μmol, 8.0 equiv) in anhydrous DMSO (50 μL). The reaction was kept stirring vigorously at 25 ºC for 4 hours. After the reaction completed, the protecting groups were globally cleaved by addition of Cocktail B according to **General Procedure A**. The crude product was purified by preparative RP-HPLC (Dubhe C18 column, 10 μm, 20 × 250 mm, linear gradient 15%-30% of solvent B over 30 min). Glycopeptide **S10** was obtained as a white lyophilized powder (29.3 mg, 45% isolated yield).


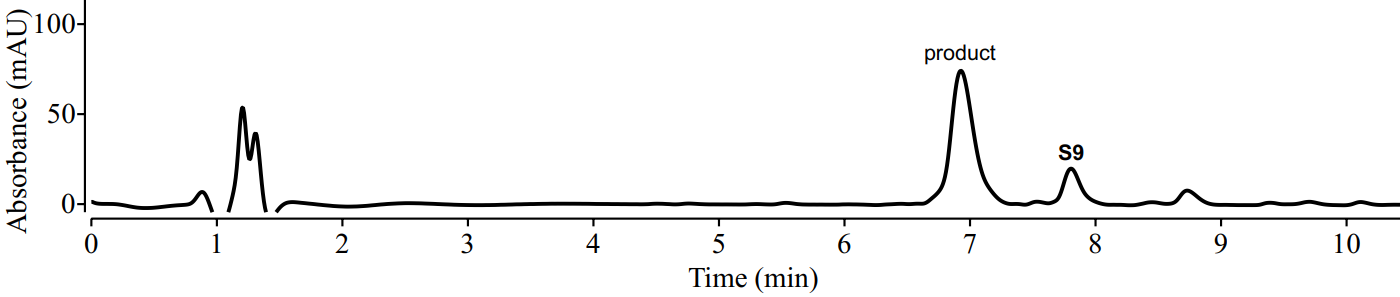


**
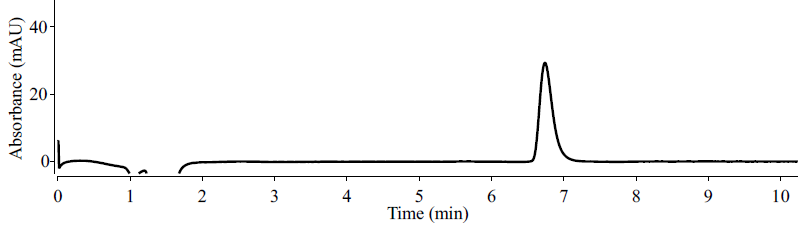
**

**Figure S43.** Analytical HPLC profile of crude (top) and purified (bottom) **S10** (*t*_R_ = 6.7 min, Hedera C18 column, 2.1 × 100 mm, 3 μm, linear gradient 10%-40% of solvent B over 10 min).

**
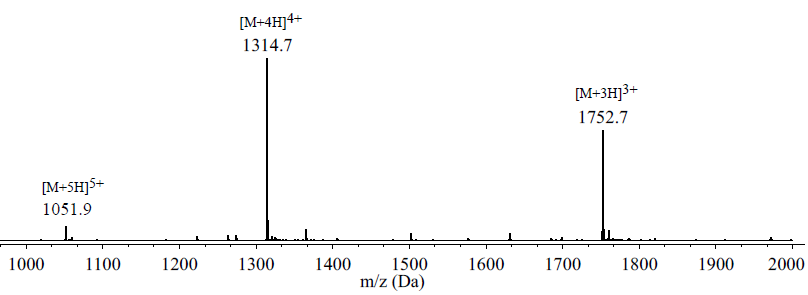
**

**Figure S44.** Low-resolution MS (ESI) spectrum of purified **S10**, Calculated Mass for C_212_H_351_N_37_O_115_ [M+3H]^3+^:1752.8; [M+4H]^4+^: 1314.8 [M+5H]^5+^: 1052.1; Mass Found (ESI+) [M+3H]^3+^:1752.7; [M+4H]^4+^: 1314.7 [M+5H]^5+^: 1051.9.

Glycopeptidyl hydrazide **S11** was synthesized by enzyme catalytic reaction according to the protocol described in **General Procedure C** with nonasaccharide modified glycopeptidyl hydrazide **S10** (12.1 mg, 3.34 μM, 1.0 equiv), in the presence of Neu5Ac (8.0 equiv), CTP (10.0 equiv), *PmST3* (final Conc. 0.60 mg∙mL^-1^) and *NmCSS* (final Conc. 0.60 mg ∙mL^-1^). The resulting reaction mixture was purified by preparative RP-HPLC (Dubhe C18 column, 10 μm, 20 × 250 mm, linear gradient 15%-30% of solvent B over 30 min). The undecasaccharide modified glycopeptidyl hydrazide **S11** was obtained as a white lyophilized powder (17.2 mg, 80% isolated yield).


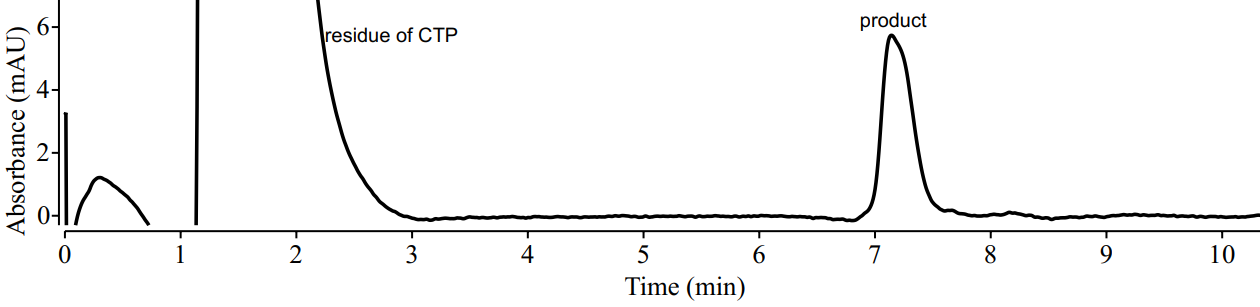


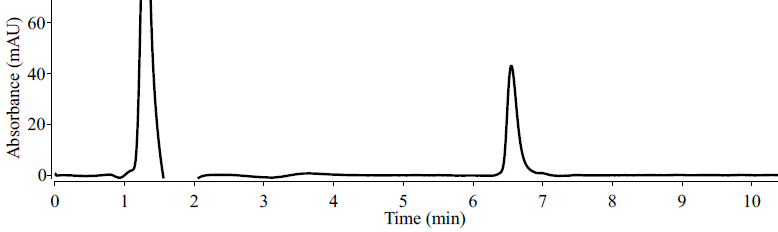


**Figure S45.** Analytical HPLC profile of crude (top) and purified (bottom) **S11** (*t*_R_ = 6.6 min, Hedera C18 column, 2.1 × 100 mm, 3 μm, linear gradient 10%-52% of solvent B over 10 min).


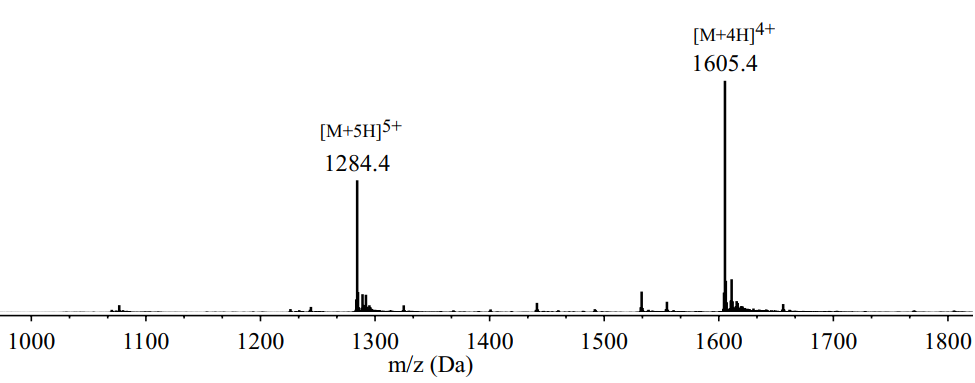


**Figure S46.** Low-resolution MS (ESI) spectrum of purified **S11**, Calculated Mass for C_256_H_419_N_41_O_147_ [M+4H]^4+^: 1605.9 [M+5H]^5+^: 1284.9; Mass Found (ESI+) [M+4H]^4+^: 1605.4 [M+5H]^5+^: 1284.4.

NCL between glycopeptidyl hydrazide **S11** (3.11 mg, 0.48 μmol, 1.1 equiv) and *N*-terminal Cys glycopeptidyl hydrazide **17** (3.14 mg, 0.44 μmol, 1.0 equiv) was conducted acccording to **General Procedure D** through two-step reaction in one-pot to assemble glycopeptidyl hydrazide **S12**. The resulting ligation mixture was purified by preparative RP-HPLC (Hedera C4 column, 10 μm, 10 × 250 mm, linear gradient 20%-35% of solvent B over 30 min). The glycopeptidyl hydrazide **S12** was obtained as a white lyophilized powder (2.92 mg, 45% isolated yield).


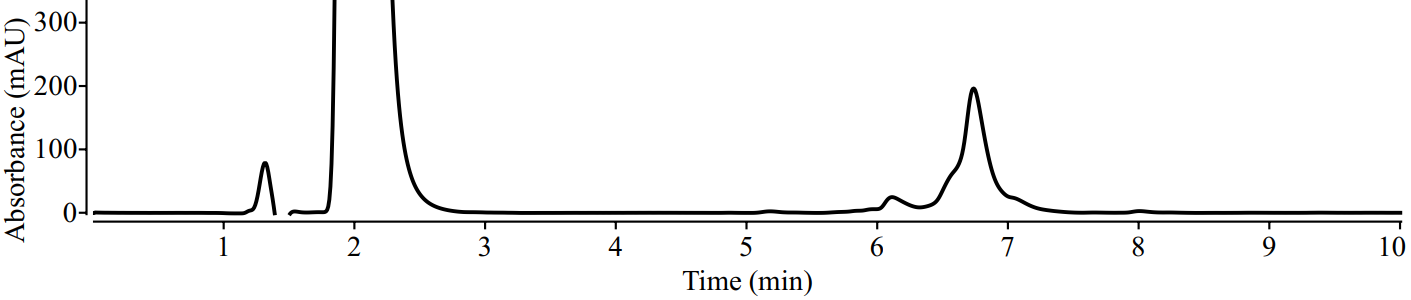


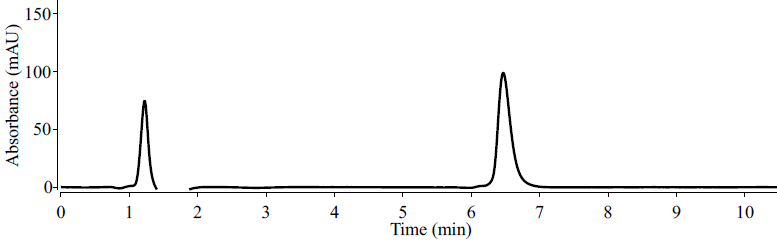


**Figure S47.** Analytical HPLC profile of crude (top) and purified (bottom) **S12** (*t*_R_ = 6.5 min, ReproSil-Pur 300Å C4 column, 2.1 × 100 mm, 3 μm, linear gradient 20%-60% of solvent B over 10 min).


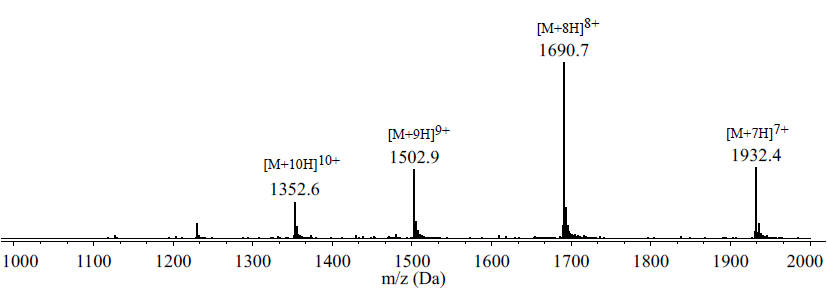


**Figure S48.** Low-resolution MS (ESI) spectrum of purified **S12**, Calculated Mass for C_564_H_880_N_106_O_270_S_2_ [M+7H]^7+^: 1932.7; [M+8H]^8+^: 1691.2; [M+9H]^9+^: 1503.4; [M+10H]^10+^: 1353.2; Mass Found (ESI+) [M+7H]^7+^: 1932.7; [M+8H]^8+^: 1690.7; [M+9H]^9+^: 1502.9; [M+10H]^10+^: 1352.6.

Full sequence of tri-undecasaccharide modified **S13** was assembled through NCL between glycopeptidyl hydrazide **S12** (2.70 mg, 0.20 μmol, 1.1 equiv) and *N*-terminal Cys recombinant fragment **19** (3.51 mg, 0.18 μmol, 1.0 equiv) acccording to **General Procedure D** through two-step reaction in one-pot. The resulting ligation mixture was purified by preparative RP-HPLC (Hedera C4 column, 10 μm, 10 × 250 mm, linear gradient 28%-35% of solvent B over 30 min). The glycosylated full sequence **S13** was obtained as a white lyophilized powder (2.44 mg, 41% isolated yield).


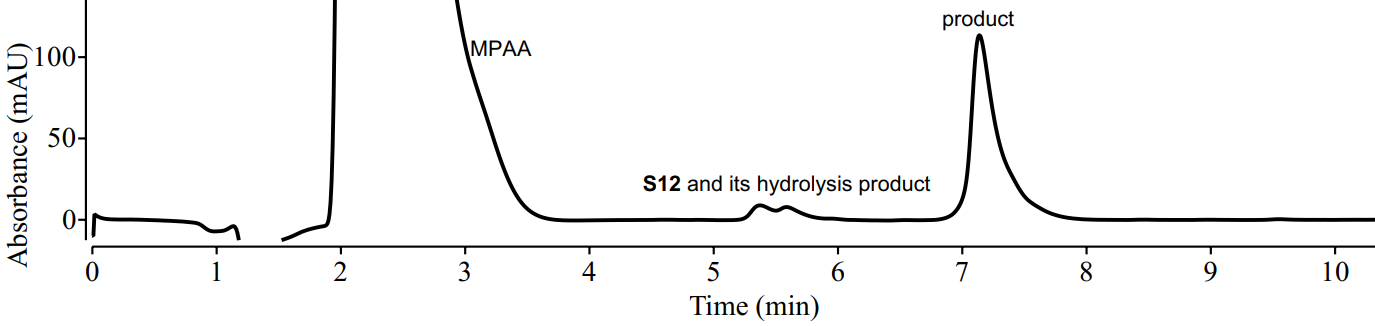

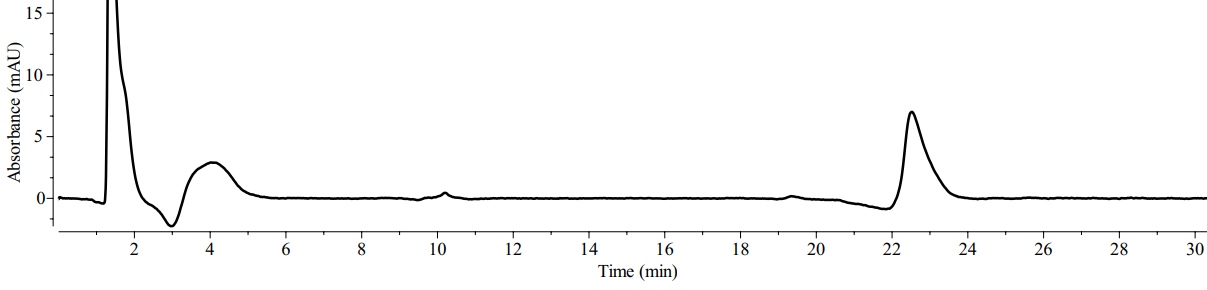


**Figure S49.** Analytical HPLC profile of crude (top) and purified (bottom) **S13** (*t*_R_ = 22.5 min, ReproSil-Pur 300Å C4 column, 2.1 × 100 mm, 3 μm, linear gradient 20%-40% of solvent B over 30 min).


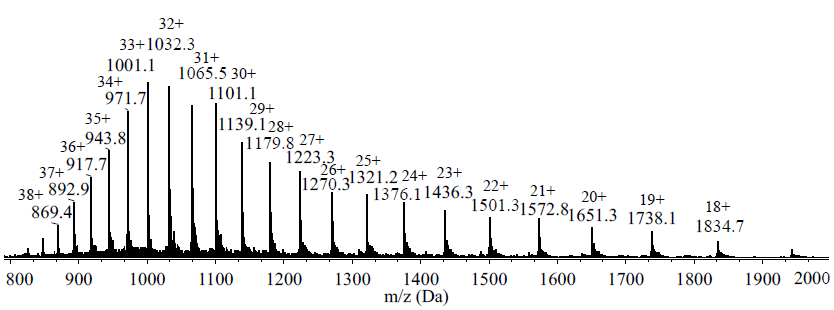


**Figure S50.** Low-resolution MS (ESI) spectrum of purified **S13**, Calculated Mass for C_1445_H_2213_N_335_O_531_S_8_: 33030.48 Da (molecular weight), [M+18H]^18+^: 1834.9; [M+19H]^19+^: 1738.4; [M+20H]^20+^: 1651.5; [M+21H]^21+^: 1572.9; [M+22H]^22+^: 1501.5; [M+23H]^23+^: 1436.2; [M+24H]^24+^: 1376.4; [M+25H]^25+^: 1321.4; [M+26H]^26+^: 1270.6; [M+27H]^27+^: 1223.6; [M+28H]^28+^: 1179.9; [M+29H]^29+^: 1139.3; [M+30H]^30+^: 1101.3; [M+31H]^31+^: 1065.9; [M+32H]^32+^: 1032.6; [M+33H]^33+^: 1001.3; [M+34H]^34+^: 971.9; [M+35H]^35+^: 944.2; [M+36H]^36+^: 918.0; [M+37H]^37+^: 893.2; [M+38H]^38+^: 869.7; Mass Found (ESI+) [M+18H]^18+^: 1834.7; [M+19H]^19+^: 1738.1; [M+20H]^20+^: 1651.3; [M+21H]^21+^: 1572.8; [M+22H]^22+^: 1501.3; [M+23H]^23+^: 1436.3; [M+24H]^24+^: 1376.1; [M+25H]^25+^: 1321.2; [M+26H]^26+^: 1270.3; [M+27H]^27+^: 1223.3; [M+28H]^28+^: 1179.8; [M+29H]^29+^: 1139.1; [M+30H]^30+^: 1101.1; [M+31H]^31+^: 1065.5; [M+32H]^32+^: 1032.3; [M+33H]^33+^: 1001.1; [M+34H]^34+^: 971.7; [M+35H]^35+^: 943.8; [M+36H]^36+^: 917.7; [M+37H]^37+^: 892.9; [M+38H]^38+^: 869.4.


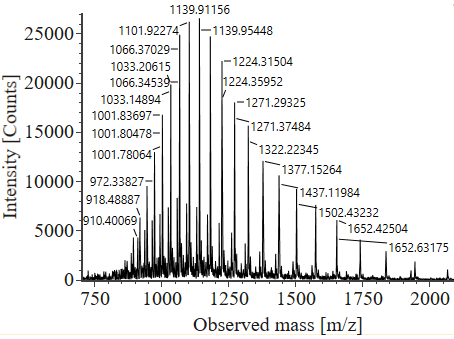

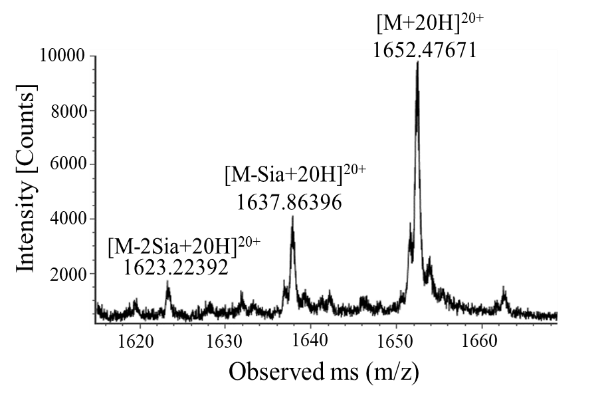


**Figure S51.** High-resolution MS (ESI) full (left) and 20+ charged peaks (right) spectrum of purified **S13**, Calculated Mass for C_1445_H_2213_N_335_O_531_S_8_ [M+20H]^20+^: 1652.52415; [M-Sia+20H]^20+^: 1637.96123; [M-2Sia+20H]^20+^: 1623.19784; Mass Found (ESI+) [M+20H]^20+^: 1652.47671; [M-Sia+20H]^20+^: 1637.86396; [M-2Sia+20H]^20+^: 1623.22392.

***Note*: Different number of sialic acid (Sia) was disconnected under HRMS condition.**


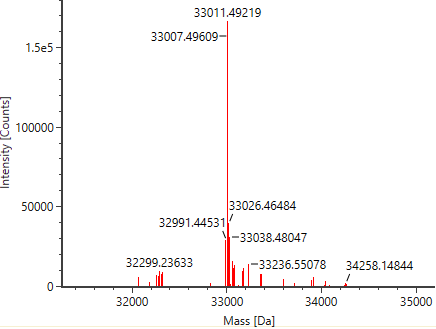


**Figure S52**. High-resolution MS (ESI) spectrum of purified **S13**, Calculated Mass for C_1445_H_2213_N_335_O_531_S_8_ [M+H]^+^: 33011.42818; Mass Found (ESI+) [M+H]^+^: 33011.49219.

Denatured **S13** (3.31 mg, 0.10 μM) was refolded and following purified by SEC-HPLC according to **General Procedure F**. The solution of RBD **3** in PBS (1 ×) was stocked for further characterization, the yield of folding was estimated to be 47% (0.96 mg·mL^-1^, 1.62 mL).


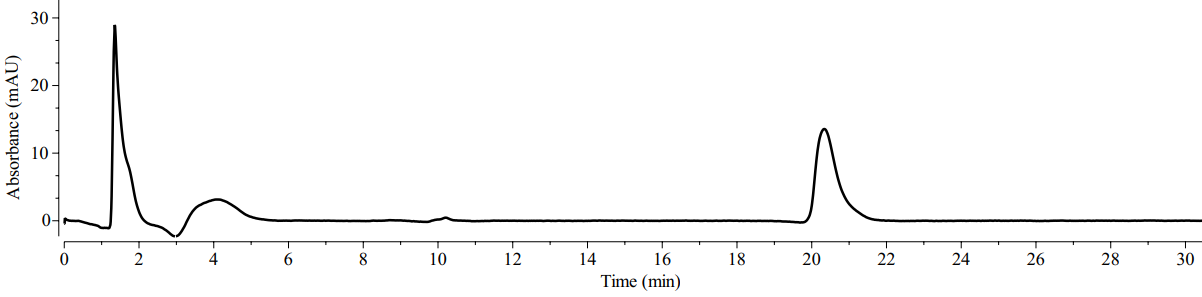


**Figure S53.** Analytical HPLC profile of purified **RBD 3** (*t*_R_ = 20.4 min, ReproSil-Pur 300Å C4 column, 2.1 × 100 mm, 3 μm, linear gradient 20%-40% of solvent B over 30 min).


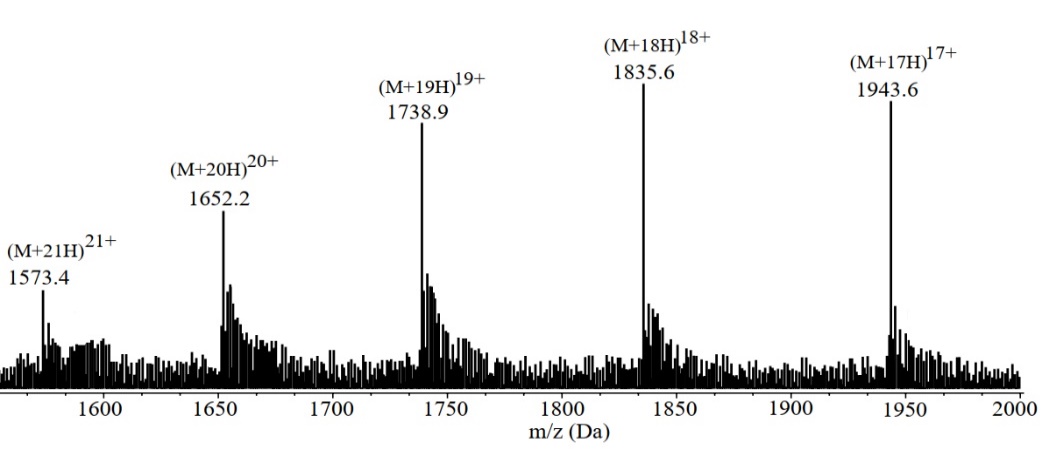


**Figure S54.** Low-resolution MS (ESI) spectrum of purified RBD **3**, Calculated Mass for C_1445_H_2213_N_335_O_531_S_8_: 33022.42 Da (molecular weight), [M+17H]^17+^: 1943.5; [M+18H]^18+^: 1835.6; [M+19H]^19+^: 1739.0; [M+20H]^20+^: 1652.1; [M+21H]^21+^: 1573.5; Mass Found (ESI+) [M+17H]^17+^: 1943.6; [M+18H]^18+^: 1835.6; [M+19H]^19+^: 1738.9; [M+20H]^20+^: 1652.2; [M+21H]^21+^: 1573.4.


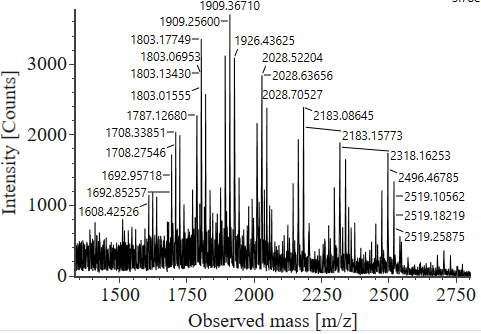

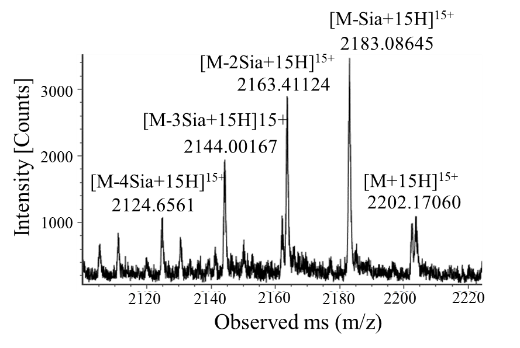


**Figure S55.** High-resolution MS (ESI) full (left) and 15+ charged peaks (right) spectra of purified RBD **3**, Calculated Mass for C_1445_H_2205_N_335_O_531_S_8_ [M+15H]^15+^: 2202.47602; [M-Sia+15H]^15+^: 2183.07767; [M-2Sia+15H]^15+^: 2163.39285; [M-3Sia+15H]^15+^: 2143.97589; [M-4Sia+15H]^15+^: 2124.55858; Mass Found [M+15H]^15+^: 2202.17060; [M-Sia+15H]^15+^: 2183.08645; [M-2Sia+15H]^15+^: 2163.41124; [M-3Sia+15H]^15+^: 2144.00167; [M-4Sia+15H]^15+^: 2124.65613.

***Note*: Different number of sialic acid (Sia) was disconnected under HRMS condition.**


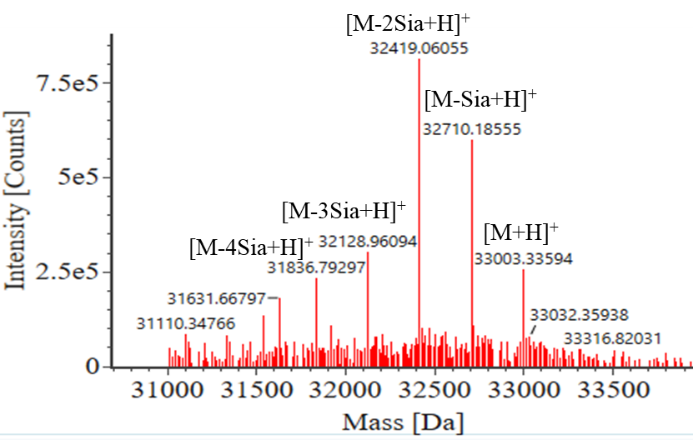


**Figure S56.** High-resolution MS (ESI) spectrum (deconvoluted) of purified RBD **3**, Calculated Mass for C_1445_H_2205_N_335_O_531_S_8_ [M+H]^+^: 33003.36658; [M-Sia+H]^+^: 32710.25711; [M-2Sia+H]^+^: 32419.16169; [M-3Sia+H]^+^: 32128.06627; [M-4Sia+H]^+^: 31836.97086; Mass Found (ESI+) [M+H]^+^: 33003.33594; [M-Sia+H]^+^: 32710.18555; [M-2Sia+H]^+^: 32419.06055; [M-3Sia+H]^+^: 32128.79297; [M-4Sia+H]^+^: 31836.79297.

***Note*: Different number of sialic acid (Sia) was disconnected under HRMS condition.**

## 6. Refolding, purification and characterization of RBD 4

**Scheme S7:** The synthesis of di-nonasaccharide modified RBD **4**

Denatured **S14** (3.12 mg, 0.11 μM, synthesized according to our previous work [4]) was refolded and following purified by SEC-HPLC according to **General Procedure F**. The solution of RBD **4** in PBS (1 ×) was stocked for further characterization, the yield of folding was estimated to be 49% (0.84 mg·mL^-1^, 1.81 mL). Spectral data are in accordance with those reported in our previous work [4].

## 7. Synthesis, purification and characterization of RBD 5

**Scheme S8:** The synthesis of di-nonasaccharide modified Omicron variant RBD **5**

Amino acids containing acid-labile protecting groups are marked in bold in the schemes. Protecting groups are: R(Pbf), Q(Trt), C(Trt), D(*t*Bu), E(*t*Bu), W(Boc), N(Trt), S(*t*Bu), Y(*t*Bu) , T(*t*Bu), K(Boc).

Full nucleotides sequence (forward, the cleavage sites are marked in bold) and amino acids

sequence of Omicron RBD C379-K537 **S20** were listed as follow.

**CATATG**TGCTACGGCGTGAGCCCCACCAAGCTGAACGACCTGTGCTTCACCAACGTGTACGCCGACAGCTTCGTGATCAGGGGCGACGAGGTGAGGCAGATCGCCCCCGGCCAGACCGGCAACATCGCCGACTACAACTACAAGCTGCCCGACGACTTCACCGGCTGCGTGATCGCCTGGAACAGCAACAAGCTGGACAGCAAGGTGAGCGGCAACTACAACTACCTGTACAGGCTGTTCAGGAAGAGCAACCTGAAGCCCTTCGAGAGGGACATCAGCACCGAGATCTACCAGGCCGGCAACAAGCCCTGCAACGGCGTGGCCGGCTTCAACTGCTACTTCCCCCTGAGGAGCTACAGCTTCAGGCCCACCTACGGCGTGGGCCACCAGCCCTACAGGGTGGTGGTGCTGAGCTTCGAGCTGCTGCACGCCCCCGCCACCGTGTGCGGCCCCAAGAAGAGCACCAACCTGGTGAAGAACAAG**CTCGAG**

**Figure S57.** Full nucleotides (top) and amino acids sequence (bottom) of Omicron RBD C379-K537 **S20**

The plasmid pET21a-omicron-rbd was employed to recombine Omicron RBD C379-K537 **S20** according to the protocol described in **General Procedure B**. Supernatant containing Omicron-RBD fragment III (379-537) was purified by preparative RP-HPLC (Hedera C4 column, 10 μm, 20 × 250 mm, linear gradient 30%-40% of solvent B over 30 min). **S20** was obtained as a white lyophilized powder (31 mg·L^-1^, isolated yield).


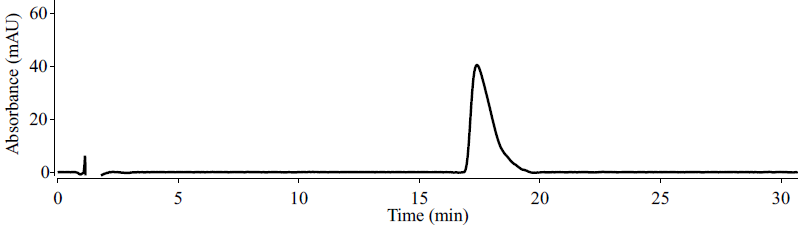


**Figure S58.** Analytical HPLC profile of purified **S20** (*t*_R_ = 17.5 min, ReproSil-Pur 300Å C4 column, 2.1 × 100 mm, 3 μm, linear gradient 20%-40% of solvent B over 30 min).


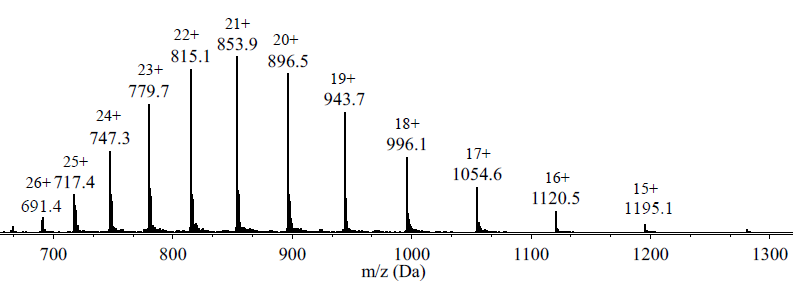


**Figure S59.** Low-resolution MS (ESI) spectrum of purified **S20**, Calculated Mass for Calculated Mass for C_808_H_1229_N_217_O_233_S_6_: 17903.11 Da (molecular weight), [M+15H]^15+^: 1194.6; [M+16H]^16+^: 1120.0; [M+17H]^17+^: 1054.1; [M+18H]^18+^: 995.6; [M+19H]^19+^: 943.3; [M+20H]^20+^: 896.2; [M+21H]^21+^: 853.5; [M+22H]^22+^: 814.8; [M+23H]^23+^: 779.4; [M+24H]^24+^: 747.0; [M+25H]^25+^: 717.1; [M+26H]^26+^: 689.6; Mass Found (ESI+) [M+15H]^15+^: 1195.1; [M+16H]^16+^: 1120.5; [M+17H]^17+^: 1054.6; [M+18H]^18+^: 996.1; [M+19H]^19+^: 943.7; [M+20H]^20+^: 896.5; [M+21H]^21+^: 853.9; [M+22H]^22+^: 815.1; [M+23H]^23+^: 779.7; [M+24H]^24+^: 747.3; [M+25H]^25+^: 717.4; [M+26H]^26+^: 691.4.


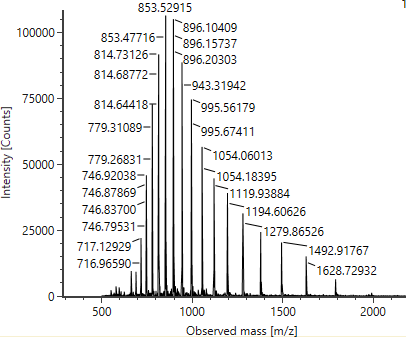

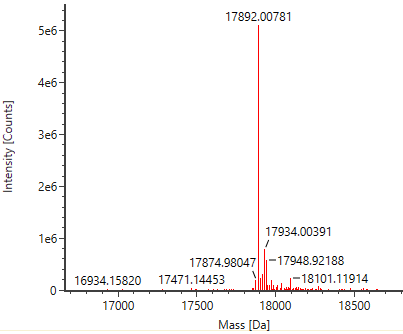


**Figure S60.** High-resolution MS (ESI) spectrum of purified **S20**, Calculated Mass for C_808_H_1229_N_217_O_233_S_6_ [M+H]^+^: 17892.93833; Mass Found (ESI+) [M+H]^+^: 17892.00078 (deconvoluted).

Fmoc-Lys(Boc)-OH pre-loaded resins (0.80 g, 0.53 mmol·g^-1^) were used to synthesize resin-bound peptide **S15** according to generally manual SPPS protocols described in **General Procedure A**. Allyl (All) protected Fmoc-Asp(All)-OH and pseudoproline dipeptide Fmoc-Tyr-Ser(ψ^Me,Me^Pro)-OH, Fmoc-Ala-Thr(ψ^Me,Me^Pro)-OH were introduced in the corresponding sites [6].

Resin-bound peptide was subjected to conservative cleavage with diluted TFA solution (1% in DCM, v/v) according to **General Procedure A** to give crude protected peptidyl hydrazide (3.11 g). The crude product was directly used in the next step without further purification.

The crude peptidyl hydrazide was dissolved in a mixture of THF (15 mL) and saturated NaHCO_3_ aqueous solution (8 mL), Boc_2_O (0.97 mL, 4.24 mmol, 10.0 equiv, based on initial loading value) were following added. The mixture was further stirred vigorously at 25 ºC for 6 hours. After the reaction completed, the reaction mixture was diluted with AcOEt (50 mL), the organic phase was seperated and dried over Na_2_SO_4_. After filtration, the solution was concentrated under reduced pressure to give crude protected peptide. The residue was further purified by silica gel column chromatography (DMC/MeOH, 40/1-20/1, v/v, with trace trimethylamine) to afford allyl protected peptide **S16** (1.46 g, 42% yield, based on initial loading value) as a white solid.


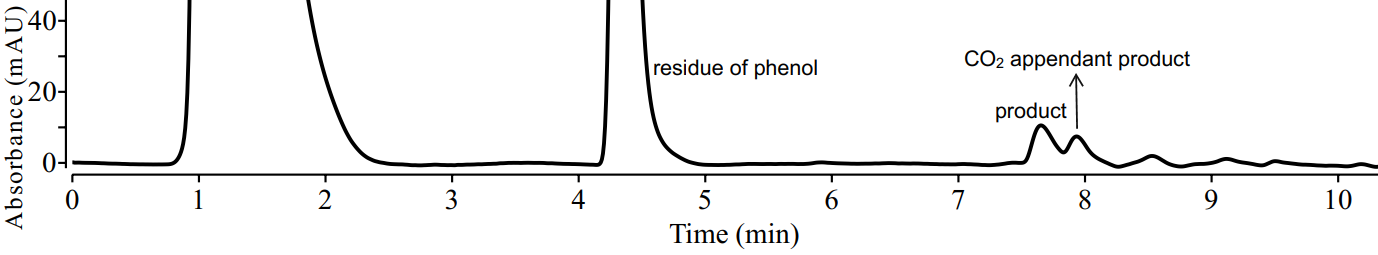


**
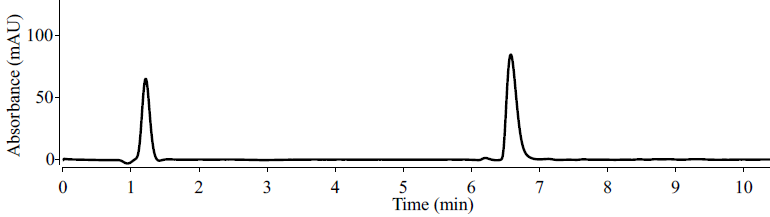
**

**Figure S61.** Analytical HPLC profile of crude (top) and purified (bottom) **S16** after globally deprotected with Cocktail B (*t*_R_ = 6.6 min, ReproSil-Pur 300Å C4 column, 2.1 × 100 mm, 3 μm, linear gradient 15%-50% of solvent B over 10 min).


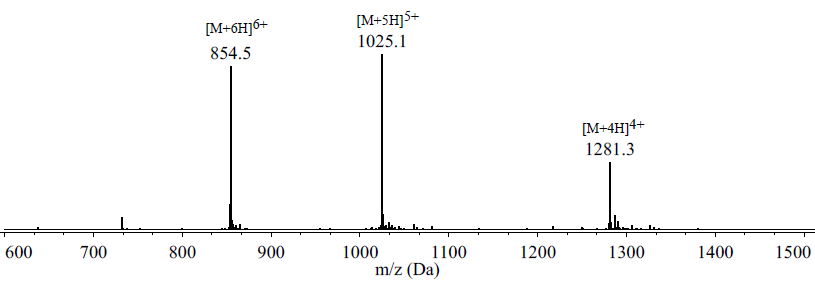


**Figure S62.** Low-resolution MS (ESI) spectrum of purified **S16** after globally deprotected with Cocktail B, Calculated Mass for C_240_H_346_N_60_O_62_S_2_ [M+4H]^4+^: 1282.9; [M+5H]^5+^: 1025.9; [M+6H]^6+^: 855.1; Mass Found (ESI+) [M+4H]^4+^: 1281.3; [M+5H]^5+^: 1025.1, [M+6H]^6+^: 854.5.

To a solution of allyl protected peptide **S16** (1.15 g, 0.14 mmol, 1.0 equiv) in DCM (7 mL), Pd(PPh_3_)_4_ (80.9 mg, 0.07 mmol, 0.5 equiv) and PhSiH_3_ (173 μL, 1.40 mmol, 10.0 equiv) were added. The mixture was kept stirring at 25 ºC for 1 h. During the progress, the reaction mixture turned from light yellow to dark brown. Upon completion, the reaction mixture was concentrated under reduced pressure, then further purified by silica gel column chromatography (DMC/MeOH, 30/1-15/1, v/v) to afford protected peptidyl acid **S17** (0.98 g, 86% isolated yield) as a gray solid.


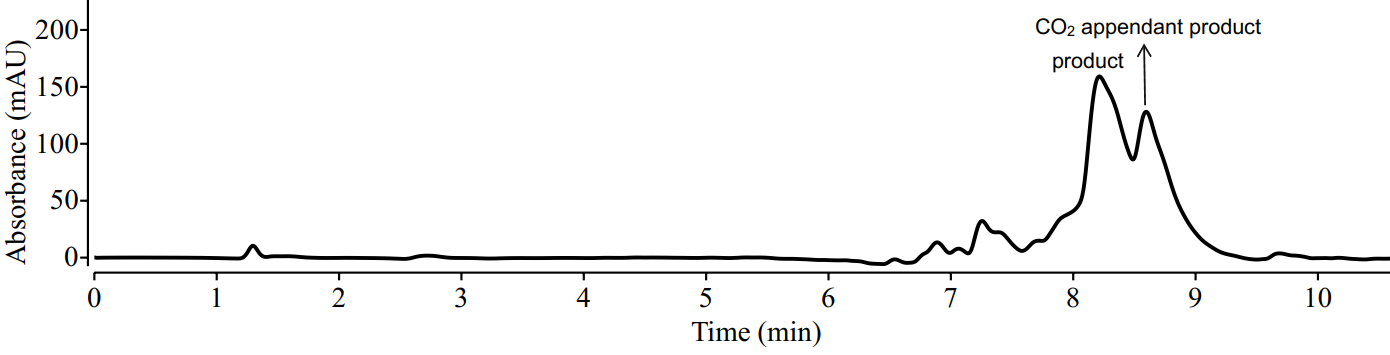


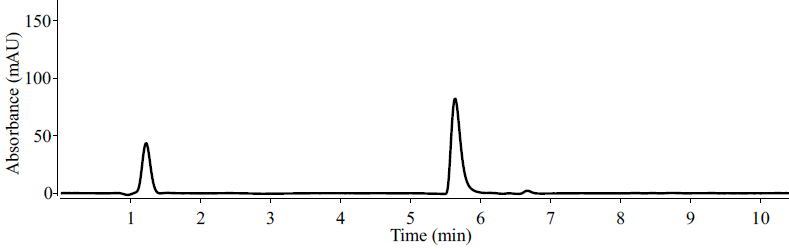


**Figure S63.** Analytical HPLC profile of crude (top) and purified (bottom) **S17** after globally deprotected with Cocktail B (*t*_R_ = 5.6 min, ReproSil-Pur 300Å C4 column, 2.1 × 100 mm, 3 μm, linear gradient 15%-50% of solvent B over 10 min).


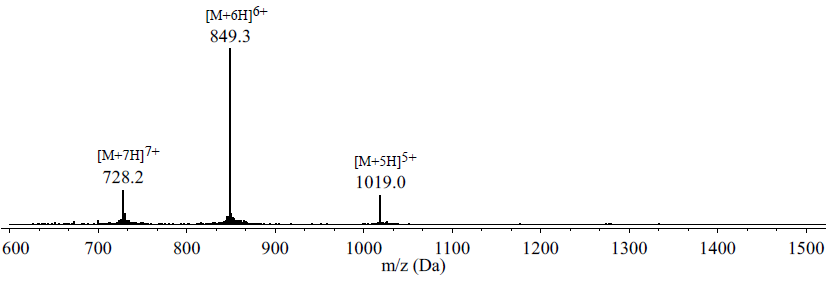


**Figure S64.** Low-resolution MS (ESI) spectrum of purified **S17** after globally deprotected with Cocktail B, Calculated Mass for C_237_H_342_N_60_O_62_S_2_ [M+5H]^5+^: 1018.6; [M+6H]^6+^: 849.0; [M+7H]^7+^: 727.8; Mass Found (ESI+) [M+5H]^5+^: 1019.0, [M+6H]^6+^: 849.3; [M+7H]^7+^: 728.2.

Protected peptidyl acid **S17** (50.0 mg, 6.1 μmol, 1.0 equiv) was glycosylated with nonasaccharide amine (13.1 mg, 8.0 μmol, 1.3 equiv) in the presence of HATU (4.64 mg, 12.2 μmol, 2.0 equiv), HOBt (1.73 mg, 12.8 μmol, 2.1 equiv) and DIPEA (4.2 μL, 24.4 μmol, 4.0 equiv) in anhydrous DMSO (80 μL). The reaction was kept stirring vigorously at 25 ºC for 3 hours. After the reaction completed, the protecting groups were globally cleaved by addition of Cocktail B according to **General Procedure A**. The crude product was purified by preparative RP-HPLC (Dubhe C18 column, 10 μm, 20 × 250 mm, linear gradient 30%-40% of solvent B over 30 min). Glycopeptide **S18** was obtained as a white lyophilized powder (18.4 mg, 45% isolated yield).


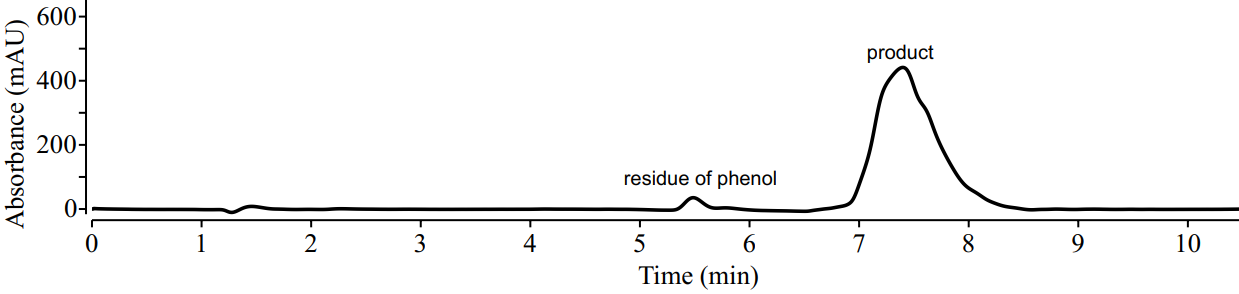


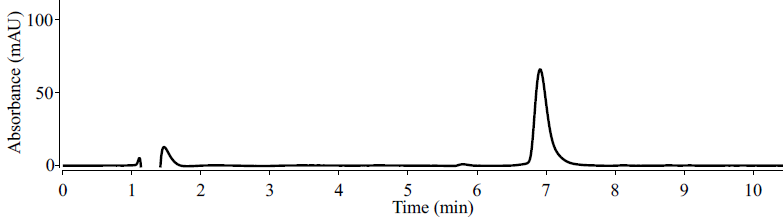


**Figure S65.** Analytical HPLC profile of crude (top) and purified (bottom) **S18** (*t*_R_ = 6.9 min, ReproSil-Pur 300Å C4 column, 2.1 × 100 mm, 3 μm, linear gradient 20%-50% of solvent B over 10 min).


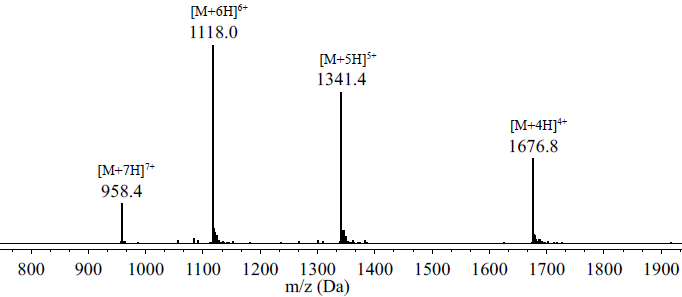


**Figure S66.** Low-resolution MS (ESI) spectrum of purified **S18**, Calculated Mass for C_299_H_445_N_65_O_106_S_2_ [M+4H]^4+^: 1677.5; [M+5H]^5+^: 1342.2; [M+6H]^6+^: 1118.7; [M+7H]^7+^: 959.0; Mass Found (ESI+) [M+4H]^4+^: 1676.8; [M+5H]^5+^: 1341.4; [M+6H]^6+^: 1118.0; [M+7H]^7+^: 958.4.

NCL between glycopeptidyl hydrazide **9** (2.52 mg, 0.67 μmol, 1.1 equiv) and *N*-terminal Cys glycopeptidyl hydrazide **S18** (4.08 mg, 0.61 μmol, 1.0 equiv) was conducted acccording to **General Procedure D** through two-step reaction in one-pot to assemble glycopeptidyl hydrazide **S19**. The resulting ligation mixture was purified by preparative RP-HPLC (Hedera C4 column, 10 μm, 20 × 250 mm, linear gradient 30%-40% of solvent B over 30 min). The glycopeptidyl hydrazide **S19** was obtained as a white lyophilized powder (4.17 mg, 66% isolated yield).


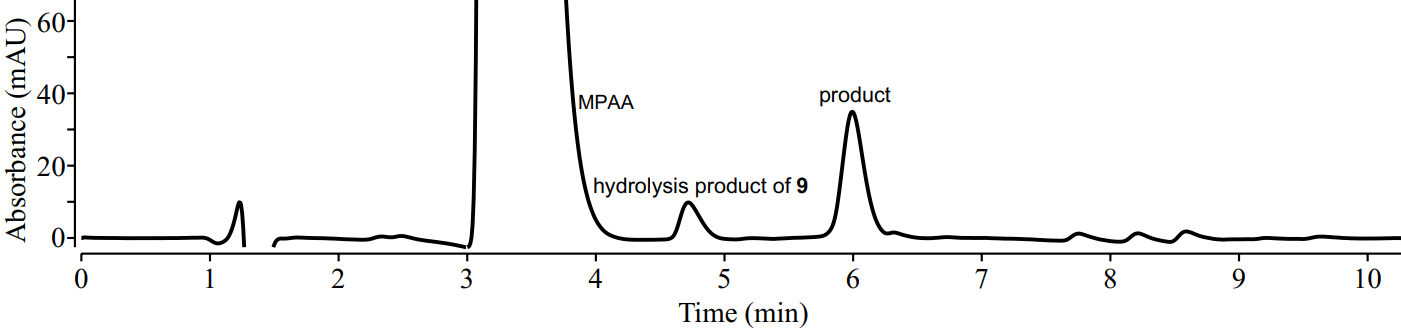


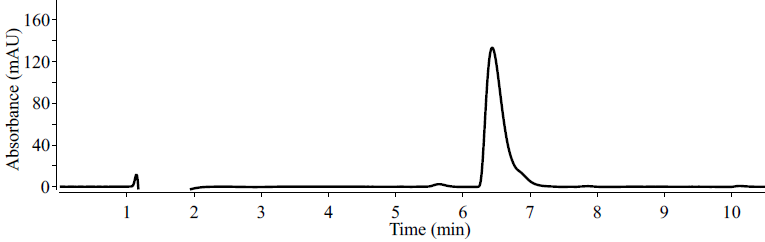


**Figure S67.** Analytical HPLC profile of crude (top) and purified (bottom) **S19** (*t*_R_ = 6.6 min, ReproSil-Pur 300Å C4 column, 2.1 × 100 mm, 3 μm, linear gradient 20%-60% of solvent B over 10 min).


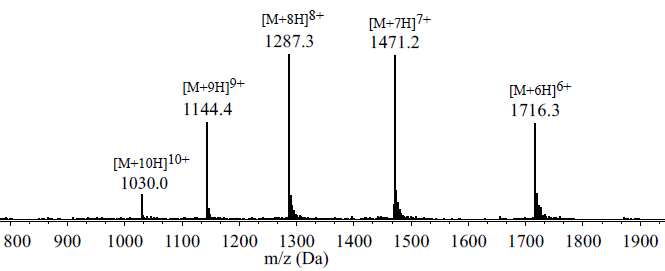


**Figure S68.** Low-resolution MS (ESI) spectrum of purified **S19**, Calculated Mass for C_299_H_445_N_65_O_106_S_2_ [M+6H]^6+^: 1716.5; [M+7H]^7+^: 1471.4; [M+8H]^8+^: 1287.6; [M+9H]^9+^: 1144.7; [M+10H]^10+^: 1030.3; Mass Found (ESI+) [M+6H]^6+^: 1716.3; [M+7H]^7+^: 1471.2; [M+8H]^8+^: 1287.3; [M+9H]^9+^: 1144.4; [M+10H]^10+^: 1030.0.

Full sequence of Omicron variant **S21** was assembled through NCL between glycopeptidyl hydrazide **S19** (2.47 mg, 0.24 μmol, 1.1 equiv) and *N*-terminal Cys recombinant fragment **S20** (3.90 mg, 0.22 μmol, 1.0 equiv) acccording to **General Procedure D** through two-step reaction in one-pot. The resulting ligation mixture was purified by preparative RP-HPLC (Hedera C4 column, 10 μm, 20 × 250 mm, linear gradient 32%-43% of solvent B over 30 min). The glycosylated full sequence **S21** was obtained as a white lyophilized powder (3.32 mg, 54% isolated yield).


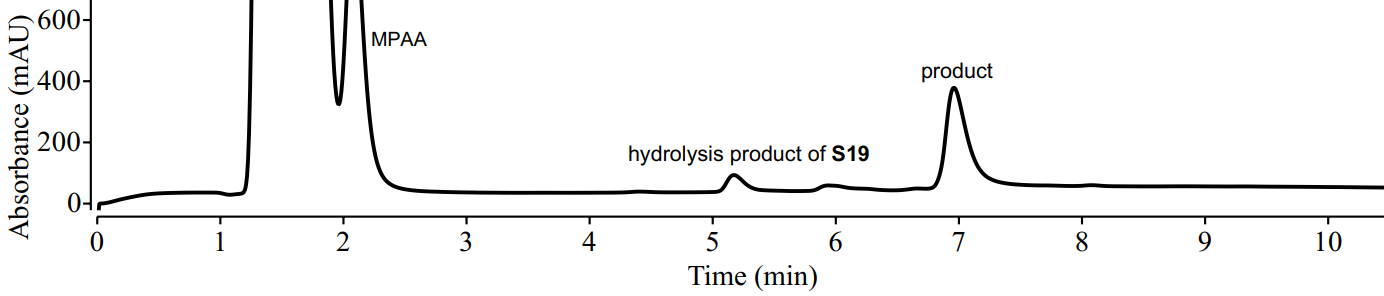


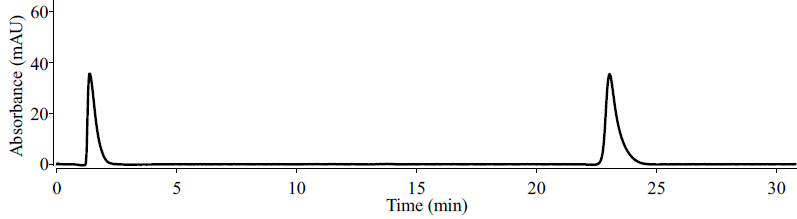


**Figure S69.** Analytical HPLC profile of crude (top) and purified (bottom) **S21** (*t*_R_ = 23.2 min, ReproSil-Pur 300Å C4 column, 2.1 × 100 mm, 3 μm, linear gradient 20%-40% of solvent B over 30 min).


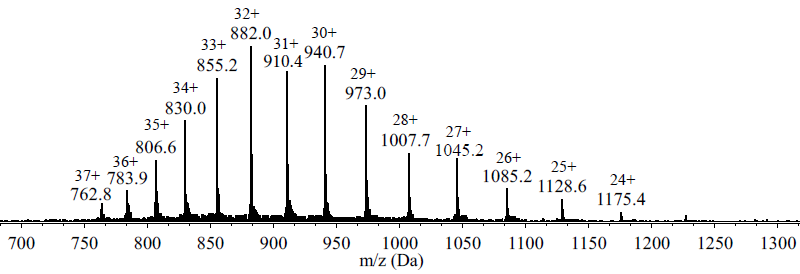


**Figure S70.** Low-resolution MS (ESI) spectrum of purified **S21**, Calculated Mass for C_1257_H_1916_N_310_O_409_S_8_: 28171.01 Da (molecular weight), [M+24H]^24+^: 1174.8; [M+25H]^25+^: 1127.9; [M+26H]^26+^: 1084.5; [M+27H]^27+^: 1044.4; [M+28H]^28+^: 1007.1; [M+29H]^29+^: 972.4; [M+30H]^30+^: 940.1; [M+31H]^31+^: 909.8; [M+32H]^32+^: 881.4; [M+33H]^33+^: 854.7; [M+34H]^34+^: 829.6; [M+35H]^35+^: 806.9; [M+36H]^36+^: 783.5; [M+37H]^37+^: 762.4; Mass Found (ESI+) [M+24H]^24+^: 1175.4; [M+25H]^25+^: 1128.6; [M+26H]^26+^: 1085.2; [M+27H]^27+^: 1045.2; [M+28H]^28+^: 1007.7; [M+29H]^29+^: 973.0; [M+30H]^30+^: 940.7; [M+31H]^31+^: 910.4; [M+32H]^32+^: 882.0; [M+33H]^33+^: 855.2; [M+34H]^34+^: 830.0; [M+35H]^35+^: 806.6; [M+36H]^36+^: 783.9; [M+37H]^37+^: 762.8.


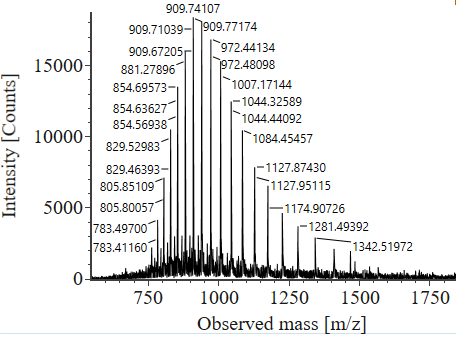

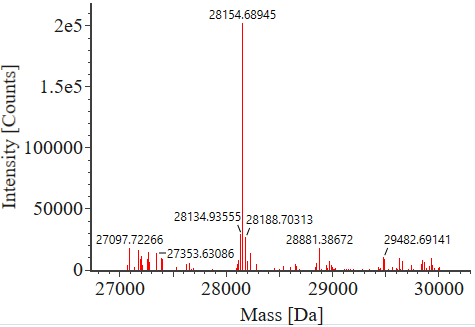


**Figure S71.** High-resolution MS (ESI) spectrum of purified **S21**, Calculated Mass for C_1257_H_1916_N_310_O_409_S_8_ [M+H]^+^: 28154.64828; Mass Found (ESI+) [M+H]^+^: 28154.68945 (deconvoluted).

Denatured **S21** (2.74 mg, 0.11 μM) was refolded and following purified by SEC-HPLC according to **General Procedure F**. The solution of Omicron RBD **5** in PBS (1 ×) was stocked for further characterization, the yield of folding was estimated to be 48% (0.89 mg·mL^-1^, 1.48 mL).


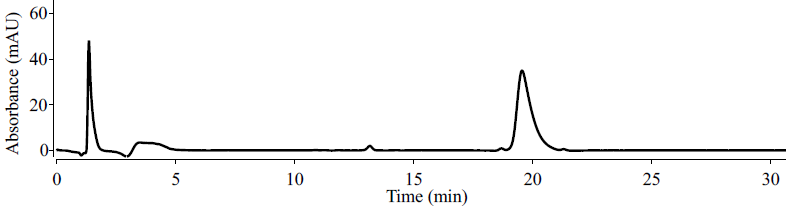


**Figure S72.** Analytical HPLC profile of purified Omicron RBD **5** (*t*_R_ = 19.5 min, ReproSil-Pur 300Å C4 column, 2.1 × 100 mm, 3 μm, linear gradient 20%-40% of solvent B over 30 min).

**Figure S73.** Low-resolution MS (ESI) spectrum of purified Omicron RBD **5**, Calculated Mass for C_1257_H_1908_N_310_O_409_S_8_ [M+15H]^15+^: 1878.5; [M+16H]^16+^: 1761.2; [M+17H]^17+^: 1657.6; [M+18H]^18+^: 1565.6; [M+19H]^19+^: 1483.3; [M+20H]^20+^: 1409.1; Mass Found (ESI+) [M+15H]^15+^: 1878.5; [M+16H]^16+^: 1761.2; [M+17H]^17+^: 1657.5; [M+18H]^18+^: 1565.6; [M+19H]^19+^: 1483.3; [M+20H]^20+^: 1409.1.

**Figure S74.** High-resolution MS (ESI) spectrum of purified Omicron RBD **5**, Calculated Mass for C_1257_H_1908_N_310_O_409_S_8_ [M+H]^+^: 28146.58568; Mass Found (ESI+) [M+H]^+^: 28146.48242 (deconvoluted).

## 8. Refolding, purification and characterization of naked RBD 6

**Scheme S9:** The synthesis of naked RBD **6**

Full nucleotides sequence (forward, the cleavage sites are marked in bold) and amino acids sequence of naked RBD (318-537) were listed as follow.

**CATATG**ATGAGGGTGCAGCCCACCGAGAGCATCGTGAGGTTCCCCAACATCACCAACCTGTGCCCCTTCGGCGTGTTCAACGCCACCAGGTTCGCCAGCGTGTACGCCTGGAACAGGAAGAGGATCAGCAACTGCGTGGCCGACTACAGCGTGCTGTACAACAGCGCCAGCTTCAGCACCTTCAAGTGCTACGGCGTGAGCCCCACCAAGCTGAACGACCTGTGCTTCACCAACGTGTACGCCGACAGCTTCGTGATCAGGGGCGACGAGGTGAGGCAGATCGCCCCCGGCCAGACCGGCAAGATCGCCGACTACAACTACAAGCTGCCCGACGACTTCACCGGCTGCGTGATCGCCTGGAACAGCAACAACCTGGACAGCAAGGTGGGCGGCAACTACAACTACCTGTACAGGCTGTTCAGGAAGAGCAACCTGAAGCCCTTCGAGAGGGACATCAGCACCGAGATCTACCAGGCCGGCAGCACCCCCTGCAACGGCGTGGAGGGCTTCAACTGCTACTTCCCCCTGCAGAGCTACGGCTTCCAGCCCACCAACGGCGTGGGCTACCAGCCCTACAGGGTGGTGGTGCTGAGCTTCGAGCTGCTGCACGCCCCCGCCACCGTGTGCGGCCCCAAGAAGAGCACCAACCTGGTGAAGAACAAG**CTCGAG**

**Figure S75.** Full nucleotide (top) and amino acid sequence (bottom) of full RBD sequence (M318-K537) **S22**

The plasmid pET21a-naked rbd was employed to recombine full RBD sequence **S22** according to the protocol described in **General Procedure B**. Supernatant containing **S22** was purified by preparative RP-HPLC (Hedera C4 column, 10 μm, 20 × 250 mm, linear gradient 28%-40% of solvent B over 30 min). Full sequance **S22** was obtained as a white lyophilized powder (26 mg·L^-1^, isolated yield).

**Figure S76.** Analytical HPLC profile of purified **S22** (*t*_R_ = 20.5 min, ReproSil-Pur 300Å C4 column, 2.1 × 100 mm, 3 μm, linear gradient 20%-45% of solvent B over 30 min).

**Figure S77.** Low-resolution MS (ESI) spectrum of purified **S22**, Calculated Mass for Calculated Mass for C_1118_H_1690_N_296_O_325_S_9_: 24765.75 Da (molecular weight), [M+14H]^14+^: 1768.9; [M+15H]^15+^: 1651.0; [M+16H]^16+^: 1547.9; [M+17H]^17+^: 1456.9; [M+18H]^18+^: 1376.0; [M+19H]^19+^: 1303.6; [M+20H]^20+^: 1238.5; [M+21H]^21+^: 1179.6; [M+22H]^22+^: 1126.0; [M+23H]^23+^: 1077.1; [M+24H]^24+^: 1032.3; [M+25H]^25+^: 991.0; [M+26H]^26+^: 952.9; [M+27H]^27+^: 917.7; [M+28H]^28+^: 884.9; [M+29H]^29+^: 854.4; [M+30H]^30+^: 826.0; [M+31H]^31+^: 799.4; Mass Found (ESI+) [M+14H]^14+^: 1769.1; [M+15H]^15+^: 1651.2; [M+16H]^16+^: 1548.0; [M+17H]^17+^: 1457.0; [M+18H]^18+^: 1376.1; [M+19H]^19+^: 1303.7; [M+20H]^20+^: 1238.6; [M+21H]^21+^: 1179.6; [M+22H]^22+^: 1126.0; [M+23H]^23+^: 1077.1; [M+24H]^24+^: 1032.2; [M+25H]^25+^: 991.0; [M+26H]^26+^: 952.9; [M+27H]^27+^: 917.7; [M+28H]^28+^: 885.0; [M+29H]^29+^: 854.5; [M+30H]^30+^: 826.1; [M+31H]^31+^: 799.5.

**Figure S78**. High-resolution MS (ESI) spectrum of purified **S22**, Calculated Mass for C_1118_H_1690_N_296_O_325_S_9_ [M+H]^+^: 24751.23642; Mass Found (ESI+) [M+H]^+^: 24751.20117 (deconvoluted).

Denatured **S22** (2.74 mg, 0.11 μM) was refolded and following purified by SEC-HPLC according to the protocols described in **General Procedure F**. The solution of naked RBD **6** in PBS (1 ×) was stocked for further characterization, the yield of folding was estimated to be 29% (0.54 mg·mL^-1^, 1.46 mL).

***Note*: During the refolding process of naked RBD 6, protein aggregated remarkably.**

**Figure S79.** Analytical HPLC profile of purified RBD **6** (*t*_R_ = 17.9 min, ReproSil-Pur 300Å C4 column, 2.1 × 100 mm, 3 μm, linear gradient 20%-45% of solvent B over 30 min).

**Figure S80.** Low-resolution MS (ESI) spectrum of purified RBD **6**, Calculated Mass for C_11118_H_1682_N_296_O_325_S_9_ [M+13H]^13+^: 1904.2; [M+14H]^14+^: 1768.3; [M+15H]^15+^: 1650.5; [M+16H]^16+^: 1547.4; [M+17H]^17+^: 1456.4; [M+18H]^18+^: 1375.6; [M+19H]^19+^: 1303.2; Mass Found (ESI+) [M+13H]^13+^: 1903.9; [M+14H]^14+^: 1767.9; [M+15H]^15+^: 1650.1; [M+16H]^16+^: 1547.0; [M+17H]^17+^: 1456.0; [M+18H]^18+^: 1375.1; [M+19H]^19+^: 1302.8.

**Figure S81.** High-resolution MS (ESI) spectrum of purified RBD **6**, Calculated Mass for C_1118_H_1682_N_296_O_325_S_9_ [M+H]^+^: 24743.17380; Mass Found (ESI+) [M+H]^+^:24743.16797 (deconvoluted).

## 9. CD and SDS-PAGE analysis of different RBD derivatives.

**Figure S82.** SDS-PAGE analysis of RBD **1**, **2**, **3**, **5** and **6** in a reduced condition. (SDS-PAGE analysis of RBD **4** was in consistent with that reported in our previous work [4])

The Circular Dichroism (CD) spectra of RBD **1**, **2**, **3**, **5**, **6** and HEK293 RBD were recorded on a Jasco J-1500 spectrometer in a semi-micro quartz cell at 25 ºC (data interval 0.2 nm, path length 0.1 cm, scan rate 100 nm/min, response time 2 s).

**Figure S83.** CD spectra of RBD **1**, **2**, **3**, **5**, **6** and HEK293 RBD. (CD spectra of RBD **4** was in consistent with that reported in our previous work [4])

## 10. Binding affinities of the RBD derivatives to antibodies and hACE2 receptor

To evaluate the binding affinities of the RBD derivatives to different antibodies and hACE2 receptor, Biacore 8K equipped with Protein A chip was applied to perform surface plasmon resonance (SPR) assays according to **General Procedure G** [4].

**Figure S84.** Real-time binding profiles between RBD derivatives and hACE2 receptor (1:1 binding，with the same protein gradient). Binding affinity *K_D_*, association rate constant *K_a_*, and dissociation constant *K_d_* were calculated and inserted.

**Figure S85.** Real-time binding profiles between RBD derivatives and CB6 antibody (1:1 binding, with the same protein gradient except RBD **5**), *K_D_*, *K_a_* and *K_d_* were inserted.

**Figure S86.** Real-time binding profiles between RBD derivatives and P2B-2F6 antibodies (1:1 binding), *K_D_*, *K_a_* and *K_d_* were inserted.

**Figure S87.** Real-time binding profiles between RBD derivatives and S309 antibodies (1:1 binding, with the same protein gradient), *K_D_*, *K_a_* and *K_d_* were inserted.

**Figure S88.** Real-time binding profiles between RBD derivatives and CR3022 antibodies (1:1 binding), *K_D_*, *K_a_* and *K_d_* were inserted.

# II. Immunological evaluation

**1. Materials and reagents**

Dulbecco’s modified Eagle’s medium (DMEM), bovine serum albumin (BSA), alum adjuvant (Alum) and fetal bovine serum (FBS) were purchased from Thermo Fisher Scientific. Firefly Luciferase Assay Kit and 3,3′-diaminobenzidine (DAB) were purchased from Promega and Dako, respectively. The antibodies used for immunohistochemistry staining were purchased from Servicebio. SARS-CoV-2 spike protein (His-tagged) was kindly provided by Prof. Tianlei Ying. Huh-7 cells were purchased from Cell Bank of the Chinese Academy of Sciences (Shanghai, China).

**2. Animals**

Female 6- to 8-week-old BALB/c mice were pruchased from Charles River Laboratories (CRL) (Beijing, China), and rasied in pathogen-free environment. Animal experiments were conducted at Laboratory Animal Centre of Shanghai Jiao Tong University (Shanghai, China). Female 6-8-week old pathogen-free, hACE2 transgenic mice (B6/JGpt-Ace2^em1Cin(hACE2-stop)^/Gpt) as a mild infection model were purchased from GemPharmatech (Nanjing, China), and kepted in pathogen-free environment.

**3. Ethics statements**

The Ethics Review Board of Shanghai Jiao Tong University and Kunming Institute of Zoology, Chinese Academy of Sciences (CAS) reviewed and approved approved the animal procedures (License No.:SYXK2018-0028, SMKX-2021-01-006). All the animal experiments were performed and approved by the Ethics Committee of Shanghai Jiao Tong University and Kunming Institute of Zoology, and were carried out in strict accordance with the animal ethics guidelines and the recommendations concerning laboratory animal welfare in the Guide for the Care and Use of Laboratory Animals of Shanghai Jiao Tong University and Kunming Institute of Zoology, CAS. The authentic SARS-CoV-2 virus challenge experiments were performed in the biosafety level-3 (BSL-3) laboratory of Kunming Institute of Zoology, CAS.

**4. BALB/c mice vaccination**

Forty-five pathogen-free female 6 to 8-week-old BALB/c mice were randomly allocated to 9 groups (group 0−8, n = 5/group). Each group was immunized subcutaneously with corresponding stimulator three times with two booster shot at 2-week intervals (Day 1, 15 and 29). Vaccine groups were vaccinated with the same doses of antigen (100 μL, containing equal mole relative to 10 μg HEK293 RBD) and Alum adjuvant (50 μg Alum). Placebo group 0 was immunized with 100 μL Alum/PBS (containing 50 μg Alum, without antigens). Groups 1−6 were administrated with 100 μL RBD **1−6** (equal mole relative to 10 mg HEK293 RBD) formulated with Alum adjuvant (50 μg Alum), respectively. Group 7 was immunized with a bivalent of RBD **4** & **5** (mole ratio 1/1) formulated with Alum adjuvant. Group 8 was immunized with 100 μL of HEK293 RBD (10 μg) formulated with Alum adjuvant (50 μg Alum). Physical state of the mice were regularly checked, including body weight change.Orbital venous blood was sampled on given days (Day 0, 14, 28, 35, 49 and 63), and sera samples were harvested by centrifugation (5000 rpm, 15 min, 4 ℃) after coagulating for 2 hours at 4 ℃. Sera samples were stocked in -80 ℃ for further use.

**Figure S89.** The immunization schedule for BLAB/c mice.

**Figure S90.** The body weight change of BLAB/c mice.

**5. Enzyme-linked immunosorbent assay (ELISA)**

Each well of 96-well ELSA microplates (Costartype 3690, Corning) were coated with 100 ng His-tagged HEK293 RBD overnight at 4 ℃. The antigen coated plates were washed three times with PBST (PBS + 0.05% Tween 20), then blocked with 5% bovine serum albumin (BSA) in PBS (100 μL/well) for 1 hour at 37 °C. After washing three times with PBST, the plates were incubated with 1:100 serially diluted sera samples (50 μL/well), following incubated for 1.5 hours at 37 ℃. Samples were performed in triplicate. The plates were washed three times with PBST, HRP-conjugated goat anti-mouse IgG (1:5000 dilution, 100 μL/well) (Sigma-Aldrich) was following added, further incubated for 1 hour at 37 ºC. Finally, the plates were washed five times with PBST and developed with 2, 2'-azino-bis(3-ethylbenzothiazoline-6-sulfonic acid) (ABTS, Invitrogen) substrate (50 μL/well). The absorbance at 405 nm (OD405) was recorded with a microplate reader (Thermo Fisher) after incubating at 25 ºC for 10 min. The absorbance intensity was expressed as *relative binding*.

**Figure S91.** Relative binding of vaccinee plasma against HEK293 RBD. Three independent experiments were performed in triplicate for every sample.

Log[dilution]/[OD405] was caculated, following plotted using Graphpad Prism software, and the curve was fitted with four parameters nonlinear regression. Then median effective dose (ED_50_) was calculated by the used of GraphPad Prism software.

**Figure S92.** Binding titers of vaccinee plasma (Day 35) against HEK293 RBD. Three independent experiments were performed in triplicate for every sample.

**6. Pseudovirus neutralization assay**

Huh-7 cells were seeded onto 96-well cell culture plates at 10000 cells per well, incubated in a humidified 5% CO_2_ atmosphere overnight at 37 °C. According to previous work [9], pseudo-viral infectivity was determined. Pseudovirus suspension of relative light unit (RLU) at 30000 was mixed with three-fold serially diluted antibody or sera samples (1/1, v/v, started from 10-fold diluted sera samples) for 1.5 hours at 37 ℃. The mixture was following added to the Huh-7 cells coated plate after washing with PBS, then incubated for another 12 hours. The medium was replaced by fresh DMEM medium containing 10% FBS, and further incubated for 48 hours. After washing with PBS for two times, lysis reagent (Promega) was added to the plates (50 μL/well) and the cell was lysed for 10 min at 25 ℃. Luciferase activity was measured by adding 30 μL of cell lysates to the substrate of Bright-Glo Firefly Luciferase Assay Kit (Promega). The RLU was recorded with a microplate reader. The percentage of inhibition was calculated as relative ratio reduction of RLU to that of the control (without sera sample) according to the following formula, (RLU^control^ - RLU^sample^) /RLU^control^. GraphPad Prism software was applied to data analysis and median inhibitory dose (ID_50_) caculation.

**Figure S93.** Neutralization titers of vaccinee plasma (Day 35) against WT SARS-CoV-2 pseudovirus. Three independent experiments were performed in triplicate for every sample.

**7. For transgenic mouse vaccination and virus challenge study**

Twelve female 6 to 8-week-old hACE2 transgenic mice (B6/JGpt-Ace2^em1Cin(hACE2-stop)^/Gpt, mild infection model) were randomly allocated to 4 groups (group 0-3, n = 3/group). The mice were immunized intramuscularly with PBS (group 0), HEK293 RBD (group 1), RBD **1** (group 2) and RBD **2** (group 3) formulated with Alum adjuvant three times with two booster shot at 2-week intervals (Day 1, 15 and 29). Vaccine groups were vaccinated with the same doses of antigen (100 μL, containing equal mole relative to 10 μg HEK293 RBD) and Alum adjuvant (50 μg Alum). Placebo group 0 was immunized with 100 μL Alum/PBS (containing 50 μg Alum, without antigens). Orbital venous blood was sampled and sera samples were collected before virus challenge (Day 35). At one week after the 2^nd^ booster, the mice were intranasally challenged with 1 × 10^6^ TCID_50_ of WT SARS-CoV-2 in 30 µL volume (Guangdong Provincial Center for Disease Control and Prevention, Guangzhou, China). Physical state of the mice were regularly checked everyday, including body weight change. All mice were euthanized at 3 days post-infection (dpi), viral RNA levels in the lungs were determined by real-time RT-PCR as described previously [9,10].

**Figure S94.** The immunization and authentic SARS-CoV-2 infection schedule for hACE2 transgeic mice.

**Figure S95.** The body weight change of hACE2 transgeic mice post authentic SARS-CoV-2 infection. (dpi, days post-infection).

**Figure S96.** Binding titers of vaccinee plasma (Day 35, hACE2 transgeic mice) against HEK293 RBD. Three independent experiments were performed in triplicate for every sample.

**Figure S97.** Lung virus loads of challenged hACE2 transgenic mice. Three independent experiments were performed in triplicate for every sample.

**8. Histopathology and immunohistochemistry (IHC) staining**

All hACE2 transgenic mice were euthanized at 3 dpi, fresh lung tissues were harvested and fixed in 10% paraformaldehyde for 24 hours, following immersed in 70% ethanol for 72 hours. Subsequently, the Specimens were embedded in paraffin and cut into a 5 mm thick slices by a microtome.

For histological staining, tissue slices were stained with hematoxylin and eosin (H&E), and whole slide imaging was observed with a light microscope. Histopathologic changes were evaluated by a histopathologist in a blinded manner.

For IHC staining, the tissue slices were deparaffinized with xylene and followed by rehydrated with graded ethanol. Antigen retrieval was performed by heating the tissue slices in sodium citrate buffer (10 mM, pH 6.0) at 100 ℃ for 10 min in a microwave oven. After cooling at room temperature, the tissue slices were washed three times with PBS by shaking for 5 min. 3% H_2_O_2_ solution in methanol was applied to block endogenous peroxidase activity in the darkness for 30 min, followed by washing three times with PBS by shaking for 5 min. Tissue slices were blocked with 3 % BSA in PBS (1 ×) for 30 min at 25 ℃. After carefully discarding the blocking buffer, tissue slices were incubated with PBS diluted primary antibody, anti-CD8 (1:800) (Servicebio), further incubated overnight at 4 ℃. Tissue slices was washed three times with PBS by shaking for 5 min, and PBS diluted goat anti-rabbit HRP secondary antibody (1:200) (Servicebio) was added, incubated for another 1 hour at 25 ℃. After washing three times with PBS, freshly prepared DAB was applied to visualise. Real-time monitor the staining under microscope and the process was stopped by rinsing with water. Furtherly, tissue slices were counterstained with hematoxylin and visualized under microscope. CD8 staining were photographed with a light microscope, the results were analyzed with Image-Pro Plus 6.0 software and caculated positive ratio and density of CD8.

**Figure S98.** Lung tissue histopathology H&E staining and immunohistochemistry of authentic SARS-CoV-2 infected mice.

**Figure S 99.** CD8 positive density (left) and ratio (right) of lung tissue from authentic SARS-CoV-2 infected mice

**9. Statistical analysis.**

GraphPad Prism 8.0 software was applied to biological data analysis, and all data was presented as geometric means ± standard deviation (SD). Statistical significance of multiple groups comparison was performed using Student’s t-test, one-way ANOVA with Tukey’s multiple comparison test. Statistical significance values (P) were presented as asterisks (*P < 0.05; **P < 0.01; ***P < 0.001; ****P < 0.0001), and P < 0.05 was considered statistically significant difference.

**Reference**

1. Eissler S, Kley M, Bächle D *et al.* Substitution determination of Fmoc‐substituted resins at different wavelengths. *J Pept Sci* 2017; **23**: 757-62.

2. Reif A, Lam K, Weidler S *et al.* Natural Glycoforms of Human Interleukin 6 show atypical plasma clearance. *Angew Chem Int Ed* 2021; **60**: 13380-87.

3. Fang G M, Li Y M, Shen F *et al.* Protein chemical synthesis by ligation of peptide hydrazides. *Angew Chem Int Ed* 2011; **33**: 7645-49.

4. Ye F, Zhao J, Xu P *et al.* Synthetic Homogeneous Glycoforms of the SARS‐CoV‐2 Spike Receptor‐Binding Domain Reveals Different Binding Profiles of Monoclonal Antibodies. *Angew Chem Int Ed* 2021; **60**: 12904-10.

5. Yang J, Wang W, Chen Z *et al.* A vaccine targeting the RBD of the S protein of SARS-CoV-2 induces protective immunity. *Nature* 2020; **586**: 572-77.

6. Wang P, Aussedat B, Vohra Y *et al.* An advance in the chemical synthesis of homogeneous N‐linked glycopolypeptides by convergent aspartylation. *Angew Chem Int Ed* 2012; **51**: 11571-75.

7. Wang P, Dong S, Shieh J, Danishefsky, S J *et al.* Erythropoietin derived by chemical synthesis. *Science* 2013; **342**: 1357-60.

8. Ullmann V, Rädisch M, Boos I *et al.* Convergent solid‐phase synthesis of N‐glycopeptides facilitated by pseudoprolines at consensus‐sequence Ser/Thr residues. *Angew Chem Int Ed* 2012; **51**: 11566-70.

9. Li C, Zhan W, Yang Z *et al.* Broad neutralization of SARS-CoV-2 variants by an inhalable bispecific single-domain antibody. *Cell* 2022; **185**: 1389-401.

10. Pang W, Lu Y, Zhao Y-B *et al.* A variant-proof SARS-CoV-2 vaccine targeting HR1 domain in S2 subunit of spike protein. *Cell Res* 2022; **32**: 1068-85.
